# Advances in targeted therapy for malignant lymphoma

**DOI:** 10.1038/s41392-020-0113-2

**Published:** 2020-03-06

**Authors:** Li Wang, Wei Qin, Yu-Jia Huo, Xiao Li, Qing Shi, John E. J. Rasko, Anne Janin, Wei-Li Zhao

**Affiliations:** 1 State Key Laboratory of Medical Genomics, Shanghai Institute of Hematology, Shanghai Rui Jin Hospital, Shanghai Jiao Tong University School of Medicine, 197 Rui Jin Er Road, Shanghai, China; 2 Pôle de Recherches Sino-Français en Science du Vivant et Génomique, Laboratory of Molecular Pathology, Shanghai, China; 3 Gene & Stem Cell Therapy Program Centenary Institute, Sydney Medical School, University of Sydney, Camperdown, Australia; 4 Cell and Molecular Therapies, Royal Prince Alfred Hospital, Camperdown, Australia; 5 U1165 Inserm/Université Paris 7, Hôpital Saint Louis, Paris, France

**Keywords:** Haematological cancer, Cancer therapy

## Abstract

The incidence of lymphoma has gradually increased over previous decades, and it ranks among the ten most prevalent cancers worldwide. With the development of targeted therapeutic strategies, though a subset of lymphoma patients has become curable, the treatment of refractory and relapsed diseases remains challenging. Many efforts have been made to explore new targets and to develop corresponding therapies. In addition to novel antibodies targeting surface antigens and small molecular inhibitors targeting oncogenic signaling pathways and tumor suppressors, immune checkpoint inhibitors and chimeric antigen receptor T-cells have been rapidly developed to target the tumor microenvironment. Although these targeted agents have shown great success in treating lymphoma patients, adverse events should be noted. The selection of the most suitable candidates, optimal dosage, and effective combinations warrant further investigation. In this review, we systematically outlined the advances in targeted therapy for malignant lymphoma, providing a clinical rationale for mechanism-based lymphoma treatment in the era of precision medicine.

## Introduction

Lymphoma is the most common lymphoid malignancy and is among the ten most prevalent cancers worldwide.^1^ Lymphoma is a heterogeneous entity and includes Hodgkin’s lymphoma (HL) and non-Hodgkin’s lymphoma (NHL). HL accounts for 10–15% of lymphoma and is characterized by the presence of Reed–Sternberg cells. NHL accounts for 80–85% of lymphoma, including B-cell NHLs (B-NHLs) expressing CD20 or CD19, T-cell NHLs (T-NHLs) expressing CD3, CD4, or CD8, and natural killer (NK)/T-cell NHLs expressing CD56. Chemotherapy is the standard of care for lymphoma patients. The introduction of monoclonal antibodies targeting surface antigens has greatly changed the therapeutic landscape of lymphoma. For example, rituximab, an anti-CD20 antibody targeting CD20 in B-NHLs and brentuximab vedotin targeting CD30 in classical HL and T-NHLs, have significantly improved the response rates and clinical outcomes of patients.^2,3^ In addition, growing insights into molecular biology and signaling pathways have led to the development of many innovative agents for lymphoma in recent years.^4^ More recently, with a better understanding of the crosstalk between malignant lymphocytes and the tumor microenvironment, chimeric antigen receptor T-cells (CAR-T cells) have been rapidly developed in treating relapse and refractory patients.^5,6^ Although the overall survival (OS) of lymphoma patients has been considerably improved by the new immunochemotherapeutic regimens, the selection of targeted agents and the optimal dosage are important due to treatment-related adverse events (AEs). In this review, we systematically outlined the advances in targeted therapy for malignant lymphoma that provide significant improvement in mechanism-based lymphoma treatment in the era of precision medicine.

## Surface antigens and targeted therapies

Surface antigens are the most accessible part of lymphoma cells, and monoclonal antibodies (mAbs) targeting surface antigens have become important therapeutic strategies in many lymphoid malignancies. Cytotoxic to tumor cells, mAbs relatively spare normal tissues. The mechanisms of action include the induction of apoptosis, antibody-dependent cellular cytotoxicity (ADCC) and complement-dependent cytotoxicity (CDC). In addition to “bare” antibodies, antibodies or their fragments may be linked with cell toxins, immunotoxins, or radioisotopes to increase clinical efficacy.

### CD20

The CD20 molecule is a transmembrane protein involved in B-cell activation and differentiation and is present on all mature B-cells and most B-NHL cells.^7^ Moreover, without internalization or downregulation following antibody binding, CD20 functions as an ideal therapeutic target for most B-NHLs.^8^ Moreover, pro-B cells and antibody-producing plasma cells do not express CD20, so anti-CD20 treatment will not impair the healthy B-cell population.

Anti-CD20 mAbs are classified as type I and type II.^9^ Type I antibodies most effectively induce CDC, in which the binding of the mAb activates a complement cascade. Type I antibodies also induce ADCC, in which immune cells expressing Fc gamma receptor (FcγR) attack antibody-coated cells. Type II antibodies initiate ADCC as well as cell death through apoptotic or non-apoptotic mechanisms.

Rituximab was the first mAb to target CD20 and the first mAb approved to treat cancer patients. It is a chimeric antibody with a murine variable region and a human IgG1-kappa constant region,^8^ classified as a type I mAb. The significant anti-lymphoma activity of rituximab in early trials^3,10–12^ has led to its widespread use in most CD20^+^ B-NHLs.

The targeted agents and clinical trials related to mAbs are listed in Table 1. Ofatumumab is a fully humanized second-generation type I CD20 antibody that exhibits more potent CDC than rituximab in vitro.^13^ Ofatumumab is approved in combination with chlorambucil for chronic lymphocytic leukemia (CLL).^14,15^ Moreover, the results from a phase 2 trial (NCT00410163) suggested that ofatumumab in combination with fludarabine and cyclophosphamide was efficient in untreated CLL patients.^16^ The main AEs were infusion-related reactions and grade 1–2 infections.

**Table 1:** Targeted agents and clinical trials related to monoclonal antibodies

Drug	Disease	Trial name	Phase	Status	ORR/CR	NCT#	Reference
*Anti-CD20 antibody*							
*Ofatumumab*	*A fully humanized second-generation type I CD20 antibody*						
Ofatumumab, fludarabine, cyclophosphamide	CLL	Ofatumumab with fludarabine and cyclophosphamide in b-cell chronic lymphocytic leukemia patients	2	Completed	500 mg, 77%/42%; 100 mg, 73%/50%	NCT00410163	^16^
*Obinutuzumab*	*A humanized type II CD20 antibody*						
Obinutuzumab	Relapsed or refractory DLBCL/MCL	A dose-escalating study of obinutuzumab in patients with b-lymphocyte antigen (CD20^+^) malignant disease (gauguin)	1/2	Completed	DLBCL, 28%/4%; MCL, 27%/13%	NCT00517530	^19^
Obinutuzumab, bendamustine vs. bendamustine	Rituximab-refractory iNHLs	A study to investigate the efficacy and safety of bendamustine compared with bendamustine plus obinutuzumab in participants with rituximab-refractory, indolent non-Hodgkin’s lymphoma (GADOLIN)	3	Completed	Obinutuzumab plus bendamustine, 69%/11%; bendamustine monotherapy, 63%/12%	NCT01059630	^20^
Obinutuzumab, CHOP/CVP/bendamustine vs. rituximab, CHOP/CVP/bendamustine	Untreated iNHLs	A study of obinutuzumab (RO5072759) plus chemotherapy in comparison with rituximab plus chemotherapy followed by obinutuzumab or rituximab maintenance in patients with untreated advanced indolent non-Hodgkin’s lymphoma (GALLIUM)	3	Active, not recruiting	FL: obinutuzumab group, 88.5%/19.5%; rituximab group, 86.9%/23.8%	NCT01332968	^21^
Obinutuzumab, CHOP/FC/bendamustine	FL	A study of obinutuzumab in combination with chemotherapy in participants with CD20^+^ B-cell follicular non-Hodgkin’s lymphoma	1	Completed	G-CHOP, 96%/39%; G-FC, 93%/50%	NCT00825149	^22^
G-Clb vs. Clb vs. R-Clb	Untreated CLL	CLL11: a study of obinutuzumab with chlorambucil in patients with previously untreated chronic lymphocytic leukemia (Stage 1a)	3	Completed	G-Clb, 77.3%/22.3%; Clb, 31.4%/0%; R-Clb, 65.7%/7.3%	NCT01010061	^23^
Obinutuzumab, ibrutinib vs. obinutuzumab, chlorambucil	Untreated CLL/SLL	A multicenter study of ibrutinib in combination with obinutuzumab versus chlorambucil in combination with obinutuzumab in patients with treatment naïve CLL or SLL	3	Completed	Obinutuzumab plus ibrutinib, 91%/41%; obinutuzumab plus chlorambucil, 81%/16%	NCT02264574	^24^
*Ublituximab*	*A type I, chimeric, recombinant IgG1 monoclonal antibody targeting a unique epitope on the CD20 antigen, glycoengineered to enhance affinity for all FcRIIIa variants*						
Ublituximab, ibrutinib	CLL/MCL	Ublituximab plus ibrutinib in select B-cell malignancies	1/2	Completed	88%/5%	NCT02013128	^27^
Ublituximab, ibrutinib vs. ibrutinib	Previously treated high-risk CLL	Ublituximab in combination with ibrutinib versus ibrutinib alone in patients with previously treated high-risk chronic lymphocytic leukemia	3	Active, not recruiting	combination arm, 78%/7%; monotherapy, 45%/0%	NCT02301156	^28^
Ublituximab, umbralisib vs. obinutuzumab, chlorambucil	CLL	Ublituximab plus umbralisib compared to obinutuzumab plus chlorambucil in patients with untreated and previously treated chronic lymphocytic leukemia	3	Active, not recruiting	–	NCT02612311	–
Ublituximab, umbralisib; ublituximab, umbralisib, ibrutinib	B-NHLs, CLL	Ublituximab in combination with umbralisib +/− ibrutinib or bendamustine in patients with B-cell malignancies	1	Completed	Ublituximab, umbralisib, ibrutinib, 84%/30%; ublituximab, umbralisib, 46%/17%	NCT02006485	^29,30^
*Veltuzumab*	*A humanized type I anti-CD20 monoclonal antibody*						
Veltuzumab	Relapsed or refractory B-NHLs	Study of humanized anti-CD20 in patients with CD20^+^ non-Hodgkin’s lymphoma	1/2	Completed	FL, 44%/27%; MZL, 83%/33%; DLBCL, 43%/0%	NCT00285428	^31^
*Ocrelizumab*	*A humanized type I anti-CD20 monoclonal antibody*						
Ocrelizumab	Relapsed or refractory FL	An open-label, multicentre, dose-escalating phase 1/2 trial of 3-weekly ocrelizumab in patients with follicular non-Hodgkin’s lymphoma	1/2	Completed	38%/15%	NCT02723071	^32^
*CT-P10*	*A rituximab biosimilar*						
CT-P10 vs. rituximab	FL	To compare efficacy and safety between CT-P10 and rituxan in patients with low tumor burden follicular lymphoma	3	Active, not recruiting	CT-P10, 83%/28%; rituximab, 81%/34%	NCT02260804	^33^
CT-P10, CVP vs. R-CVP	FL	To demonstrate equivalence of pharmacokinetics and noninferiority of efficacy for CT-P10 in comparison with rituxan	3	Completed	CT-P10, CVP, 97%/30%; R-CVP, 93%/22%	NCT02162771	^34^
*GP2013*	*A rituximab biosimilar*						
GP2013, CVP vs. R-CVP	Untreated advanced-stage FL	GP2013 in The treatment of patients with previously untreated, advanced-stage follicular lymphoma	3	Completed	GP2013, CVP, 87%/15%; R-CVP, 88%/13%	NCT01419665	^35^
*PF-05280586*	*A rituximab biosimilar*						
PF-05280586 vs.rituximab	FL	A study of PF-05280586 (Rituximab-Pfizer) or MabThera® (Rituximab-EU) for the First-Line treatment of patients with CD20^+^, low tumor burden, follicular lymphoma (REFLECTIONS B328-06)	3	Completed	PF-05280586, 76%/26%; rituximab, 71%/28%	NCT02213263	^36^
*ABP798*	*A rituximab biosimilar*						
ABP798 vs. rituximab	B-NHLs	Study to assess if ABP798 Is safe and effective in treating non-Hodgkin’s lymphoma compared to rituximab	3	completed	NA	NCT02747043	–
^*90*^*Y-ibritumomab tiuxetan*	*A radiolabeled anti-CD20 monoclonal antibody which targets the same epitope on the CD20 molecule like rituximab and chelates the radioactive particle Yttrium-90*						
^90^Y-ibritumomab tiuxetan	FL	^90^Y-Ibritumomab tiuxetan first line in follicular lymphoma	2	Unknown status	87%/56%	NCT00772655	^41^
^90^Y-ibritumomab tiuxetan	FL	Phase 2 study of fractionated ^90^Y-ibritumomab tiuxetan radioimmunotherapy as an initial therapy of follicular lymphoma	2	Completed	95.8%/69.4%	NCT01493479	^42^
^90^Y-ibritumomab tiuxetan vs. no treatment	FL	Treatment with ^90^Y-ibritumomab tiuxetan versus no treatment in patients with follicular non-Hodgkin’s lymphoma (stage III or IV) having achieved a partial or complete pemission after first line chemotherapy	3	Completed	PR after induction therapy converted to a CR/CRu: consolidation arm, 77%; control arm, 17.5%	NCT00185393	^43,44^
^90^Y-ibritumomab tiuxetan, rituximab vs. rituximab	Untreated FL	Rituximab with or without ^90^Y-Ibritumomab tiuxetan in treating patients with untreated follicular lymphoma	3	Recruiting	–	NCT02320292	–
^90^Y-ibritumomab tiuxetan vs. ASCT	Relapsed or refractory FL	A phase 3 multicenter, randomized study comparing ^90^Y-Ibritumomab tiuxetan vs. ASCT in patients with relapsed or refractory FL	3	Recruiting	–	NCT01827605	–
^90^Y-ibritumomab tiuxetan, BEAM	FL/DLBCL/MCL/transformed lymphomas	Phase 2 trial of a transplantation regimen of ^90^Y-Ibritumomab tiuxetan and high-dose chemotherapy in patients with non-Hodgkin’s lymphoma	2	Completed	NA	NA	^45^
*Anti-CD22 antibody*							
*Epratuzumab*	*A humanized IgG1 monoclonal antibody targeting CD22*						
Epratuzumab	Relapsed or refractory iNHLs	Phase 1/2 trial of epratuzumab in indolent non-Hodgkin’s lymphoma	1/2	Completed	all, 18%/6%; FL, 24%/8%	NA	^52^
Epratuzumab	Relapsed or refractory aggressive NHLs	Phase 1/2 trial of epratuzumab in patients with recurrent aggressive NHLs	1/2	Completed	all, 10%/6%; DLBCL, 15%/9%	NA	^53^
Epratuzumab, rituximab	Relapsed or refractory iNHLs	Phase 2 trial of rituximab plus epratuzumab in patients with relapsed or refractory, indolent non-Hodgkin’s lymphoma	2	Completed	FL, 54%/24%; SLL, 57%/43%	NA	^54^
Epratuzumab, rituximab	Untreated FL	Epratuzumab and rituximab in treating patients with previously untreated follicular non-Hodgkin’s lymphoma	2	Completed	88.2%/42.4%	NCT00553501	^55^
Epratuzumab, R-CHOP	DLBCL	Monoclonal antibody therapy and combination chemotherapy in treating patients with stage II, stage III, or stage IV diffuse large B-cell lymphoma	2	Completed	96%/74%	NCT00301821	^56^
*Inotuzumab*	*A CD22-targeted ADC combining a humanized IgG4 anti-CD22 monoclonal antibody with calicheamicin, an enediyne antibiotic*						
Inotuzumab ozogamicin, rituximab	B-NHLs	Study evaluating inotuzumab ozogamicin administered in combination with rituximab in subjects with non-Hodgkin’s lymphoma	1/2	Completed	Relapsed FL, 87%/62%; relapsed DLBCL, 74%/50%; refractory aggressive NHLs, 20%/3%	NCT00299494	^60^
R-InO vs. RB/RG	Relapsed or refractory aggressive NHLs	A study of inotuzumab ozogamicin plus rituximab for relapsed or refractory aggressive non-Hodgkin’s lymphoma patients who are not candidates for intensive high-dose chemotherapy	3	Terminated	R-InO, 41%/13%; RB/RG, 44%/13%;	NCT01232556	^61^
Inotuzumab ozogamicin, R-CVP vs. R-G-CVP	DLBCL	Treatment of patients with diffuse large B-cell lymphoma who are not suitable for anthracycline containing chemotherapy	2	Active, not recruiting	–	NCT01679119	–
*Moxetumomab pasudotox*	*A recombinant immunotoxin consisting of the Fv portion of the anti-CD22 antibody and a fragment of pseudomonas exotoxin A*						
Moxetumomab pasudotox	Relapsed or refractory HCL	Safety study of moxetumomab pasudotox in patients with HCL with advance disease	1	Unknown	86%/46%	NCT00462189	^64^
Moxetumomab pasudotox	Relapsed or refractory HCL	Moxetumomab pasudotox for advanced HCL	3	Completed	75%/41%	NCT01829711	^65^
*Anti-CD30 antibody*							
*SGN-30*	*A chimeric monoclonal antibody consisting of the variable region of an anti-CD30 murine monoclonal antibody with human gamma 1 heavy chain and kappa light chain constant regions*						
SGN-30	Relapsed or refractory HL/ALCL	Phase 2 study of SGN-30 in Hodgkin’s lymphoma or systemic anaplastic large cell lymphoma	2	Completed	ALCL, 17%/5%; HL, 0%/0%	NA	^74^
SGN-30, GVD vs. placebo, GVD	Relapsed or refractory classical HL	Phase 2 trial of SGN-30 or placebo with GVD in patients with relapsed or refractory classical HL	2	Terminated	SGN-30, GVD, 65%/NA; GVD, 57%/NA	NA	^75^
*BV*	*A CD30 ADC connecting an anti-CD30 antibody with the anti-mitotic agent MMAE via a valine-citrulline peptide linker*						
BV	HL/ALCL	Phase 1 open-label dose finding study of brentuximab vedotin for CD30^+^ hematologic malignancies	1	Completed	38%/27%	NCT00430846	^2^
BV	HL	A pivotal open-label Trial of brentuximab vedotin for Hodgkin’s lymphoma	2	Completed	75%/34%	NCT00848926	^79^
BV	ALCL	A phase 2 open-label trial of brentuximab vedotin for systemic anaplastic large cell lymphoma	2	Completed	86%/57%	NCT00866047	^80^
BV	Relapsed or refractory NHLs	A study of brentuximab vedotin in relapsed or refractory non-Hodgkin’s lymphoma	2	Completed	T-NHLs, 41%/24%	NCT01421667	^81^
BV vs. methotrexate/bexarotene	CD30^+^ CTCL	A phase 3 trial of brentuximab vedotin versus physician’s choice (methotrexate or bexarotene) in participants with CD30^+^ cutaneous T-cell lymphoma (ALCANZA study)	3	Completed	BV, 56%/16%; methotrexate/bexarotene, 13%/2%	NCT01578499	^83^
BV, AVD vs. ABVD	Advanced classical HL	A frontline therapy trial in participants with advanced classical Hodgkin’s lymphoma	3	Active, not recruiting	A+AVD, 86%/73%; ABVD, 83%/70%	NCT01712490	^84^
BV, CHP, CHOP	CD30^+^ mature T-cell and NK-cell neoplasms	A phase 1 study of brentuximab vedotin given sequentially and combined with multi-agent chemotherapy for CD30^+^ mature T-cell and NK-cell neoplasms	1	Completed	sequential treatment, 85%/62%; combination treatment, 100%/88%	NCT01309789	^85,86^
BV, CHP vs. CHOP	CD30^+^ mature T-cell lymphomas	ECHELON-2: A comparison of brentuximab vedotin and CHP with standard-of-care CHOP in the treatment of patients with CD30^+^ mature T-cell lymphomas	3	Active, not recruiting	BV, CHP, 83%/68%; CHOP, 72%/56%	NCT01777152	^87^
*Anti-CD52 antibody*							
*Alemtuzumab*	*A humanized monoclonal antibody targeting CD52*						
Alemtuzumab	Relapsed or refractory CLL	Phase 2 trial of slemtuzumab in patients with relapsed or refractory B-cell chronic lymphocytic leukemia exposed to alkylating agents and having failed fludarabine therapy	2	Completed	33%/2%	NA	^91^
Alemtuzumab vs. chlorambucil	CLL	A phase 3 study to evaluate the efficacy and safety of frontline therapy with alemtuzumab vs. chlorambucil in patients with progressive B-cell chronic lymphocytic leukemia	3	Completed	Alemtuzumab, 83%/24%; chlorambucil, 55%/2%	NA	^92^
Alemtuzumab	Advanced MF/SS	Phase 2 study of alemtuzumab in patients with advanced mycosis fungoides/Sézary syndrome	2	Completed	55%/32%	NA	^93^
Alemtuzumab	Relapsed or refractory PTCL	A pilot study of alemtuzumab therapy for patients with relapsed or chemotherapy-refractory peripheral T-cell lymphoma	2	Completed	36%/21%	NA	^94^
Alemtuzumab, FC vs. FCR	CLL	Fludarabine, cyclophosphamide, and rituximab or alemtuzumab in treating CLL	3	Completed	FCCam, 90%/19.2%; FCR, 91%/33.75%	NCT00564512	^95^
Subcutaneous alemtuzumab, bendamustine	Relapsed or refractory CLL	Bendamustine and subcutaneous alemtuzumab in relapsed or refractory chronic lymphocytic leukemia patients	1/2	Completed	68%/24%	NA	^96^
Alemtuzumab, rituximab, pentostatin	Relapsed or refractory CLL/SLL	Pentostatin, alemtuzumab, and rituximab in treating patients with relapsed or refractory chronic lymphocytic leukemia or small lymphocytic lymphoma	2	Completed	56%/28%	NCT00669318	^97^
Alemtuzumab, CHOP	PTCL	A phase 2 study of alemtuzumab plus CHOP as frontline chemotherapy for patients with peripheral T-cell lymphoma	2	Completed	80%/65%	NA	^98^
Alemtuzumab, CHOP	PTCL	GITIL trial of alemtuzumab and CHOP chemotherapy as first-line treatment of peripheral T-cell lymphoma	2	Completed	75%/71%	NA	^99^
Alemtuzumab, CHOP	PTCL	Alemtuzumab, MabCampath® with 2-weekly CHOP chemotherapy for mature T-cell non-Hodgkin’s lymphoma	2	Completed	90%/60%	NA	^100^
Alemtuzumab, CHOP14 vs.CHOP14	PTCL	Alemtuzumab and CHOP in T-cell Lymphoma	3	Completed	ALZ-CHOP, NA/52%; CHOP, NA/42%	NCT00646854	^101^
alemtuzumab, CHOP14 vs. CHOP14	PTCL	Immunotherapy in peripheral T-cell lymphoma—the role of alemtuzumab in addition to dose dense CHOP	3	Unknown	ALZ-CHOP, NA/60%; CHOP, NA/43%	NCT00725231	^102^
*Anti-CD79 antibody*							
*polatuzumab vedotin*	*An anti-CD79b monoclonal antibody conjugated to MMAE*						
Polatuzumab vedotin, rituximab	Relapsed or refractory B-NHLs/CLL	A study of escalating doses of polatuzumab vedotin in participants with relapsed or refractory B-cell non-Hodgkin’s lymphoma and chronic lymphocytic leukemia and polatuzumab vedotin in combination with rituximab in participants with relapsed or refractory B-cell non-Hodgkin’s lymphoma	1	Completed	single-agent polatuzumab vedotin: DLBCL, 56%/16%; iNHLs, 47%/20%; MCL, 100%/0%; CLL, 0%/0%; R-pola: 78%/22%	NCT01290549	^106^
Pinatuzumab vedotin, obinutuzumab, polatuzumab vedotin, rituximab	Relapsed or refractory DLBCL/FL	A study of pinatuzumab vedotin combined with rituximab or polatuzumab vedotin combined with rituximab or obinutuzumab in participants with relapsed or refractory B-cell non-Hodgkin’s lymphoma	1/2	Completed	DLBCL: R-pina, 60%/26%; R-pola, 54%/21%; FL: R-pina, 60%/5%; R-pola, 70%/45%	NCT01691898	^107^
Polatuzumab vedotin, rituximab vs. bendamustine, obinutuzumab	Relapsed or refractory DLBCL/FL	A study of polatuzumab vedotin in combination with rituximab or obinutuzumab plus bendamustine in participants with relapsed or refractory follicular or diffuse large B-cell lymphoma	1/2	Active, not recruiting	–	NCT02257567	^108^
Polatuzumab vedotin, R-CHP vs. R-CHOP	DLBCL	A study comparing the efficacy and safety of polatuzumab vedotin with rituximab-cyclophosphamide, doxorubicin, and prednisone versus rituximab-cyclophosphamide, doxorubicin, vincristine, and prednisone in participants with diffuse large B-cell lymphoma	3	Recruiting	–	NCT03274492	–
*Anti-CD19 antibody*							
*Inebilizumab*	*A CD19-targeted humanized monoclonal antibody*						
Inebilizumab	Relapsed or refractory advanced B-NHLs	A phase 1, dose-escalation study of inebilizumab in japanese adult patients with relapsed or refractory advanced B-cell malignancies	1	Completed	FL, 82%/55%; DLBCL, 50%/17%	NCT01957579	^112^
Inebilizumab, rituximab	Relapsed or refractory B-NHLs	A clinical study using inebilizumab in adult subjects with relapsed or refractory advanced B-cell malignancies	1/2	Completed	NA	NCT00983619	–
Inebilizumab, bendamustine vs. rituximab, bendamustine	Relapsed or refractory CLL	A phase 2, multicenter, open-label study of inebilizumab in adults with relapsed or refractory chronic lymphocytic leukemia	2	Completed	rituximab, bendamustine 59.7%/6.5%; inebilizumab 2mg/kg, bendamustine 52.8%/5.6%; inebilizumab 4mg/kg, bendamustine 63.9%/11.5%	NCT01466153	–
Inebilizumab, ICE/DHAP vs. rituximab, ICE/DHAP	Relapsed or refractory DLBCL	A phase 2, multicenter, randomized, open-label study of inebilizumab in adults with relapsed or refractory diffuse large B-cell lymphoma	2	Completed	inebilizumab 2mg/kg, ICE/DHAP, 46.2%/NA; inebilizumab 4mg/kg, ICE/DHAP, 43.6%/NA; rituximab, ICE/DHAP, 47.5%/NA	NCT01453205	–
*Tafasitamab*	*A novel Fc-engineered, humanized, anti-CD19 antibody with enhanced ADCC*						
Tafasitamab	Relapsed or refractory NHLs	Study of Fc-optimized anti-CD19 antibody tafasitamab to treat non-Hodgkin’s lymphoma	2	Active, not recruiting	DLBCL, 26%/6%; FL, 29%/9%; iNHLs, 27%/18%	NCT01685008	^114^
Tafasitamab, lenalidomide	Relapsed or refractory DLBCL	A study to evaluate the safety and efficacy of lenalidomide with tafasitamab in patients with relapsed or refractory DLBCL	2	Active, not recruiting	58%/33%	NCT02399085	^115^
Tafasitamab, lenalidomide	CLL/SLL, PLL	Phase 2 tafasitamab in combination with lenalidomide for patients with relapsed or refractory CLL/SLL or PLL or older patients with untreated CLL/SLL or PLL	2	Active, not recruiting	–	NCT02005289	–
Tafasitamab, bendamustine vs. rituximab, bendamustine	Relapsed or refractory DLBCL	A trial to evaluate the efficacy and safety of tafasitamab with bendamustine versus rituximab with bendamustine in adult patients with relapsed or refractory diffuse large B-cell lymphoma	2/3	Recruiting	–	NCT02763319	–
*Coltuximab ravtansine*	*A CD19-targeted ADC consists of CD19 antibody and a cytotoxic maytansinoid, DM4, which is a potent inhibitor of tubulin polymerization and microtubule assembly*						
Coltuximab ravtansine	Relapsed or refractory DLBCL	Coltuximab ravtansine as single agent in relapsed or refractory diffuse large B-cell lymphoma patients	2	Completed	43.9%/14.6%	NCT01472887	^116^
*loncastuximab tesirine*	*An ADC consisting of an anti-CD19 humanized monoclonal antibody conjugated to a cytotoxic, crosslinking agent pyrrolobenzodiazepine dimer*						
loncastuximab tesirine	Relapsed or refractory DLBCL	Study to evaluate the efficacy and safety of ioncastuximab tesirine in patients with relapsed or refractory diffuse large B-cell lymphoma	2	Active, not recruiting	–	NCT03589469	–
loncastuximab tesirine	Relapsed or refractory B-NHLs	Study of ioncastuximab tesirine in patients with relapsed or refractory B-cell lineage non-Hodgkin’s lymphoma	1	Completed	NA	NCT02669017	–
loncastuximab tesirine, ibrutinib	DLBCL/MCL	Safety and antitumor activity study of loncastuximab tesirine plus ibrutinib in diffuse large B-cell or mantle cell lymphoma	1	Recruiting	–	NCT03684694	–
loncastuximab tesirine, durvalumab	DLBCL/MCL/FL	Safety and antitumor activity study of loncastuximab tesirine and durvalumab in diffuse large B-cell, mantle cell, or follicular lymphoma	1	Recruiting	–	NCT03685344	–
*Anti-CD37 antibody*							
*Otlertuzumab*	*A humanized variant of SMIP-016 built on the ADAPTIR platform*						
Otlertuzumab	Relapsed or refractory NHL/CLL	Phase 1/1b study of otlertuzumab in patients with previously treated CLL or select subtypes of non-Hodgkin’s lymphoma	1	Completed	FL, 12.5%/0%; MCL, 0%/0%; WM, 25%/0%; CLL, 23%/0%	NCT00614042	^123,124^
Otlertuzumab, bendamustine vs. bendamustine	Relapsed CLL	Safety and efficacy study of otlertuzumab plus bendamustine vs. bendamustine in relapsed chronic lymphocytic leukemia	1/2	Completed	Otlertuzumab and bendamustin, 69%/9%; bendamustin, 39%/3%	NCT01188681	^125^
Otlertuzumab, bendamustine, rituximab	Relapsed iNHLs	A study of otlertuzumab in combination with rituximab and bendamustine in subjects with relapsed indolent lymphoma	1	Completed	83%/32%	NCT01317901	^126^
*IMGN529*	*Consisting of an anti-CD37 antibody coupled with the maytansine-derived anti-microtubule agent, DM1*						
IMGN529	Relapsed or refractory NHLs/CLL	IMGN529 in treating patients with relapsed or refractory non-Hodgkin’s lymphoma and chronic lymphocytic leukemia	1	Completed	DLBCL, 22.2%/5.6%; FL, 7.7%/0%; MCL, 0%/0%; MZL, 0%/0%	NCT01534715	^129^
*AGS67E*	*A fully human monoclonal IgG2 antibody conjugated via a protease-cleavable linker to MMAE*						
AGS67E	Relapsed or refractory lymphoid malignancy	A study to evaluate safety, tolerability, and pharmacokinetics of escalating doses of AGS67E given as monotherapy in subjects with refractory or relapsed lymphoid malignancies	1	Active, not recruiting	–	NCT02175433	–
*Betalutin*	*A novel ARC targeting the CD37 antigen*						
Betalutin	Relapsed or refractory NHLs	A Phase 1/2 study of betalutin for treatment of relapsed non-Hodgkin’s lymphoma	1/2	Recruiting	–	NCT01796171	–
Betalutin	Relapsed or refractory DLBCL	Study of betalutin for treatment of relapsed or refractory non-Hodgkin’s lymphoma (LYMRIT-37-05)	1	Recruiting	–	NCT02658968	–
Betalutin, rituximab	Relapsed or refractory FL	Study of safety and efficacy of betalutin and rituximab in patients with FL	1	Recruiting	–	NCT03806179	–
*Anti-CCR4*							
*Mogamulizumab*	*A defucosylated humanized monoclonal antibody directed against CCR4*						
Mogamulizumab	ATLL	Phase 2 study of KW-0761 in subjects with CCR4^+^ adult T-cell leukemia/lymphoma	2	Completed	50%/31%	NCT00920790	^138^
Mogamulizumab, mLSG15 vs. mLSG15	ATLL	Multicenter, randomized, open-label, parallel-group study to compare mLSG15 plus mogamulizumab to mLSG15	2	Completed	Mogamulizumab, mLSG15, 86%/52%; mLSG15, 75%/33%	NCT01173887	^139^
Mogamulizumab	PTCL	Safety study to evaluate monoclonal antibody mogamulizumab in subjects with peripheral T-cell lymphoma	1/2	Completed	36.8%/7.9%	NCT00888927	^140^
Mogamulizumab	PTCL	Study of mogamulizumab in subjects with CCR4^+^ T-cell lymphoma	2	Completed	35%/14%	NCT01192984	^141^
Mogamulizumab vs. vorinostat	Relapsed or refractory CTCL	Study of mogamulizumab versus vorinostat in relapsed or refractory CTCL	3	Active, not recruiting	Mogamulizumab, 28%/3%; vorinostat, 5%/0%	NCT01728805	^142^
*Anti-CD25 antibody*							
^*90*^*Y-daclizumab*	*A radiolabeled anti-CD25 antibody*						
^90^Y-daclizumab	HL/NHLs	^90^Y-Daclizumab to treat Hodgkin’s disease, non-Hodgkin’s lymphoma and lymphoid leukemia	1/2	Completed	Relapsed HL, 50%/30%	NCT00001575	^145^
^*90*^*Y-basiliximab*	*A radiolabeled anti-CD25 antibody*						
^90^Y-basiliximab, BEAM	Relapsed or refractory HL	Radiolabeled monoclonal antibody therapy and combination chemotherapy before stem cell transplant in treating patients with primary refractory or relapsed Hodgkin’s lymphoma	1	Active, not recruiting	–	NCT01476839	–
^90^Y-basiliximab, BEAM	Mature T-NHLs	90Y-basiliximab and combination chemotherapy before stem cell transplant in treating patients with mature T-cell non-Hodgkin’s lymphoma	1	Recruiting	–	NCT02342782	–
*Camidanlumab tesitine*	*A CD25 antibody-drug conjugate*						
Camidanlumab tesitine	Relapsed or refractory HL/NHLs	Study of camidanlumab tesitine in patients with relapsed or refractory Hodgkin’s and non-Hodgkin’s lymphoma	1	Completed	NA	NCT02432235	–
*Anti-CD38 antibody*							
*Daratumumab*	*An anti-CD38 monoclonal antibody*						
Daratumumab	Relapsed or refractory NKTCL, nasal type	A study to assess the clinical efficacy and safety of daratumumab in participants with relapsed or refractory NK/T-cell lymphoma, nasal type	2	Active, not recruiting	35.7%/0%	NCT02927925	^149^
*Anti-CD40 antibody*							
*Dacetuzumab*	*A humanized IgG1 monoclonal antibody targeting CD40*						
Dacetuzumab	NHL	A safety study of dacetuzumab in patients with non-Hodgkin’s lymphoma	1	Completed	12%/2%	NCT00103779	^152^
Dacetuzumab	Relapsed DLBCL	Study of dacetuzumab in patients with relapsed diffuse large B-cell lymphoma	2	Completed	9%/4%	NCT00435916	^153^
Dacetuzumab, R-ICE vs. placebo, R-ICE	Relapsed DLBCL	A randomized phase 2 placebo-controlled study of R-ICE chemotherapy with and without dacetuzumab for patients with DLBCL	2	Terminated	Dacetuzumab, R-ICE, 66%/33%; placebo, R-ICE, 64%/36%	NCT00529503	^154^
*Anti-CD74 antibody*							
*milatuzumab*	*A humanized antibody against CD74*						
Milatuzumab, veltuzumab	Relapsed or refractory B-NHLs	Veltuzumab and milatuzumab in treating patients with relapsed or refractory B-cell non-Hodgkin’s lymphoma	1/2	Completed	FL, 33%/7%; DLBCL, 0%/0%; MCL, 17%/0%; MZL, 100%/50%; WM, 0%/0%	NCT00989586	^156^
*Anti-CD80 antibody*							
*Galiximab*	*An anti-CD80 monoclonal antibody*						
Galiximab	Relapsed or refractory HL	Galiximab in treating patients with relapsed or refractory Hodgkin’s lymphoma	2	Completed	10.3%/NA	NCT00516217	–
Galiximab	Relapsed or refractory FL	Phase 1/2 study of galiximab for relapsed or refractory follicular lymphoma	1/2	Completed	11%/6%	–	^159^
Galiximab, rituximab	Relapsed or refractory FL	Safety and efficacy of galiximab in combination with rituxan in the treatment of non-Hodgkin’s lymphoma	1/2	Completed	66%/19%	NCT00048555	^160^
*Anti-CD158k antibody*							
*IPH4102*	*An anti-CD158k monoclonal antibody*						
IPH4102	Relapsed or refractory CTCL	Study of IPH4102 in patients with relapsed or refractory cutaneous T-cell lymphoma	1	Active, not recruiting	45%/0%	NCT02593045	^165^
IPH4102 vs. IPH4102, gemcitabine, oxaliplatin	Advanced T-NHLs	IPH4102 alone or in combination with chemotherapy in patients with advanced T-cell lymphoma	2	Recruiting	–	NCT03902184	–
*Bispecific T cell Engager*							
*Blinatumomab*	*A CD19/CD3 Bispecific T cell Engager*						
Blinatumomab	Relapsed NHLs	Safety study of the bispecific T-cell engager blinatumomab in patients with relapsed NHLs	1	Completed	DLBCL, 55%/36%; MCL, 71%/43%; FL, 80%/40%	NCT00274742	^169^
Blinatumomab	Relapsed or refractory DLBCL	Clinical study with blinatumomab in patients with relapsed or refractory diffuse large B-cell lymphoma	2	Completed	43%/19%	NCT01741792	^170^
Blinatumomab	Relapsed or refractory aggressive B-NHLs	Study to evaluate safety and efficacy of blinatumomab in subjects with relapsed or refractory aggressive B-cell NHL	2	Active, not recruiting	–	NCT02910063	–
*Mosunetuzumab*	*A CD20/CD3 Bispecific T cell Engager*						
Mosunetuzumab	DLBCL	A trial of mosunetuzumab as consolidation therapy in participants with diffuse large B-cell lymphoma following first-line immunochemotherapy and as therapy in participants with previously untreated diffuse large B-cell lymphoma who are unable to tolerate full-dose chemotherapy	1/2	Recruiting	–	NCT03677154	–
Mosunetuzumab, polatuzumab vedotin	B-NHLs	A study to evaluate the safety and efficacy of mosunetuzumab in combination with polatuzumab vedotin in B-cell non-Hodgkin’s lymphoma	1	Recruiting	–	NCT03671018	–
Mosunetuzumab, polatuzumab vedotin, CHP vs.mosunetuzumab, CHOP	B-NHLs	A phase 1/2 study investigating the safety, tolerability, pharmacokinetics, and efficacy of mosunetuzumab in combination With CHOP or CHP-polatuzumab vedotin in participants With B-cell non-Hodgkin’s lymphoma	1/2	Recruiting	–	NCT03677141	–
*RO7082859*	*A CD20/CD3 Bispecific T cell Engager*						
RO7082859, obinutuzumab	Relapsed or refractory B-NHLs	A dose escalation study of RO7082859 as a single agent and in combination with obinutuzumab, administered after a fixed, single pre-treatment dose of obinutuzumab in participants with relapsed or refractory B-cell non-Hodgkin’s lymphoma	1	Recruiting	–	NCT03075696	–
RO7082859, atezolizumab, obinutuzumab	Relapsed or refractory B-NHLs	An open-label phase 1b study of RO7082859 and atezolizumab in adult patients with relapsed or refractory B-cell non-Hodgkin’s lymphoma	1	Recruiting	–	NCT03533283	–
RO7082859, obinutuzumab/rituximab, CHOP	B-NHLs	A study of RO7082859 in combination with rituximab or obinutuzumab plus cyclophosphamide, doxorubicin, vincristine, and prednisone in participants with non-Hodgkin’s lymphomas	1	Recruiting	–	NCT03467373	–
*REGN1979*	*A CD20/CD3 Bispecific T cell Engager*						
REGN1979	Relapsed or refractory FL	Assess the antitumor activity and safety of REGN1979 in patients with relapsed or refractory follicular lymphoma	2	Recruiting	–	NCT03888105	–
REGN1979	B-NHLs	A phase 1 study to investigate the safety and tolerability of REGN1979 in patients With CD20^+^ B-cell malignancies	1	Recruiting	–	NCT02290951	–
REGN1979, REGN2810	B-NHLs	Study of REGN2810 and REGN1979 in patients with lymphoma	1	Recruiting	–	NCT02651662	–
*XmAb13676*	*A CD20/CD3 Bispecific T cell Engager*						
XmAb13676	B-NHLs, CLL/SLL	Study to evaluate safety and tolerability of XmAb13676 in patients with CD20^−^ expressing hematologic malignancies	1	Recruiting	–	NCT02924402	–

NA: ORR or CR are not available on the clinicaltrials.gov or from the published article although the trial has been completed

*iNHLs* indolent NHLs, *CHOP* cyclophosphamide, doxorubicin, vincristine, prednisolone, *CVP* cyclophosphamide, vincristine, and prednisolone, *FC* fludarabine and cyclophosphamide, *G-CHOP* obinutuzumab, cyclophosphamide, doxorubicin, vincristine and prednisone, *G-FC* obinutuzumab, fludarabine and cyclophosphamide, *G-Clb* obinutuzumab and chlorambucil, *R-Clb* rituximab and chlorambucil, *BEAM* carmustine, etoposide, cytarabine, melphalan chemotherapy, *R-CHOP* rituximab, cyclophosphamide, doxorubicin, vincristine, prednisolone, *R-InO* rituximab and inotuzumab ozogamicin, *RB* rituximab and bendamustine, *RG* rituximab and gemcitabine, *R-G-CVP* rituximab, gemcitabine, cyclophosphamide, vincristine and prednisolone, *GVD* gemcitabine, vinorelbine, and liposomal doxorubicin, *BV* brentuximab vedotin, *A+AVD* brentuximab vedotin, doxorubicin, vinblastine, and dacarbazine, *ABVD* doxorubicin, bleomycin, vinblastine, and dacarbazine, *CHP* cyclophosphamide, doxorubicin and prednisone, *FCCam* fludarabine cyclophosphamide and alemtuzumab, *FCR* fludarabine cyclophosphamide and rituximab, *CHOP14* cyclophosphamide, doxorubicin, vincristine, and prednisone every 14 days, *ALZ-CHOP* alemtuzumab, cyclophosphamide, doxorubicin, vincristine and prednisone, *R-pola* rituximab and polatuzumab vedotin, *R-pina* rituximab and pinatuzumab vedotin, *R-CHP* rituximab, cyclophosphamide, doxorubicin and prednisone, *ICE* ifosfamide, carboplatin, etoposide, *DHAP* dexamethasone, high-dose cytarabine, cisplatin, *PLL* prolymphocytic leukemia, *mLSG15* a dose-intensified chemotherapy

Obinutuzumab (GA101, Gazyva™) is a humanized type II mAb that can induce ADCC and direct apoptosis both in vitro and in vivo.^17,18^ In a phase 1/2 study (NCT00517530), obinutuzumab as monotherapy showed clinical activity with an acceptable safety profile in aggressive B-NHLs.^19^ Moreover, clinical trials (NCT01059630, NCT01332968, and NCT00825149) of obinutuzumab in combination with other chemotherapy regimens showed promising results in relapsed or refractory indolent B-NHLs^20,21^ and untreated follicular lymphoma (FL).^22^ The most common nonhematologic AEs were grade 1-2 infusion-related reactions, and the most common hematologic AE was neutropenia. For CLL, the findings of a phase 3 study (NCT01010061) of naïve elderly patients suggested that obinutuzumab in combination with chlorambucil yields better response rates and longer progression-free survival (PFS) than rituximab with chlorambucil and chlorambucil; thus, obinutuzumab became the first drug with “breakthrough therapy designation” approved by the FDA for the treatment of untreated CLL in combination with chlorambucil.^23^ Recently, a multicenter, randomized, phase 3 trial (iLLUMINATE, NCT02264574) demonstrated the advantages of obinutuzumab plus ibrutinib over obinutuzumab plus chlorambucil as a first-line treatment for CLL.^24^

Ublituximab is another type I, chimeric, recombinant IgG1 mAb targeting a unique epitope on the CD20 antigen, glycoengineered to enhance affinity for all FcRIIIa variants, leading to greater ADCC than other anti-CD20 mAbs such as rituximab and ofatumumab.^25^ Ublituximab demonstrated efficacy and safety as a single agent in early clinical trials in patients with B-NHLs and CLL,^25,26^ and it was further investigated in combination regimens. A phase 2 study (NCT02013128) combining ublituximab with ibrutinib was carried out in relapsed or refractory CLL and obtained an overall response rate (ORR) of 88%. Of note, in high-risk patients bearing del17p, del11q, or *TP53* mutations, the ORR was 95%.^27^ A phase 3 trial (GENUINE, NCT02301156) of ublituximab plus ibrutinib in high-risk relapsed or refractory CLL reported an ORR of 78% for the combination arm vs 45% for the monotherapy arm.^28^ The combination of ublituximab and umbralisib with/without ibrutinib had indicated tolerability and activity in patients with relapsed or refractory B-NHLs and CLL in a phase 1 study (NCT02006485).^29,30^

Other humanized type I anti-CD20 mAbs, such as veltuzumab (IMMU-106) and ocrelizumab (PRO70769), also showed efficacy in patients with relapsed or refractory B-NHLs and FL in phase 1/2 studies (NCT00285428 and NCT02723071).^31,32^ In addition, progress has been made in the study of biosimilars of rituximab. CT-P10 (CELLTRION) was the first mAb biosimilar anticancer drug to gain international regulatory approval following the results of phase 3 trials (NCT02260804 and NCT02162771) in FL.^33,34^ Other examples of rituximab biosimilars include GP2013, PF-05280586, and ABP798. GP2013 has also been approved in the European Union for its efficacy data from a phase 3 trial in FL (ASSIST-FL, NCT01419665).^35^ The phase 3 study (NCT02213263) of PF-05280586 displayed positive results as well.^36^ Moreover, ABP798 is currently under study (NCT02747043).

Radioimmunotherapy (RIT) has also emerged as an important therapeutic strategy for B-NHLs. Ibritumomab tiuxetan (IDEC-Y2B8, Zevalin®) is a radiolabeled anti-CD20 mAb that targets the same epitope on the CD20 molecule as rituximab. This compound chelates the radioactive particle yttrium-90 (^90^Y), which delivers high beta energy to improve its ability to kill bulky, poorly vascularized tumors.^37^ Ibritumomab tiuxetan is effective in both rituximab-naïve and rituximab-resistant FL, as well as in transformed B-NHLs.^38,39^ Consequently, ibritumomab tiuxetan acquired FDA approval for rituximab-naïve relapsed or refractory low-grade B-NHLs and transformed NHLs. The long-term toxicity of developing myelodysplastic syndrome and acute myelogenous leukemia was observed.^40^ Furthermore, ibritumomab tiuxetan has shown promising results in the first-line treatment of untreated FL (NCT00772655 and NCT01493479).^41,42^ In addition, a phase 3 trial (FIT, NCT00185393) observed an improvement of efficacy through ibritumomab tiuxetan consolidation;^43,44^ thus, the FDA approved this agent for consolidation therapy in untreated FL patients who achieve partial response (PR) or complete response (CR) after first-line chemotherapy. A phase 3 study of rituximab with or without ibritumomab tiuxetan in untreated FL is ongoing (NCT02320292). Ibritumomab tiuxetan is also being evaluated as consolidation therapy in relapsed or refractory FL in a phase 3 study (NCT01827605). Additionally, ibritumomab tiuxetan combined with high-dose chemotherapy prior to autologous stem cell transplantation (ASCT) has also been proven to be safe with relative efficacy.^45,46^

### CD22

CD22 is a single-spanning membrane glycoprotein with a molecular weight of 140,000 located on the surface of B-cells. It is mostly expressed in mature B-cells and many malignant B-cells.^47,48^ CD22 acts as a negative regulator of B-cell receptor (BCR)-induced signaling and plays a critical role in B-cell activation.^47,49^ The inhibitory function of CD22 and its restricted expression on B-cells make CD22 an ideal target in NHLs.

Epratuzumab is a humanized IgG1 mAb targeting CD22. The crosslinking of CD22 by epratuzumab triggers BCR signaling and caspase-dependent apoptosis in human lymphoma cells.^50^ Preclinical studies demonstrated that CD22 mAbs had independent lymphomacidal properties.^51^ Single-agent epratuzumab has been investigated in both indolent and aggressive NHLs. In an early phase 1/2 trial including 55 patients with recurrent NHLs, epratuzumab showed a response in FL (ORR 24%), while no response was observed in other indolent lymphomas.^52^ In another concurrent phase 1/2 trial, 15% of patients with relapsed or refractory diffuse large B-cell lymphoma (DLBCL) responded to epratuzumab.^53^ The combination of epratuzumab with rituximab has been tested in a multicenter phase 2 trial and exhibited an ORR of 54% in FL and 57% in small lymphocytic lymphoma (SLL).^54^ Epratuzumab plus rituximab was also studied in untreated FL and obtained an ORR of 88.2% (NCT00553501).^55^ In aggressive lymphomas, a phase 2 trial (NCT00301821) showed that epratuzumab combined with rituximab, cyclophosphamide, doxorubicin, vincristine, and prednisolone (R-CHOP) achieved an ORR of 96% in DLBCL, with 3-year event-free survival (EFS) and OS rates of 70% and 80%, respectively.^56^

Conjugate antibodies utilize the direct conjugation of mAbs with cytotoxic agents, and there are two types of antibody-based conjugates: antibody-drug conjugates (ADCs) and immunotoxins.^57^ ADCs are mAbs connected to bioactive drugs by chemical linkers. Inotuzumab ozogamicin (InO, CMC-544) is a CD22-targeted ADC combining a humanized IgG4 anti-CD22 mAb with calicheamicin, an enediyne antibiotic, which causes DNA damage and cell apoptosis.^58,59^ The combination of InO with rituximab in a phase 1/2 study (NCT00299494) of relapsed FL, DLBCL, and refractory aggressive NHL induced ORRs of 87%, 74%, and 20%, respectively. The most common grade 3–4 AEs were thrombocytopenia (31%) and neutropenia (22%).^60^ However, InO plus rituximab failed to obtain positive results in a randomized phase 3 trial (NCT01232556) of relapsed or refractory CD22^+^ aggressive B-NHLs and FLs.^61^ A phase 2 trial (NCT01679119) of InO plus rituximab, cyclophosphamide, vincristine, and prednisolone (R-CVP) in chemotherapy-naïve DLBCL not suitable for anthracycline-based treatment is ongoing. An immunotoxin is a genetically engineered protein consisting of a targeting portion linked to a toxin. Moxetumomab pasudotox connects anti-CD22 to PE38, a fragment of Pseudomonas exotoxin A, and induces apoptosis through the inhibition of protein synthesis.^62,63^ A phase 1 study (NCT00462189) demonstrated an ORR of 86% in hairy cell leukemia (HCL) patients with no dose-limiting toxicity.^64^ Moreover, a pivotal phase 3 study (NCT01829711) for relapsed or refractory HCL obtained an ORR of 75%, with a CR rate of 41%.^65^ The FDA approved moxetumomab pasudotox (Lumoxiti) for the treatment of adult patients with relapsed or refractory HCL.

### CD30

CD30 is a 120-kDa type I transmembrane receptor of the tumor necrosis factor receptor (TNFR) superfamily.^66^ The binding of CD30 with its ligand induces signal transduction through several downstream pathways, especially nuclear factor-κB (NF-κB).^67^ CD30 is normally expressed on activated B cells, T cells, and NK cells, as well as virally infected lymphocytes. In addition, CD30 is universally expressed in HL and anaplastic large cell lymphoma (ALCL).^68,69^ Other lymphoproliferative disorders, such as DLBCL, primary mediastinal B-cell lymphoma (PMBCL), peripheral T-cell lymphoma (PTCL), mycosis fungoides (MF), Sézary syndrome (SS) and adult T-cell leukemia/lymphoma (ATLL), can also express CD30 to various degrees.^70–72^

A chimeric mAb SGN-30, consisting of the variable region of an anti-CD30 murine mAb with human gamma 1 heavy chain and kappa light chain constant regions, promotes growth arrest and DNA fragmentation in vitro and exhibits antitumor activity in HL models.^73^ In a phase 2 study of relapsed or refractory HL or ALCL, SGN-30 showed only a modest effect in ALCL (2 CR and 5 PR in 41 ALCL patients).^74^ However, another phase 2 trial used a combination of SGN-30 with gemcitabine, vinorelbine, and liposomal doxorubicin in relapsed HL and showed an ORR of 65%, while grades 3–5 pneumonitis occurred in five patients, leading to the premature closure of the trial.^75^

Brentuximab vedotin (BV, Adcetris), a CD30 ADC, connects an anti-CD30 antibody with the anti-mitotic agent monomethyl auristatin E (MMAE) via a valine-citrulline peptide-linker. It showed strong activity against CD30^+^ tumor cell lines in vitro, as well as xenograft models of HL and ALCL.^76^ A phase 1 dose-escalation study (NCT00430846) of BV in 45 patients with relapsed or refractory CD30^+^ hematological malignancies (mainly HL) determined the optimal dose of BV as 1.8 mg/m^2^ intravenously every 3 weeks and showed an ORR of 38%.^2^ Common AEs of BV include fatigue, pyrexia, diarrhea, nausea, peripheral neuropathy, neutropenia, anemia, and arthralgias.^2^ Other AEs, such as anaphylaxis and acute pancreatitis, have also been reported.^77,78^ BV was granted FDA accelerated approval for the treatment of relapsed or refractory HL and ALCL based on the results of two phase 2 studies. NCT00848926 enrolled 102 relapsed or refractory HL patients and obtained an ORR of 75% (CR 34%) with a median duration of response (DoR) of 6.7 months.^79^ NCT00866047 showed an ORR of 86% (CR 57%) with a median DoR of 12.6 months in 58 patients with relapsed or refractory CD30^+^ ALCL.^80^ After approval, the FDA issued a boxed warning related to the risk of progressive multifocal leukoencephalopathy and added a contraindication warning for the concomitant use of BV and bleomycin due to pulmonary toxicity.

In addition to ALCL, BV has shown efficacy as a single agent in other T-NHLs (NCT01421667).^81^ In addition to systemic lymphomas, BV was also utilized in primary CD30^+^ cutaneous lymphomas and showed encouraging efficacy.^82^ A phase 3 randomized multicenter trial (ALCANZA, NCT01578499) was conducted to evaluate single-agent BV vs a control arm of the investigator’s choice of standard therapies in patients with CD30^+^ primary cutaneous ALCL or MF. ALCANZA demonstrated an improvement in ORR (ORR: 56.3% in the BV arm vs. 12.5% in the conventional therapy arm),^83^ leading to FDA approval for the treatment of adult patients with primary cutaneous ALCL or CD30^+^ MF.

For BV combined with chemotherapy, in a multicenter phase 3 trial (NCT01712490) involving patients with untreated stage III or IV HL, patients were randomized to receive BV, doxorubicin, vinblastine, and dacarbazine (A+AVD) or doxorubicin, bleomycin, vinblastine, and dacarbazine (ABVD). The results showed that at a median follow-up of 24.6 months, the 2-year modified PFS rates in the A+AVD and ABVD groups were 82.1% and 77.2%, respectively. Neutropenia and peripheral neuropathy were the most common AEs.^84^ Based on these promising clinical data, the FDA expanded the approval of BV for the first-line treatment of stage III or IV HL in combination with chemotherapy. A phase 1 study (NCT01309789) combining BV with cyclophosphamide, doxorubicin, and prednisolone in patients with CD30^+^ PTCL resulted in an objective response in all patients (CR 88%).^85^ Moreover, the five-year follow-up demonstrated durable remission in half of the patients after combination therapy.^86^ Therefore, a randomized phase 3 trial (ECHELON-2, NCT01777152) comparing BV plus cyclophosphamide, doxorubicin and prednisone (CHP) with CHOP was conducted in untreated patients and demonstrated a significant improvement in PFS and OS with a manageable safety profile when using BV plus CHP.^87^ The FDA thus approved BV in combination with chemotherapy for adults with untreated ALCL or other CD30^+^ PTCL.

### CD52

The CD52 antigen is a small glycopeptide highly expressed on normal and malignant B and T lymphocytes. The exact function of CD52 remains undefined, but in vitro studies have proven that it is a costimulatory molecule for the induction of CD4^+^ regulatory T-cells.^88^

Alemtuzumab (Campath®) is a humanized mAb targeting CD52 that can induce complement-mediated lysis as well as caspase-independent cell death in malignant lymphoid cells.^89,90^ Single-agent alemtuzumab received accelerated approval by the FDA for CLL patients who had received alkylating agents and failed fludarabine therapy.^91^ A phase 3 randomized trial comparing alemtuzumab to chlorambucil as first-line treatment showed significantly improved PFS, time to alternative treatment, ORR and CR, with manageable toxicity in CLL.^92^ Alemtuzumab has also been evaluated as monotherapy in T-NHLs and exhibited efficacy in advanced MF, Sézary syndrome (SS), and relapsed or refractory PTCL,^93,94^ where hematological toxicity and cytomegalovirus (CMV) reactivation were the most common AEs.

Alemtuzumab-containing chemoimmunotherapy regimens can be effective but have been limited by their toxicities in CLL (NCT00564512).^95^ The bendamustine and subcutaneous alemtuzumab combination was proven to be as effective as the combination of fludarabine, cyclophosphamide, and cladribine and was safe in heavily pretreated and elderly patients.^96^ Other attempts at combining pentostatin, alemtuzumab, and low-dose rituximab (NCT00669318) also yielded efficacy and tolerability in relapsed or refractory 17p13-deleted CLL.^97^ The combination of alemtuzumab and CHOP-based chemotherapy was explored in untreated PTCL.^98–100^ Phase 3 randomized studies (NCT00646854 and NCT00725231) of alemtuzumab plus CHOP in either young or elderly PTCL patients achieved improved PFS or OS.^101,102^

### CD79

CD79, composed of CD79A and CD79B components, is a main BCR signaling component and is expressed almost exclusively on B-cells and B-NHLs. CD79 expression precedes immunoglobulin heavy-chain gene rearrangement and CD20 expression during B-cell development but disappears in the late stage of B-cell differentiation.^103^ When BCR is cross-linked, CD79 is targeted to a lysosome-like compartment^104^ and induces cell apoptosis or triggers cell activation and division with rescue signals from T cells.^105^ Therefore, CD79 has become an attractive target for the use of ADCs, and preclinical studies found two stable-linker ADCs capable of killing NHL cell lines in vitro and in xenograft models.^106^

Polatuzumab vedotin (DCDS4501A) is an anti-CD79B mAb conjugated to MMAE. In a phase 1 study (NCT01290549) in relapsed or refractory B-NHLs and CLL, no objective response was observed in CLL, while at the recommended phase 2 dose of 2.4 mg/kg, objective responses were obtained in 23 of 42 patients with NHLs by polatuzumab vedotin monotherapy (56% in patients with DLBCL, 47% with indolent NHLs, and 100% with mantle cell lymphoma (MCL)) and in 7 of 9 patients by polatuzumab vedotin plus rituximab.^106^ Polatuzumab vedotin was further evaluated in a phase 2 trial (NCT01691898) in combination with rituximab in patients with relapsed or refractory NHLs. The results showed that the ORRs and CR rates were 54% and 21% in DLBCL and 70% and 45% in FL, respectively. Grade ≥3 AEs occurred in 77% of DLBCL patients and 50% of FL patients, mainly as neutropenia, anemia, and diarrhea.^107^ Furthermore, the findings of a phase 2 study (NCT02257567) pointed out that adding polatuzumab vedotin to bendamustine and rituximab (BR) treatment improved survival in patients with relapsed or refractory DLBCL.^108^ The combination of polatuzumab vedotin with rituximab, cyclophosphamide, doxorubicin and prednisone (R-CHP) vs R-CHOP in DLBCL is currently being investigated in a phase 3 study (POLARIX, NCT03274492).

### CD19

CD19 is a B-cell-specific member of the immunoglobulin superfamily that augments signals by the pre-BCR/BCR and modulates B-cell fate decisions at multiple stages of development.^109^ CD19 is highly expressed in nearly all B-NHLs, making it an excellent target for immune-based therapies.^110^

Inebilizumab (MEDI-551) is a CD19-targeted humanized mAb that has potent ADCC activity in vitro and in vivo in preclinical studies.^111^ Inebilizumab monotherapy has been evaluated in phase 1 studies and showed acceptable toxicity and promising efficacy in patients with relapsed or refractory FL and DLBCL (NCT01957579).^112^ A phase 1/2 trial (NCT00983619) of inebilizumab alone and in combination with rituximab in FL, CLL, and DLBCL has recently been completed. Regarding inebilizumab in combination with chemotherapy, recent clinical trials did not yield promising results. A phase 2 trial (NCT01466153) comparing inebilizumab plus bendamustine and BR did not find any significant difference in the ORR between the two groups. Another randomized phase 2 study (NCT01453205) on rituximab plus ifosfamide, carboplatin, and etoposide (ICE)/dexamethasone, high-dose cytarabine, and cisplatin (DHAP) vs inebilizumab plus ICE/DHAP in patients with relapsed or refractory DLBCL did not show any significant difference in ORR, PFS, or OS.

Tafasitamab (MOR208, XmAb®5574) is a novel Fc-engineered, humanized, anti-CD19 antibody with enhanced ADCC, antibody-dependent cellular phagocytosis and apoptosis, as well as more potent antitumor activity in vivo than its IgG1 analog.^113^ These effects were achieved by increasing the affinity for FcγRIIIa on effector cells through the introduction of S239D and I332E amino acid substitutions to the Fc domain. Tafasitamab monotherapy exhibited promising clinical activity in patients with relapsed or refractory B-NHLs with a favorable safety profile. The ORRs were 26%, 29%, and 27% in DLBCL, FL, and other indolent NHLs, respectively, with 9% of patients experiencing grade 3–4 neutropenia (NCT01685008).^114^ Furthermore, combinations with lenalidomide and bendamustine are being evaluated in recent phase 2/3 clinical trials (NCT02399085, NCT02005289, and NCT02763319). Based on the preliminary data from a phase 2 study (L-MIND, NCT02399085) in combination with lenalidomide, this mAb was granted FDA breakthrough therapy and fast track designations for DLBCL. Eighty-one patients enrolled in the L-MIND study obtained an ORR of 58%, including 33% CR, with no unexpected toxicities observed. With a median follow-up of 12 months, the median PFS was 16.2 months.^115^

In addition, the CD19-targeted ADC coltuximab ravtansine (SAR3419) consists of a cytotoxic maytansinoid, DM4, which is a potent inhibitor of tubulin polymerization and microtubule assembly. In a phase 2 study (NCT01472887), this agent showed good tolerance and moderate clinical responses in pretreated patients with relapsed or refractory DLBCL (ORR 43.9%).^116^ A novel ADC based on coltuximab ravtansine showed promising preclinical data and may become an attractive candidate for clinical investigation.^117^

Loncastuximab tesirine (ADCT-402) is a novel CD19-targeted ADC that delivers SG3199, a highly cytotoxic pyrrolobenzodiazepine dimer, and showed highly targeted cytotoxicity in vitro and antitumor activity in vivo in preclinical studies.^118^ A pivotal phase 2 study (NCT03589469) is currently ongoing on relapsed or refractory DLBCL, as well as phase 1 studies (NCT02669017, NCT03684694, and NCT03685344) on relapsed or refractory B-NHLs.

### CD37

CD37 is a heavily glycosylated transmembrane protein of the tetraspanin superfamily and represents one of the specific proteins for normal and malignant mature B-cells. The expression of CD37 is detected in CLL, Burkitt lymphoma (BL), MCL, and FL,^119,120^ and it is involved in various biological processes, such as cell adhesion, proliferation, differentiation, intercellular communication via exosomes and immune response.^121^

Small modular immunopharmaceuticals (SMIPs) are disulfide-linked single-chain proteins comprised of one antigen-binding region (V_H_/V_L_), a hinge, and an Fc domain of the human IgG1 region (CH2-CH3). Due to their smaller size, SMIPs may have better tissue penetration than mAbs. SMIP-016 is a homodimeric protein specially engineered to exhibit the full binding activity of an anti-CD37 antibody. Preclinical studies have demonstrated that SMIP-016 can induce apoptosis and ADCC in B-cell leukemia/lymphoma cell lines and primary CLL cells.^122^

Otlertuzumab (TRU-016) is a humanized variant of SMIP-016 built on the ADAPTIR (modular protein technology) platform. In a phase 1 study (NCT00614042), otlertuzumab was well tolerated and exhibited modest activity as monotherapy in CLL and select subtypes of relapsed or refractory NHLs. The ORR was 23% in CLL, with the most frequent grade ≥3 AEs being thrombocytopenia, neutropenia, anemia, fatigue, and hypophosphatemia.^123^ For patients with relapsed or refractory FL, MCL, and Waldenström’s macroglobulinemia (WM), a lymph node reduction of 50% or more was observed in 3 of 12 patients.^124^ The efficacy of this agent can be enhanced in combination with chemotherapy. A randomized phase 2 trial (NCT01188681) showed a significantly increased response rate and prolonged PFS of otlertuzumab in combination with bendamustine over single-agent bendamustine in relapsed CLL. The ORR of this combination therapy was 69%, with a median PFS of 15.9 months.^125^ Similarly, a phase 1 study (NCT01317901) combining otlertuzumab with BR in relapsed or refractory B-NHLs showed promising activity with no unexpected toxicity. The ORR was 83% (CR 32%).^126^

Anti-CD37 ADCs such as IMGN529 and AGS67E were also studied. IMGN529 couples an anti-CD37 antibody with the maytansine-derived anti-microtubule agent, DM1. IMGN529 has exhibited potent antitumor activity in preclinical models of CD37^+^ NHLs.^127,128^ A phase 1 trial (NCT01534715) of IMGN529 in relapsed or refractory NHLs and CLL has recently been reported, showing manageable safety profiles and preliminary evidence of activity, particularly in DLBCL.^129^ AGS67E is a fully human monoclonal IgG2 antibody conjugated via a protease-cleavable linker to MMAE. AGS67E has shown remarkable preclinical antitumor effects in NHLs and CLL cell lines and patient-derived xenograft models.^130^ Clinically, a phase 1 study (NCT02175433) of escalating doses of AGS67E as monotherapy in relapsed or refractory lymphoid malignancies is ongoing.

^177^Lu-lilotomab satetraxetan (^177^Lu-DOTA-HH1, Betalutin®) is a novel antibody radionuclide conjugate (ARC) targeting the CD37 antigen. This agent received fast channel assignment from the FDA based on the preliminary data of efficacy and safety in a phase 1/2 trial (LYMRIT 37-01, NCT01796171) in relapsed or refractory FL. It is currently in a pivotal phase 2 trial (PARADIGME) in third-line rituximab-resistant FL, while also being investigated as a single agent in a phase 1 study (NCT02658968) in relapsed or refractory DLBCL and in combination with rituximab in a phase 1 study (NCT03806179) in second-line FL treatment.

### C-C chemokine receptor type 4

C-C chemokine receptor type 4 (CCR4) is a seven-transmembrane G-protein-coupled receptor principally expressed on Th2 cells and CD4^+^ regulatory T cells,^131,132^ as well as in various types of PTCLs, including MF and ATLL.^133,134^ Furthermore, CCR4 expression was found to be an independent and significant unfavorable prognostic factor in these diseases,^133,134^ which makes it a promising target in the treatment of PTCL and ATLL.

Mogamulizumab (KW-0761, Poteligeo) is the first defucosylated humanized mAb directed against CCR4; it has been proven to induce ADCC against CCR4^+^ malignant T cells^135^ and to reduce CCR4^+^ Treg cell numbers in cutaneous T-cell lymphoma (CTCL).^136,137^ Mogamulizumab was first approved for relapsed or refractory ATLL due to its promising efficacy (ORR 50%) and acceptable toxicities in a phase 2 study (NCT00920790).^138^ In a randomized phase 2 study (NCT01173887) of dose-intensified chemotherapy with or without mogamulizumab in untreated aggressive ATLL, the mogamulizumab-containing arm showed a higher CR rate with manageable toxicities.^139^ In addition to its application in ATLL, the efficacy of mogamulizumab in CTCL has also been confirmed. A phase 1/2 study (NCT00888927) of mogamulizumab was performed on 41 pretreated patients with CTCL and resulted in an ORR of 36.8% (47.1% in SS and 28.6% in MF). The most common AEs were nausea, chills, and infusion-related reactions.^140^ A multicenter phase 2 study (NCT01192984) of relapsed CCR4^+^ PTCL and CTCL patients in Japan obtained an ORR of 35% and a median PFS of 3 months. Lymphocytopenia, leukocytopenia, and neutropenia (19%) were the most common grade 3-4 AEs.^141^ Therefore, mogamulizumab was first approved for untreated ATLL as well as relapsed or refractory PTCL in Japan.

The final results of a phase 3, randomized, multicenter clinical trial of mogamulizumab vs vorinostat in previously treated CTCL (MAVORIC, NCT01728805) have been reported.^142^ The study included 372 patients and was the largest randomized trial in CTCL. Mogamulizumab resulted in a longer PFS than vorinostat (median 7.7 months vs. 3.1 months). The most common AEs of mogamulizumab were pyrexia and cellulitis. Mogamulizumab was granted approval in the European Union and the United States for the treatment of adult patients with relapsed or refractory MF or SS after at least one prior systemic therapy.^143^

### Other surface antigens

#### CD25

CD25 (IL2R-α) is expressed on both HL and various NHLs and has been studied as a therapeutic target for over two decades. Denileukin diftitox (DD, ONTAK), a diphtheria exotoxin conjugated to an IL-2 fragment, was granted full FDA approval for the treatment of CTCL.^144^ Although the efficacy of the anti-CD25 antibodies basiliximab and daclizumab is limited, radiolabeled antibodies are promising. ^90^Y-daclizumab achieved responses in 50% of patients with relapsed HL (NCT00001575).^145^ ^90^Y-basiliximab is being evaluated in combination with carmustine, etoposide, cytarabine, melphalan (BEAM) chemotherapy for ASCT in relapsed or refractory HL (NCT01476839), as well as T-NHLs (NCT02342782). Camidanlumab tesirine (ADCT-301), a CD25 ADC, has been investigated in a phase 1 trial (NCT02432235) in patients with CD25^+^ relapsed or refractory HL and NHLs.

#### CD38

The CD38 antigen is a type II transmembrane glycoprotein with receptor and enzyme functions that is expressed in a number of hematological malignancies, particularly in multiple myeloma (MM).^146^ In addition, its expression has also been reported in lymphomas such as MCL^147^ and NK/T-cell lymphoma (NKTCL).^148^ Daratumumab is a CD38 mAb approved for treating relapsed or refractory and untreated MM. In a phase 2 study (NCT02927925) of daratumumab in relapsed or refractory NKTCL, the ORR was 35.7% in 16 patients.^149^

#### CD40

CD40 is a type-I transmembrane protein that belongs to the TNFR family. CD40 is expressed on B cells, monocytes, dendritic cells, endothelial cells and epithelial cells and plays a critical role in the regulation of immune responses.^150^ In addition, CD40 is expressed on B-NHLs, leading to the modulation of tumor cell growth after binding with its natural ligand (CD40L).^151^ Dacetuzumab (SGN-40) is a humanized IgG1 mAb targeting CD40. Although dacetuzumab has previously demonstrated anti-lymphoma activity in a phase 1 study (NCT00103779),^152^ single-agent dacetuzumab showed only modest activity in patients with relapsed DLBCL (NCT00435916)^153^ and failed to obtain higher CR rates when combined with rituximab plus ICE (R-ICE) in relapsed DLBCL in a phase 2 study (NCT00529503).^154^

#### CD74

The humanized antibody milatuzumab (hLL1) is a mAb against CD74, which is involved in malignant B-cell proliferation and survival. Preclinical studies found that milatuzumab had promising antitumor activity in NHL in vitro and in tumor xenograft models.^155^ Moreover, a phase 1/2 study (NCT00989586) delivered the anti-CD20 mAb veltuzumab (200 mg/m^2^ weekly) and escalating doses of milatuzumab to relapsed or refractory B-NHL patients and reported an ORR of 24% and a median DoR of 12 months.^156^ Another preclinical study of the novel bispecific hexavalent Abs (HexAbs) veltuzumab and milatuzumab demonstrated enhanced antitumor activity in cell lines or primary patient samples of MCL and other CD20^+^/CD74^+^ malignancies.^157^

#### CD80

CD80 (B7-1), a cell-surface receptor, is implicated in the costimulation of T-cell function and expressed on B-NHLs. The anti-CD80 mAb galiximab (IDEC-114) can inhibit tumor cells of B-NHLs in vitro and in mouse models, either alone or combined with chemotherapy (fludarabine or doxorubicin).^158^ A phase 2 study (NCT00516217) evaluated galiximab in relapsed or refractory HL and reported an ORR of 10.3%. Moreover, a phase 1/2 study on galiximab in relapsed or refractory FL revealed an ORR of 11% (CR 6%).^159^ Another phase 1/2 trial (NCT00048555) of galiximab and rituximab reported an ORR of 66% (CR 19% and unconfirmed complete remission (CRu) 14%) in relapsed or refractory FL with rituximab-refractory patients excluded.^160^

#### CD158k

CD158k (KIR3DL2) is a member of the highly polymorphic family of killer-cell immunoglobulin-like receptors (KIRs) and is expressed on NK cells and a small proportion of CD8^+^ T cells, as well as CD4^+^ T cells in CTCL.^161–163^ The anti-CD158k mAb IPH4102 has been found to be potent and safe in preclinical studies.^164^ A phase 1 study (NCT02593045) demonstrated efficacy and safety in CTCL,^165^ with the expansion study ongoing. In addition, a phase 2 study (NCT03902184) of IPH4102 alone or in combination with chemotherapy is recruiting patients with advanced T-NHLs.

#### Bispecific T cell Engagers

Bispecific T cell Engagers (BiTEs) are engineered bispecific anti-CD3 antibodies consisting of the variable domains of two antibodies linked in a single chain. A BiTE antibody binds both CD3^+^ cytotoxic T cells and a target antigen to bring the two cells into proximity and thus triggers T cells to kill tumor cells via perforin-mediated apoptosis.^166^ Blinatumomab is a CD19/CD3 BiTE that shows remarkable anti-lymphoma activity both in vitro and in vivo.^167,168^ In a phase 1 dose-escalation study (NCT00274742) in patients with relapsed or refractory NHLs, 60 μg/m^2^/day was established as the maximum tolerated dose, with 22% of patients experiencing grade 3 neurologic events. For patients treated at 60 μg/m^2^/day, the ORR was 69% (DLBCL, 55%; MCL, 71%; FL, 80%), with a median DoR of 404 days.^169^ In another phase 2 study (NCT01741792) in patients with relapsed or refractory DLBCL comparing weekly step-up dosing with flat dosing, the ORR was 43%. However, neurological AEs are also common.^170^ A later phase 2 trial (NCT02910063) of blinatumomab in aggressive B-NHLs is ongoing.

In addition, trials on anti-CD20/CD3 bispecific antibodies, including mosunetuzumab (BTCT4465A, NCT03677154, NCT03671018 and NCT03677141), RO7082859 (NCT03075696, NCT03533283 and NCT03467373), REGN1979 (NCT03888105, NCT02290951, and NCT02651662) and XmAb13676 (NCT02924402) are currently ongoing.

In summary, therapies targeting the lymphoma surface antigen have made great progress. In general, mAbs are effective in the treatment of lymphoma, as evidenced by the FDA accelerated approval of many drugs. Moreover, mAbs as monotherapy have fewer adverse reactions and higher tolerance than conventional chemotherapy. However, mAbs also have limitations, such as off-target effects. In the future, more research on the precise mechanisms of the efficacy and resistance of mAbs is needed. The design of future clinical trials should focus on subgroups with specific pathogenic mechanisms. At the same time, attention should also be paid to the timing, duration, and dose optimization of mAbs, either alone or in combination with traditional chemotherapy.

## Signaling transduction pathways and targeted therapies

Signaling transduction pathways are critically involved in lymphoma progression. Inhibitors targeting key pathways, including spleen tyrosine kinase (SYK), Bruton’s tyrosine kinase (BTK), phosphoinositide 3-kinase (PI3K)/AKT/mammalian target of rapamycin (mTOR), Janus kinase-signal transducer and activator of transcription (JAK-STAT), NOTCH, NF-κB and ubiquitin-proteasome pathway (UPP), have been applied to treat lymphomas.

### SYK

**Fig. 1:**
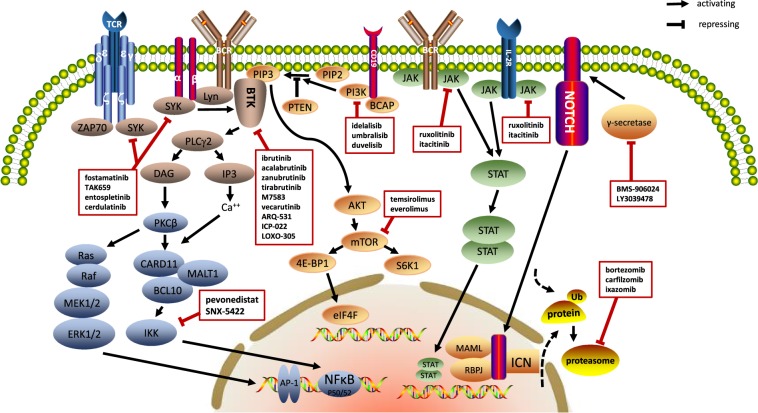
Signaling transduction pathways in lymphoma cells

SYK, a nonreceptor tyrosine kinase, plays an important role in BCR and T-cell receptor (TCR) signaling. The phosphorylation of immunoreceptor tyrosine-based activation motifs (ITAMs) in the Igα (CD79A)/Igβ (CD79B) cytoplasm region recruits SYK and induces SYK activation, BTK recruitment, and phospholipase Cγ2 (PLCγ2) activation.^171^ In TCR signaling, phosphorylated CD3 and ζ subunits of the TCR complex by the Src-related kinases LCK and FYN recruit zeta-chain-associated protein kinase 70 (ZAP-70) and SYK (Fig. 1).^172^

The activated B cell-like subtype of DLBCL (ABC-DLBCL) is characterized by antigen-driven BCR signaling,^173,174^ while germinal center B cell-like (GCB)-DLBCL features tonic, antigen-independent BCR signaling.^175,176^ BL is also characterized by tonic BCR signaling and mostly relies on SYK.^177^ In T-NHLs, aberrant SYK expression was reported in monomorphic epitheliotropic intestinal T-cell lymphomas (MEITL, type II EATL),^178^ the follicular variant of PTCL, not otherwise specified (PTCL-NOS), and angioimmunoblastic T-cell lymphoma (AITL) due to t(5;9)(q33;q22) *ITK*/*SYK* translocation.^179–181^

The targeted agents and clinical trials related to SYK and BTK are listed in Table 2. Fostamatinib disodium, the first approved oral SYK inhibitor, was evaluated in a phase 1/2 trial (NCT00446095) of recurrent B-NHLs, showing an ORR of 22% in DLBCL, 10% in FL, and 11% in MCL.^182^ TAK-659 is being studied in a phase 2 trial in relapsed or refractory DLBCL (NCT03123393) alone, in combination with venetoclax in NHLs in a phase 1 trial (NCT03357627), and in combination with R-CHOP in DLBCL in a phase 1 trial (NCT03742258). The efficacy of entospletinib (GS-9973) is being explored in a phase 2 trial (NCT01799889) in relapsed or refractory hematologic malignancies alone as well as in combination with obinutuzumab in a phase 1/2 trial in NHLs (NCT03010358). Another phase 2 study (NCT01796470) of entospletinib combined with idelalisib in relapsed or refractory NHLs and CLL underwent early termination due to treatment-emergent pneumonitis in 18% of patients.^183^ Cerdulatinib (PRT-062070), a dual SYK/JAK inhibitor, was reported to have a greater capacity to suppress cell proliferation and induce apoptosis than PRT-060318, an SYK-selective inhibitor, in ATLL-derived cell lines and murine models.^184^ A phase 1/2 trial (NCT01994382) of cerdulatinib in NHLs and CLL/SLL and a phase 2 trial (NCT04021082) of cerdulatinib in relapsed or refractory PTCL are ongoing.

**Table 2:** Targeted drugs and clinical trials related to SYK and BTK

Drug	Disease	Trial name	Phase	Status	ORR/CR	NCT#	Reference
*SYK inhibitor*							
*Fostamatinib*	*A SYK inhibitor*						
Fostamatinib	Relapsed or refractory B-NHLs	Efficacy and safety study of fostamatinib tablets to treat B-cell lymphoma	1/2	Completed	DLBCL, 22%; FL, 10%; MCL, 11%	NCT00446095	^182^
*TAK-659*	*A SYK inhibitor*						
TAK-659	Relapsed or refractory DLBCL	TAK-659 in participants with relapsed or refractory diffuse large B-cell lymphoma	2	Active, not recruiting	–	NCT03123393	–
TAK-659, venetoclax	Relapsed or refractory NHL	A study of TAK-659 in combination with venetoclax for adult participants with previously treated non-Hodgkin’s lymphoma	1	Active, not recruiting	–	NCT03357627	–
TAK-659, R-CHOP	High-risk DLBCL	Combination chemotherapy and TAK-659 as frontline treatment in treating patients with high-risk diffuse large B-cell lymphoma	1	Recruiting	–	NCT03742258	–
*Entospletinib*	*A SYK inhibitor*						
Entospletinib	Relapsed or refractory hematologic malignancies	Entospletinib in adults with relapsed or refractory hematologic malignancies	2	Active, not recruiting	–	NCT01799889	–
Entospletinib, obinutuzumab	Relapsed or refractory CLL/SLL, NHL	Entospletinib and obinutuzumab in treating patients with relapsed chronic lymphocytic leukemia, small lymphocytic lymphoma, or non-Hodgkin’s lymphoma	1/2	Recruiting	–	NCT03010358	–
Entospletinib, idelalisib	Relapsed or refractory hematologic malignancies	Entospletinib in combination with idelalisib in adults with relapsed or refractory hematologic malignancies	2	Terminated	–	NCT01796470	^183^
*Cerdulatinib*	*A dual SYK/JAK inhibitor*						
Cerdulatinib	CLL/SLL, NHL	Phase 1/2 dose-escalation study in CLL/SLL or NHL	1/2	Recruiting	–	NCT01994382	–
Cerdulatinib	Relapsed or refractory PTCL	CELTIC-1: a phase 2/3 study of cerdulatinib in patients with relapsed or refractory peripheral T-cell lymphoma	2/3	Not yet recruiting	–	NCT04021082	–
*BTK inhibitor*							
*Ibrutinib*	*Suppressing BTK enzymatic activity through a irreversible covalent bond with a cysteine residue in the BTK active site*						
Ibrutinib	Relapsed or refractory B-NHLs	Study of the safety and tolerability of ibrutinib in patients with recurrent B-cell lymphoma	1/2	Completed	60%/16%	NCT00849654	^193^
Ibrutinib	Relapsed or refractory DLBCL	Safety and efficacy study of a Bruton’s tyrosine kinase inhibitor in subjects with relapsed or refractory diffuse large B-cell lymphoma	1/2	Completed	ABC-DLBCL, 37%/16%; GCB-DLBCL, 5%/0%	NCT01325701	^194^
Ibrutinib	Relapsed or refractory FL	Ibrutinib in treating patients with relapsed or refractory follicular lymphoma	2	Active, not recruiting	37.5%/12.5%	NCT01849263	^195^
Ibrutinib	Relapsed or refractory MZL	Study of the Bruton’s tyrosine kinase inhibitor in subjects with relapsed or refractory marginal zone lymphoma	2	Completed	48%/3%	NCT01980628	^196^
Ibrutinib	Relapsed or refractory MCL	Safety and efficacy of ibrutinib in participants with relapsed or refractory mantle cell lymphoma	2	Completed	68%/21%	NCT01236391	^197^
Ibrutinib, nivolumab	Relapsed or refractory B-NHLs, CLL/SLL	A study to evaluate safety, pharmacokinetics, pharmacodynamics and preliminary efficacy of the combination of ibrutinib with nivolumab in participants with hematologic malignancies	1/2	Active, not recruiting	DLBCL, 36%/16%; FL, 33%/10%; CLL/SLL, 61%/0%	NCT02329847	^198^
Ibrutinib, venetoclax	MCL	Venetoclax plus ibrutinib in mantle cell lymphoma (AIM)	2	Completed	71%/62%	NCT02471391	^199^
Ibrutinib, lenalidomide, rituximab	Untreated and unfit elderly DLBCL	Study evaluating the safety and efficacy of ibrutinib, lenalidomide, and rituximab in untreated and unfit elderly patients with DLBCL	2	Recruiting	–	NCT03949062	–
Ibrutinib, lenalidomide, rituximab	Untreated FL	Ibrutinib, lenalidomide, and rituximab in treating patients with previously untreated stage II–IV follicular lymphoma	1	Active, not recruiting	95%/NA	NCT01829568	^200^
Ibrutinib, lenalidomide, rituximab	Relapsed or refractory MCL	A trial of ibrutinib, lenalidomide, and rituximab for patients with relapsed or refractory mantle cell lymphoma (PHILEMON)	2	Recruiting	76%/56%	NCT02460276	^201^
Ibrutinib, R-CHOP	Untreated CD20^+^ B-NHLs	A study combining ibrutinib with rituximab, cyclophosphamide, doxorubicin, vincristine, and prednisone in patients With CD20^+^ B-cell non-Hodgkin’s lymphoma	1	Completed	100%/NA	NCT01569750	^202^
Ibrutinib, R-CHOP vs. placebo, R-CHOP	Untreated non-GCB DLBCL	A study of the Bruton’s tyrosine kinase inhibitor, ibrutinib, in combination with rituximab, cyclophosphamide, doxorubicin, vincristine, and prednisone in patients with newly diagnosed non-germinal center B-cell subtype of diffuse large B-cell lymphoma	3	Active, not recruiting	Ibrutinib, R-CHOP, NA/67.3%; placebo, R-CHOP, NA/68.0%	NCT01855750	–
Ibrutinib, high-dose methotrexate, rituximab	Relapsed or refractory CNSL	Bruton’s tyrosine kinase inhibitor, ibrutinib, in patients with relapsed or refractory primary central nervous system lymphoma and relapsed or refractory secondary central nervous system lymphoma	1/2	Active, not recruiting	Phase 1 part: 80%/53%	NCT02315326	^203^
*Acalabrutinib*	*A new, irreversible and second-generation BTK inhibitor with enhanced efficacy and improved off-target effect*						
Acalabrutinib	Relapsed or refractory MCL	An open-label, phase 2 study of acalabrutinib in subjects with mantle cell lymphoma	2	Active, not recruiting	81%/40%	NCT02213926	^205^
Acalabrutinib	Relapsed CLL	Acalabrutinib, a novel bruton tyrosine kinase inhibitor, for treatment of chronic lymphocytic leukemia	1/2	Active, not recruiting	95%/0%	NCT02029443	^206^
Acalabrutinib vs. ibrutinib	Previously treated high-risk CLL	Study of acalabrutinib versus ibrutinib in previously treated subjects with high-risk CLL	3	Active, not recruiting	–	NCT02477696	–
Acalabrutinib, pembrolizumab	Hematologic malignancies	Acalabrutinib in combination with pembrolizumab, for treatment of hematologic malignancies (KEYNOTE145)	1/2	Active, not recruiting	–	NCT02362035	–
Acarabrutinib, venetoclax	Relapsed or refractory MCL	Acalabrutinib and venetoclax in treating patients with relapsed or refractory mantle cell lymphoma	2	Recruiting	–	NCT03946878	–
Acalabrutinib, BR vs. placebo, BR	Untreated MCL	A study of bendamustine and rituximab alone versus in combination with acalabrutinib in subjects with previously untreated mantle cell lymphoma	3	Recruiting	–	NCT02972840	–
Acalabrutinib, R-CHOP	Untreated DLBCL	A combination of acalabrutinib with R-CHOP for patient with diffuse large B-cell lymphoma (ACCEPT)	1/2	Recruiting	–	NCT03571308	–
acalabrutinib, R-ICE	relapsed or refractory DLBCL	Acalabrutinib plus R-ICE for relapsed or refractory diffuse large B-cell lymphoma	2	Not yet recruiting	–	NCT03736616	–
*Zanubrutinib*	*A second-generation BTK inhibitor showing distinguished kinase selectivity and lower side effect*						
Zanubrutinib	B-cell lymphoid malignancies	Study of the safety and pharmacokinetics of zanubrutinib in subjects with B-cell lymphoid malignancies	1	Active, not recruiting	total, 96.2%/2.6%; treatment-naïve, 100%/4.5%; relapsed or refractory, 94.6%/1.8%	NCT02343120	^207^
Zanubrutinib	Relapsed or refractory non-GCB DLBCL	Study of BTK inhibitor zanubrutinib in subjects with relapsed or refractory non-GCB type diffuse large B-cell lymphoma	2	Active, not recruiting	–	NCT03145064	–
Zanubrutinib	Relapsed or refractory MZL	Study of zanubrutinib in patients with marginal zone lymphoma	2	Recruiting	–	NCT03846427	–
Zanubrutinib	Relapsed or refractory MCL	Study to evaluate efficacy and safety of zanubrutinib in subjects with relapsed or refractory mantle cell lymphoma	2	Active, not recruiting	–	NCT03206970	–
Zanubrutinib vs. ibrutinib	Relapsed or refractory CLL	A study of zanubrutinib versus ibrutinib in patients with relapsed or refractory chronic lymphocytic leukemia (ALPINE)	3	Recruiting	–	NCT03734016	–
Zanubrutinib vs. ibrutinib	WM	A study comparing zanubrutinib and ibrutinib in subjects with Waldenström’s macroglobulinemia	3	Active, not recruiting	–	NCT03053440	–
*Tirabrutinib*	*A highly selective irreversible BTK inhibitor*						
Tirabrutinib	relapsed or refractory NHLs/CLL	Phase 1 study of tirabrutinib given as monotherapy in patients with relapsed or refractory NHLs and CLL	1	Completed	ABC-DLBCL, 35%/9.7%; MCL, 92%/46%; CLL, 96%/NA	NCT01659255	^208^
*M7583*	*A novel irreversible BTK inhibitor*						
M7583	B-cell malignancies	BTK inhibitor in B-cell malignancies	1/2	Active, not recruiting	–	NCT02825836	–
*Vecarutinib*	*A noncovalent or reversible BTK inhibitor*						
Vecarutinib	B-NHLs	Safety and antitumor activity of vecabrutinib in B-lymphoid cancers	1/2	Recruiting	–	NCT03037645	–
*ARQ-531*	*A reversible BTK inhibitor with off-target activity against Src and Tec family of protein tyrosine kinases*						
ARQ-531	Hematologic malignancies	Safety and antitumor activity of ARQ-531 in hematologic malignancies	1	Recruiting	–	NCT03162536	–
*ICP-022*	*A novel BTK inhibitor*						
ICP-022	Relapsed or refractory B-cell malignancies	Dose escalation of ICP-022 in patients with relapsed or refractory B-cell malignancies	1	Recruiting	–	NCT04014205	–
*LOXO-305*	*A novel, selective noncovalent or reversible BTK inhibitor*						
LOXO-305	CLL/SLL, NHLs	A study of oral LOXO-305 in patients with previously treated CLL/SLL or NHLs	1/2	Recruiting	–	NCT03740529	–

NA: ORR or CR are not available on the clinicaltrials.gov or from the published article although the trial has been completed

*R-CHOP* rituximab, cyclophosphamide, doxorubicin, vincristine, prednisolone, *BR* bendamustine and rituximab, *R-ICE* rituximab, ifosfamide, carboplatin, etoposide

### BCR-BTK

The activation of BCR leads to the phosphorylation of LYN and SYK, which phosphorylate tyrosine residues in the cytoplasmic part of CD19 and B-cell adaptor for PI3K (BCAP), inducing PI3K activation, phosphatidylinositol 4,5-bisphosphate (PIP2) transformation to phosphatidylinositol 3,4,5-trisphosphate (PIP3), and BTK recruitment. BTK activation leads to PLCγ2 phosphorylation, which could further hydrolyze PIP2 to produce 1,4,5-trisphosphate (IP3) and diacylglycerol (DAG).^185^ IP3 is involved in intracellular calcium regulation and nuclear factor of activated T cells (NFAT) transcription, and DAG is associated with protein kinase Cβ (PKCβ) and mitogen-activated protein kinase (MAPK) family activation.^186^ PKCβ also participates in the NF-κB pathway through a scaffold complex including CARD11, BCL-10, and MALT1. BTK plays a key role in the tonic BCR signaling pathway through the positive regulation of AKT phosphorylation (Fig. 1).^187^ The inhibition of BTK decreased BTK^Y223^ phosphorylation and anti-apoptotic protein expression (BCL-2, BCL-XL, and MCL-1), resulting in increased apoptosis in MCL cell lines.^188^ Moreover, recurrent gene mutations of the BCR-BTK signaling pathway are frequently found in ABC-DLBCL, FL, and marginal zone lymphoma (MZL).^4,189–192^

Ibrutinib is an irreversible BTK inhibitor that suppresses BTK enzymatic activity through a covalent bond with a cysteine residue in the BTK active site. A phase 1/2 study (NCT00849654) of ibrutinib enrolled patients with relapsed or refractory B-NHLs and reported promising safety and response (ORR 60% and CR 16%).^193^ In a phase 1/2 trial (NCT01325701) of relapsed or refractory DLBCL, ibrutinib induced an ORR of 37% in ABC-DLBCL but only an ORR of 5% in GCB-DLBCL.^194^ A phase 2 trial (NCT01849263) of ibrutinib in relapsed or refractory FL reported an ORR of 37.5% (CR 12.5%).^195^ Ibrutinib has also been actively investigated in other relapsed or refractory B-NHLs and has shown clinical efficacy (NCT01980628 and NCT01236391).^196,197^ A phase 1/2 trial (NCT02329847) of ibrutinib in combination with nivolumab in relapsed or refractory B-cell malignancies revealed an ORR of 36% in DLBCL (CR 16%), 33% in FL (CR 10%), and 61% in CLL/SLL (CR 0%).^198^ Moreover, a phase 2 study (NCT02471391) of ibrutinib combined with venetoclax in MCL reported an ORR of 71% (CR 62%).^199^ The combination of ibrutinib, lenalidomide, and rituximab is being explored in a phase 2 trial (NCT03949062) to evaluate its efficacy and safety in untreated and unfit elderly DLBCL patients. This combination also induced an ORR of 95% in untreated FL in a phase 1 trial (NCT01829568), as well as an ORR of 76% (CR 56%) in relapsed or refractory MCL in a phase 2 trial (NCT02460276).^200,201^ In untreated CD20^+^ B-NHLs, ibrutinib plus R-CHOP achieved an ORR of 100% in a phase 1 study (NCT01569750).^202^ In addition, in a phase 3 study (NCT01855750) in untreated non-GCB DLBCL, ibrutinib plus R-CHOP produced a CR rate of 67.3%, and placebo plus R-CHOP produced a CR rate of 68.0%, with no statistically significant difference. Moreover, the sequential combination of ibrutinib with high-dose methotrexate and rituximab was studied in patients with primary central nervous system lymphoma (PCNSL) (NCT02315326).^203^

Acalabrutinib (ACP-196) is a BTK inhibitor that has been proven to have a more enhanced efficacy than ibrutinib in canine studies.^204^ A phase 2 study (NCT02213926) reported an ORR of 81% (CR 40%) in relapsed or refractory MCL.^205^ The FDA has approved acalabrutinib for treating relapsed or refractory MCL. Moreover, in a phase 1/2 trial (NCT02029443) of acalabrutinib in relapsed CLL, the ORR was 95%, and a 100% ORR was obtained among patients with chromosome 17p13.1 deletion.^206^ A phase 3 trial (NCT02477696) of acalabrutinib vs ibrutinib in high-risk CLL is ongoing. Trials on acalabrutinib in combination with pembrolizumab (NCT02362035), venetoclax (NCT03946878), BR (NCT02972840), R-CHOP (NCT03571308), or R-ICE (NCT03736616) in hematological malignancies are ongoing. Zanubrutinib (BGB-3111) is a second-generation BTK inhibitor that has a promising ORR (96.2%) with low toxicity in CLL/SLL patients in a phase 1 trial (NCT02343120).^207^ Phase 2 trials of zanubrutinib in relapsed or refractory DLBCL (NCT03145064), MZL (NCT03846427), and MCL (NCT03206970), as well as phase 3 trials (NCT03734016 and NCT03053440) comparing zanubrutinib with ibrutinib in patients with relapsed or refractory CLL or WM, are ongoing. Tirabrutinib (ONO/GS-4059), a highly selective irreversible BTK inhibitor, achieved a response of 35%, 92%, and 96% in relapsed or refractory ABC-DLBCL, MCL, and CLL patients, respectively, in a phase 1 trial (NCT01659255).^208^ M7583, a novel irreversible BTK inhibitor, is being explored in a phase 1/2 trial (NCT02825836) in patients with relapsed or refractory B-cell malignancies. Vecabrutinib (SNS-062), a noncovalent or reversible BTK inhibitor, suppresses both wild-type and C481S-mutated BTK activity and is being investigated in a phase 1/2 trial (NCT03037645) in B-NHLs. ARQ-531 is another reversible BTK inhibitor with off-target activity against the Src and Tec family of protein tyrosine kinases. Compared with ibrutinib, ARQ-531 has a better capacity to reduce CLL cell viability in mice.^209^ In addition, a phase 1 trial (NCT03162536) of ARQ-531 in patients with hematological malignancies is ongoing. Trials on ICP-022 and LOXO-305, which are also novel BTK inhibitors, are recruiting patients with refractory B-cell malignancies (NCT04014205 and NCT03740529).

### PI3K-AKT-mTOR

The PI3K-AKT-mTOR pathway is an important regulator in normal myeloid and lymphoid development.^210^ Upon activation, BCAP is upregulated, and the catalytic subunit of PI3K (referred to as p110α, p110β, p110γ, and p110δ for the four different isoforms) triggers PIP3 and recruits the serine/threonine kinase AKT to the plasma membrane.^211^ AKT can subsequently activate mTOR, which encompasses two different multiprotein complexes, mTOR complex 1 (mTORC1) and mTOR complex 2 (mTORC2). mTORC1 phosphorylates 4E-BP1 and S6K1 to activate key drivers of protein translation (Fig. 1).^212^ Downstream signaling of BCR is largely dependent on p110δ, and mutations in *PIK3CA* (the gene encoding p110α) were found in approximately 1–8% of DLBCL.^213,214^ In T-NHLs, p110δ and p110γ are vital kinases of TCR signaling and chemokine receptor signaling, respectively.^215–217^

The targeted drugs and clinical trials related to the PI3K-AKT-mTOR, JAK-STAT, NOTCH, and NF-κB signaling pathways are listed in Table 3. Idelalisib (CAL-101, GS-1101), a p110δ-selective inhibitor, is the first FDA-approved PI3K inhibitor in treating relapsed FL and SLL. A phase 2 trial (NCT01393106) demonstrated that idelalisib was tolerable and had modest single-agent activity in relapsed or refractory HL (ORR 68% and CR 4%).^218^ Another phase 2 trial (NCT01282424) of idelalisib treated indolent NHLs including FL, SLL, MZL, and lymphoplasmacytic lymphoma (LPL) with or without WM and showed antitumor activity with an acceptable safety profile (ORR 57% and CR 6%).^219^ Moreover, combinations of idelalisib with other novel agents may improve the response rate and DoR. Studies of idelalisib in combination with obinutuzumab in relapsed or refractory FL (NCT03890289) and in combination with BR in indolent B-NHLs and MCL (NCT01088048 and NCT01090414) are ongoing. However, two phase 1 trials of idelalisib and lenalidomide in patients with recurrent FL (NCT01644799) and MCL (NCT01838434) showed emerging toxicities as new combinations.^220^ Umbralisib (TGR-1202) and parsaclisib (INCB050465) are also p110δ inhibitors with different chemical structures.^221^ A phase 1 trial (NCT02268851) of umbralisib and ibrutinib showed an ORR of 67% (CR 19%) in relapsed or refractory MCL.^222^ Duvelisib (IPI-145/INK1197), which is an inhibitor of both p110δ and p110γ, showed efficacy in various types of lymphomas, including DLBCL and MCL, in preclinical studies.^223,224^ A phase 2 trial (NCT01882803) of duvelisib monotherapy in relapsed or refractory indolent NHLs demonstrated an ORR of 46% (41% in FL, 33% in MZL, and 68% in SLL).^225^

**Table 3:** Targeted drugs and clinical trials related to the PI3K-AKT-mTOR, JAK-STAT, NOTCH, and NF-κB signaling pathways

Drug	Disease	Trial name	Phase	Status	ORR/CR	NCT#	Reference
*PI3k-AKT-mTOR pathway*							
*Idelalisib*	*A p110δ-selective inhibitor*						
Idelalisib	Relapsed or refractory HL	Safety and efficacy of idelalisib in relapsed or refractory Hodgkin’s lymphoma	2	Completed	68%/4%	NCT01393106	^218^
Idelalisib	Idolent B-NHLs	Efficacy and safety study of idelalisib in subjects with indolent B-cell non-Hodgkin’s lymphoma (DELTA)	2	Completed	57%/6%	NCT01282424	^219^
Idelalisib, obinutuzumab	Relapsed or refractory FL	Idelalisib plus obinutuzumab in patients with relapsed or refractory follicular lymphoma (GAUDEALIS)	2	Not yet recruiting	–	NCT03890289	–
Idelalisib, BR	Relapsed or refractory indolent B-NHLs/MCL/CLL	Study to investigate idelalisib in combination with chemotherapeutic agents, immunomodulatory agents and anti-CD20 monoclonal antibody in subjects with relapsed or refractory indolent B-cell non-Hodgkin’s lymphoma, mantle cell lymphoma or chronic lymphocytic leukemia	1	Completed	81%/32%	NCT01088048, NCT01090414	–
Idelalisib, lenalidomide	Recurrent FL	Lenalidomide and idelalisib in treating patients with recurrent follicular lymphoma	1	Completed	NA	NCT01644799	^220^
Idelalisib, lenalidomide	Relapsed or refractory MCL	Lenalidomide with or without idelalisib in treating patients with relapsed or refractory mantle cell lymphoma	1	Completed	NA	NCT01838434	^220^
*Umbralisib*	*A second-generation p110δ-selective inhibitor*						
Umbralisib, ibrutinib	CLL/MCL	A phase 1 safety and efficacy study of the PI3K-delta Inhibitor umbralisib and ibrutinib in patients with CLL or MCL	1	Completed	MCL, 67%/19%; CLL, 90%/29%	NCT02268851	^222^
*Duvelisib*	*A PI3Kδ/γ inhibitor*						
Duvelisib	Refractory iNHLs	A phase 2 study of duvelisib in subjects with refractory indolent non-Hodgkin’s lymphoma (DYNAMO)	2	Active, not recruiting	46%/NA	NCT01882803	^225^
*Temsirolimus*	*A mTOR inhibitor*						
Temsirolimus	MCL	Study evaluating temsirolimus in mantle cell lymphoma (OPTIMAL)	3	Completed	38%/3%	NCT00117598	^226^
Temsirolimus vs. ibrutinib	Relapsed or refractory MCL	Study of ibrutinib versus temsirolimus in patients with relapsed or refractory mantle cell lymphoma who have received at least one prior therapy	3	Completed	NA	NCT01646021	^227^
Temsirolimus, rituximab, DHAP	Relapsed or refractory DLBCL	Temsirolimus, rituximab, and DHAP for relapsed or refractory diffuse large B-cell lymphoma (STORM)	2	Unknown	–	NCT01653067	^228^
*Everolimus*	*An oral mTOR inhibitor*						
Everolimus, itacitinib	HL	Everolimus plus itacitinib in Hodgkin’s lymphoma	1/2	Recruiting	–	NCT03697408	–
Everolimus, panobinostat	Relapsed or refractory lymphoma	Everolimus plus panobinostat in patients with relapsed or refractory lymphoma	1/2	Completed	NA	NCT00967044	–
*JAK-STAT pathway*							
*Ruxolitinib*	*A JAK1/2 inhibitor*						
Ruxolitinib	Relapsed or refractory cHL	A phase 2 study of oral JAK1/JAK2 inhibitor ruxolitinib in adult patients with relapsed or refractory classical Hodgkin’s lymphoma (HIJAK)	2	Completed	9.4%/0%	NCT01877005	^245^
Ruxolitinib, nivolumab	Relapsed or refractory cHL	Nivolumab with ruxolitinib in relapsed or refractory classical Hodgkin’s lymphoma	1	Recruiting	–	NCT03681561	–
*Itacitinib*	*A JAK1 selective inhibitor*						
Itacitinib, everolimus	HL	Itacitinib plus everolimus in Hodgkin’s lymphoma	1/2	Recruiting	–	NCT03697408	–
Itacitinib, ibrutinib	Relapsed or refractory DLBCL	A study of itacitinib in combination with ibrutinib in subjects with relapsed or refractory diffuse large B-cell lymphoma	1/2	Active, not recruiting	–	NCT02760485	–
*NOTCH signaling pathway*							
*BMS-906024*	*A γ-secretase inhibitor*						
BMS-906024, dexamethasone	T-ALL/T-LBL	Study to evaluate the safety and tolerability of weekly intravenous doses of BMS-906024 in subjects with acute T-cell lymphoblastic leukemia or T-cell lymphoblastic lymphoma	1	Completed	NA	NCT01363817	–
*LY3039478*	*A γ-secretase inhibitor*						
LY3039478, dexamethasone	T-ALL/T-LBL	A study of LY3039478 in combination with dexamethasone in participants with acute T-cell lymphoblastic leukemia or T-cell lymphoblastic lymphoma	1/2	Completed	–	NCT02518113	–
*CB-103*	*A pan-NOTCH inhibitor*						
CB-103	Solid tumors/NHLs	Study of CB-103 in adult patients with advanced or metastatic solid tumors and hematological malignancies	1/2	Recruiting	–	NCT03422679	–
*NF-κB pathway*							
*Pevonedistat*	*A NEDD8-activating enzyme inhibitor*						
Pevonedistat, ibrutinib	Relapsed or refractory CLL/NHL	Pevonedistat and ibrutinib in treating participants with relapsed or refractory chronic lymphocytic leukemia or non-Hodgkin’s lymphoma	1	Recruiting	–	NCT03479268	–
*SNX-5422*	*A synthetic, novel, small-molecule HSP90 Inhibitor*						
SNX-5422	Solid tumors/lymphomas	Safety study of SNX-5422 to treat solid tumor cancers and lymphomas	1	Completed	NA	NCT00647764	–
SNX-5422	Solid tumors/lymphomas	SNX-5422 to treat solid tumor cancers and lymphoma	1	Completed	NA	NCT00644072	–

NA: ORR or CR are not available on the clinicaltrials.gov or from the published article, although the trial has been completed

*DHAP* dexamethasone, high-dose cytarabine, cisplatin, *T-ALL* acute T-cell lymphoblastic leukemia

Temsirolimus (CCI-779) is a derivative of rapamycin, and a phase 2 trial (NCT00117598) of temsirolimus as a single agent in relapsed MCL showed an ORR of 38% (CR 3%).^226^ In a randomized phase 3 trial (NCT01646021) enrolling patients with relapsed or refractory MCL, significant improvement in PFS and better tolerance were observed in patients treated with ibrutinib vs temsirolimus.^227^ Another ongoing study (NCT01653067) is evaluating temsirolimus in combination with DHAP in patients with relapsed or refractory DLBCL.^228^ Everolimus (RAD001) is an oral mTOR inhibitor that has been used as a single agent in relapsed or refractory aggressive and indolent NHLs as well as HL.^229–231^ Clinical trials (NCT03697408 and NCT00967044) to assess everolimus combined with other agents, such as itacitinib and panobinostat, are recruiting patients.

### JAK-STAT

The JAK-STAT pathway is activated by extracellular cytokines such as interferons, IL-2, IL-6 and growth factors, which regulate cell survival, proliferation, differentiation, and apoptosis.^232,233^ There are four cytoplasmic JAK kinases: JAK1, JAK2, JAK3, and TYK2. JAK1/JAK3 are prone to immunoregulation, while JAK2 is associated with erythrocyte and platelet formation.^234,235^ JAKs lead to STAT phosphorylation, homodimerization, and nuclear translocation (Fig. 1).^233,236^ There are seven STAT proteins (STAT1, STAT2, STAT3, STAT4, STAT5A, STAT5B, and STAT6).^234,235^ The activation of the JAK/STAT signaling pathway, as assessed by STAT3 or STAT5B phosphorylation, was present in T-NHLs, including anaplastic lymphoma kinase (ALK)-positive and ALK-negative ALCL,^237,238^ HTLV-1-associated ATLL,^239,240^ and NKTCL.^241,242^ Twenty percent of ALK-negative ALCL patients present mutations of the JAK1 and/or STAT3 genes,^237^ and approximately 10% of NKTCL patients present STAT3 mutations.^243^

Ruxolitinib (INCB018424) is a JAK1/2 inhibitor approved by the FDA to treat myelofibrosis. Ruxolitinib significantly enhanced apoptosis in HL and PMBCL in vitro and promoted survival in a lymphoma xenograft murine model.^244^ A phase 2 study (NCT01877005) of ruxolitinib in advanced relapsed or refractory HL showed poor efficacy as monotherapy (ORR 9.4% and CR 0%).^245^ Ruxolitinib and navitoclax, a Bcl-2/Bcl-XL inhibitor, reduced the tumor burden and prolonged survival in an ATLL xenograft murine model.^246^ A phase 1 study (NCT03681561) of ruxolitinib in combination with nivolumab in relapsed or refractory HL is currently recruiting patients. However, ruxolitinib has off-target effects due to JAK2 inhibition, which may lead to thrombocytopenia, anemia, and neutropenia.^247^ Therefore, agents that can selectively inhibit JAK1, such as itacitinib (INCB039110), are expected to better treat lymphomas in view of the risk-benefit ratio. A phase 1/2 study (NCT03697408) of itacitinib in combination with everolimus in relapsed or refractory HL is ongoing. In addition, a phase 1/2 trial (NCT02760485) of itacitinib in combination with ibrutinib in subjects with relapsed or refractory DLBCL is also active.

### NOTCH

NOTCH receptors are single-pass type I transmembrane proteins. Four receptors (NOTCH1-4) are expressed in mammals and share a common structure. Among them, NOTCH1 and NOTCH2 are the most widely expressed receptors and play a role in cell growth, proliferation, survival, and differentiation.^248^ NOTCH is cleaved in the transmembrane region by the γ-secretase complex, which can be inhibited by small-molecule γ-secretase inhibitors (GSIs). After release from the membrane, the intracellular portion of the NOTCH receptor translocates to the nucleus, where it interacts with the RBPJ DNA-binding protein and recruits the MAML1 transcriptional coactivator to assemble the transcriptional complex and start transcription. The signal can be terminated by the proteasome (Fig. 1).^249^ Mutations of NOTCH1 and NOTCH2 have been reported to mediate the differentiation of B- or T-cell lineages.^250^ In T-cell lymphoblastic lymphoma (T-LBL), *NOTCH1* mutations vary from 30% to 80%.^251^ In DLBCL, *NOTCH1* mutations are classified into the N1 subtype, which accounts for 6.1% of ABC DLBCL cases and is associated with poor prognosis.^252^ Activation of the NOTCH1 pathway was also observed in MCL, HL and BL.^253–255^ *NOTCH2* mutations are present in approximately 25% of patients with splenic marginal zone lymphoma (SMZL) and approximately 5% of patients with non-splenic MZL^253^ and are related to adverse clinical outcomes.^253,256,257^ In addition, a similar gene profile has been found in FL.^258^ In DLBCL, the BN2 subtype is characterized by *BCL6* fusions and *NOTCH2* mutations and presents a relatively good prognosis.^252^

For targeted agents of the NOTCH pathway, GSIs, as well as antibodies against NOTCH, Delta/Jagged ligands, or other extracellular components involved in the NOTCH signaling cascade, have been tested in multiple clinical trials.^259^ GSIs can suppress the release of ICN1 from the membrane and effectively abrogate the activation of NOTCH1 transcriptional programs in cell lines.^260^ A phase 1 trial (NCT01363817) evaluating the safety and tolerability of BMS-906024 in subjects with T-LBL was completed. Another study showed strong synergy between glucocorticoids and GSIs.^261^ A phase 1/2 trial (NCT02518113) to evaluate LY3039478 in combination with dexamethasone in T-LBL patients was also completed. However, GSIs demonstrated dose-limiting goblet cell hyperplasia of the gut, mainly due to the inhibition of both NOTCH1 and NOTCH2 expression on these tissues.^262^ In addition, a phase 1/2 trial (NCT03422679) to investigate the safety, tolerability, and preliminary efficacy of CB-103, a pan-NOTCH inhibitor, is recruiting patients. More research and clinical trials are needed to better understand targeted therapy of the NOTCH pathway.

### NF-κB

The NF-κB pathway is one of the key signaling pathways implicated in physiological cellular functions and neoplastic processes.^263,264^ Core components of the NF-κB pathway are inhibitors of NF-κB (IκB) proteins, the IκB kinase (IKK) complex, and NF-κB transcription factors, which include RelA/p65, RelB, c-Rel/Rel, p50, and p52.^265^ B-cell associated kinases (BAKs), such as BTK or PI3Kδ, are critical signaling transducers of BCR signaling and can trigger a cascade reaction to form a multiprotein CARD11-BCL-10-MALT1 (CBM) complex.^266^ This complex interacts with IKK, the upstream molecule of NF-κB, and promotes NF-κB activation (Fig. 1).^267–270^ The constitutive activation of NF-κB is common in most types of B-NHLs.^269^ In DLBCL, NF-κB activity is upregulated in PMBCL and ABC-DLBCL but not in GCB-DLBCL.^173^ BCR-dependent NF-κB activation was the highest in the MCD subtype (based on the cooccurrence of MYD88 L265P and CD79B mutations) and BN2 subtype.^252^ In CLL, the NF-κB pathway is usually activated through BCR and TLRs.^271^ For mucosa-associated lymphoid tissue (MALT) lymphomas, intrinsic BCR activation is associated with an advanced stage.

The NF-κB pathway can be inhibited by directly or indirectly targeting NF-κB components. As a direct targeting agent, pevonedistat (TAK-924/MLN4924), a NEDD8-activating enzyme (NAE) inhibitor, suppresses NF-κB activity by blocking phospho-IκBα degradation.^272^ A phase 1 study (NCT03479268) of relapsed or refractory CLL and NHLs is ongoing. HSP90 is a component of the IKK complex and prevents the proteasomal degradation of IKKα and IKKβ.^270^ Two phase 1 trials (NCT00647764 and NCT00644072) of the HSP90 inhibitor SNX-5422 in patients with lymphomas were completed.

### Proteasome

UPP is a choreographed system that degrades misfolded proteins in all eukaryotic cells. It plays a role in the processes of cell apoptosis, cell-cycle progression, antigen presentation, and DNA repair.^273–276^ The first step of protein degradation is polyubiquitination, and the proteasome binds the polyubiquitin chain and mediates deubiquitination and then degrades the target proteins to oligopeptides less than 25 amino acids (Fig. 1).^277,278^ Inhibition of the pro-survival NF-κB pathway is the main antitumor mechanism of proteasome inhibitors in lymphoma.^279^

The targeted drugs and clinical trials related to the proteasome are listed in Table 4. Currently, three proteasome inhibitors (bortezomib, carfilzomib, and ixazomib) are approved for MM or MCL. Bortezomib, a reversible proteasome inhibitor, binds primarily with β5 and, to a lesser extent, with β2 and β1 of the 20S proteasome particle.^280^ A phase 2 trial (NCT00063713) of bortezomib in relapsed or refractory MCL reported an ORR of 31% (CR 8%).^281^ Another phase 2 trial (NCT00901147) of bortezomib and panobinostat showed an ORR of 43% (CR 22%) in relapsed or refractory PTCL patients.^282^ Bortezomib in combination with other agents, such as ibrutinib in MCL (NCT02356458), dexamethasone in CTCL (NCT03487133), and chemotherapeutic regimens, such as gemcitabine, dexamethasone, and cisplatin (GDP) in DLBCL (NCT02542111) and CHOP in T-NHLs (NCT00374699), are currently ongoing. A randomized phase 3 trial (NCT00722137) compared the efficacy of R-CHOP with bortezomib, rituximab, cyclophosphamide, doxorubicin, and prednisone (VR-CAP) in untreated MCL, showing an improved median PFS but increased hematologic toxicity.^283^ Moreover, a phase 2 trial of bortezomib, low-dose dexamethasone, and rituximab (NCT00981708) presented an ORR of 85% (CR 3%) in untreated WM.^284^ A phase 3 trial (NCT01788020) conducted in WM patients to evaluate bortezomib in combination with dexamethasone, cyclophosphamide, and rituximab is ongoing. Other proteasome inhibitors, including the irreversible carfilzomib and the reversible oral inhibitor ixazomib, have been studied in a variety of clinical trials. Trials of carfilzomib (NCT01336920) alone or in combination with other agents including vorinostat (NCT01276717), romidepsin (NCT03141203), umbralisib (NCT02867618), rituximab (NCT03269552), BR (NCT02187133), R-CHOP (NCT02073097) and R-ICE (NCT01959698) in relapsed or refractory lymphoma are ongoing. Phase 2 trials of ixazomib showed an ORR of 8.3% (CR 0%) in relapsed or refractory FL (NCT01939899) and an ORR of 67% in relapsed or refractory CTCL/PTCL (NCT02158975). Ixazomib in combination with rituximab (NCT02339922) or with ibrutinib (NCT03323151) is currently under evaluation in indolent B-NHLs and MCL. Phase 1/2 trials of ixazomib combined with romidepsin (NCT03547700) in refractory PTCL and with rituximab and lenalidomide as frontline therapy in high-risk indolent B-NHLs (NCT02898259) are ongoing.

**Table 4:** Targeted drugs and clinical trials related to the proteasome

Drug	Disease	Trial name	Phase	Status	ORR/CR	NCT#	Reference
*Proteasome inhibitor*							
*Bortezomib*	*A reversible proteasome inhibitor binding primarily with β5 and to a lesser extent, with β2 and β1 of the 20S proteasome particle*						
Bortezomib	Relapsed or refractory MCL	Bortezomib in subjects with relapsed or refractory mantle cell lymphoma	2	Completed	31%/8%	NCT00063713	^281^
Bortezomib, panobinostat	Relapsed or refractory PTCL	Study of bortezomib and panobinostat in treating patients with relapsed or refractory peripheral T-cell lymphoma	2	Completed	43%/22%	NCT00901147	^282^
Bortezomib, ibrutinib	MCL	Combination of ibrutinib and bortezomib to treat patients with mantle cell lymphoma	1/2	Recruiting	–	NCT02356458	–
Bortezomib, dexamethasone	Relapsed or refractory CTCL	Bortezomib plus dexamethasone therapy in patients with relapsed or refractory cutaneous T-cell lymphoma	2	Recruiting	–	NCT03487133	–
Bortezomib, GDP	Non-GCB DLBCL	A study of bortezomib plus GDP in the treatment of relapsed or refractory non-GCB DLBCL	2	Unknown	–	NCT02542111	–
Bortezomib, CHOP	Advanced aggressive T-NHLs/NKTCL	Bortezomib and CHOP in patients with advanced-stage aggressive T-cell or NK/T-cell lymphoma	1/2	Completed	NA	NCT00374699	–
VR-CAP vs. R-CHOP	Untreated MCL	Study of the combination of rituximab, cyclophosphamide, doxorubicin, bortezomib, and prednisone or rituximab, cyclophosphamide, doxorubicin, vincristine, and prednisone in patients with newly diagnosed mantle cell lymphoma	3	Completed	NA	NCT00722137	^283^
Bortezomib, dexamethasone, rituximab	Untreated WM	Bortezomib, low-dose dexamethasone, and rituximab in untreated Waldenström’s macroglobulinemia	2	Completed	85%/3%	NCT00981708	^284^
Bortezomib, dexamethasone, rituximab, and cyclophosphamide	WM	Efficacy of First-Line Dexamethasone, Rituximab, and Cyclophosphamide +/− Bortezomib for Patients With Waldenström’s Macroglobulinemia	3	Active, not recruiting	–	NCT01788020	–
*Carfilzomib*	*A second-generation irreversible proteasome inhibitor binding to the β5 subunit of the 20S proteasome particle*						
Carfilzomib	Relapsed or refractory T-NHLs	Carfilzomib in treating patients with relapsed or refractory T-cell lymphoma	1	Completed	NA	NCT01336920	–
Carfilzomib, vorinostat	Relapsed or refractory lymphoma	Study of carfilzomib and vorinostat for relapsed or refractory lymphoma	1	Completed	NA	NCT01276717	–
Carfilzomib, romidepsin	Relapsed or refractory PTCL	Evaluation of the combination of romidepsin and carfilzomib in relapsed or refractory peripheral T-cell lymphoma patients	1/2	Recruiting	–	NCT03141203	–
Carfilzomib, umbralisib	Relapsed or refractory lymphoma	Carfilzomib and umbralisib in treatment of relapsed or refractory lymphoma	1/2	Recruiting	–	NCT02867618	–
Carfilzomib, rituximab	WM/MZL	Carfilzomib with or without rituximab in the treatment of Waldenström’s macroglobulinemia or marginal zone lymphoma	2	Completed	NA	NCT03269552	–
Carfilzomib, bendamustine, rituximab	Relapsed or refractory NHLs	Carfilzomib with bendamustine and rituximab in patients with relapsed or refractory non-Hodgkin’s lymphoma	1	Recruiting	–	NCT02187133	–
Carfilzomib, R-CHOP	DLBCL	Carfilzomib, rituximab, and combination chemotherapy in treating patients with diffuse large B-cell lymphoma	1/2	Recruiting	–	NCT02073097	–
Carfilzomib, R-ICE	Relapsed or refractory DLBCL	Carfilzomib, rituximab, ifosfamide, carboplatin, and etoposide in treating patients with relapsed or refractory stage I-IV diffuse large B-cell lymphoma	1/2	Recruiting	–	NCT01959698	–
*Ixazomib*	*A reversible proteasome inhibitor binding to the β5 subunit of the 20S proteasome particle*						
Ixazomib	Relapsed or refractory FL	Phase 2 study of oral ixazomib in adult patients with relapsed or refractory follicular lymphoma	2	Completed	PSMB1 positice, 8.3%/0%; PSMB1 negative, 0%/0%	NCT01939899	–
Ixazomib	Relapsed or refractory CTCL/PTCL	Open-label, phase 2 study of ixazomib in patients with relapsed or refractory cutaneous and peripheral T-cell lymphoma	2	Completed	67%/NA	NCT02158975	–
Ixazomib, rituximab	Indolent B-NHLs	Ixazomib and rituximab in treating patients with indolent B-cell non-Hodgkin’s lymphoma	2	Recruiting	–	NCT02339922	–
Ixazomib, ibrutinib	Relapsed or refractory MCL	A study of ixazomib and ibrutinib in relapsed or refractory mantle cell lymphoma	1/2	Recruiting	–	NCT03323151	–
Ixazomib, romidepsin	Relapsed or refractory PTCL	Study of ixazomib and romidepsin in peripheral T-cell lymphoma	1/2	Recruiting	–	NCT03547700	–
lenalidomide, ixazomib, rituximab	High-risk indolent B-NHLs	Lenalidomide, ixazomib, and rituximab as frontline therapy for high-risk indolent B-cell lymphoma	1/2	Active, not recruiting	–	NCT02898259	–

NA: ORR or CR are not available on the clinicaltrials.gov or from the published article, although the trial has been completed

*GDP* gemcitabine, dexamethasone, and cisplatin, *CHOP* cyclophosphamide, doxorubicin, vincristine, prednisolone, *VR-CAP* bortezomib, rituximab, cyclophosphamide, doxorubicin, and prednisone, *R-CHOP* rituximab, cyclophosphamide, doxorubicin, vincristine, prednisolone, *R-ICE* rituximab, ifosfamide, carboplatin, etoposide, *PSMB1* proteasome subunit beta type-1

Directly targeting signaling pathways and off-target effects remain a major issue of signaling pathway inhibitors. For example, AEs of ibrutinib, such as atrial fibrillation and bleeding-related events, were connected with the irreversible targeting of ibrutinib on BTK signaling in cardiac myocytes and platelets.^285,286^ The off-target inhibition of kinases containing an analogous cysteine residue with BTK^C481^ may also be crucial to the side effects of ibrutinib.^287^ Moreover, drug resistance reduces the clinical efficacy, warranting further investigation on combined treatment and dual inhibitors. BTK^C481S^ in the ibrutinib binding site is associated with ibrutinib resistance^288,289^ but can be overcome in combination with venetoclax.^290^ mTOR inhibitors show limited long-term effectiveness due to feedback PI3K/AKT activation, while dual PI3K/mTOR inhibitors could be better alternatives.

## Epigenetic regulation and targeted therapy

**Fig. 2:**
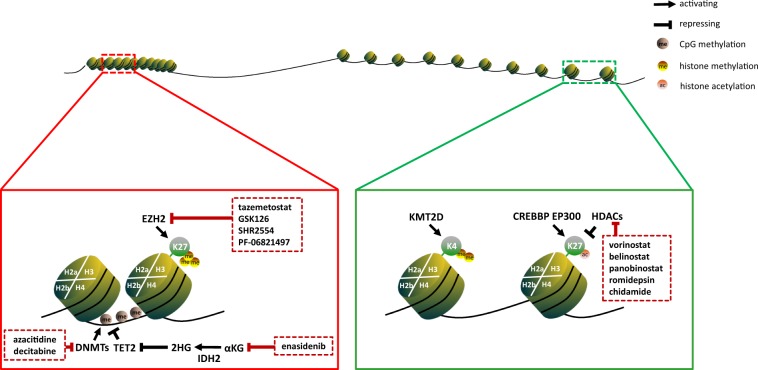
Epigenetic modifications in lymphoma cells

Epigenetic regulation mainly includes DNA methylation, histone acetylation and methylation. Histone acetylation and methylation regulate the chromatin state. In the active status, chromatin is accessible to transcription factors, which is represented by the enrichment of H3K27 acetylation and H3K4 methylation. In the repressive status, chromatin is compact and inaccessible to transcription factors, which is characterized by the enrichment of H3K36, H3K27 and H3K9 trimethylation (Fig. 2).^291^ Epigenetic dysregulation plays an important role in both B- and T-NHLs and represents potential therapeutic targets according to preclinical data and clinical trials.

### DNA methylation and targeted therapy

#### DNMT

The main type of DNA methylation observed in mammals is the methylation of CpG dinucleotides.^292^ DNA methyltransferases (DNMTs) mediate this process and induce transcriptional repression. DNMT1 maintains DNA methylation on hemimethylated CpG sites, whereas DNMT3A and DNMT3B are involved in DNA methylation on unmethylated CpG sites. In vitro, the molecular silencing of DNMT1 decreased the expression of cell-cycle genes, such as *CDK1*, *CCNA2*, and *E2F2*, in GCB-DLBCL-derived cell lines.^293^ Analysis of DLBCL patients reported the overexpression of DNMT1, DNMT3A, and DNMT3B in 48%, 13%, and 45% of patients, respectively.^294^ Moreover, *DNMT1* loss induced altered methylation levels and impaired tumor cell proliferation in mice with T-NHLs.^295^ Almost all T-NHL subtypes harbor mutations of *DNMT3A*.^296^

The targeted drugs and clinical trials related to epigenetic modifications are listed in Table 5. Azacitidine, a demethylating agent, inhibits DNMTs by incorporating into RNA and DNA through covalent bonding to DNMTs. A phase 1/2 trial (NCT01120834) showed that azacitidine in combination with vorinostat induced an ORR of 6.7% in patients with relapsed or refractory DLBCL. Azacitidine was also studied in combination with R-CHOP in a phase 1/2 trial (NCT01004991) that reported a CR rate of 91.7% in 12 untreated DLBCL patients. In addition, there are some other trials investigating azacitidine plus R-ICE (NCT03450343) or rituximab and GDP (R-GDP) (NCT03719989) in relapsed or refractory DLBCL and azacitidine with CHOP (NCT03542266) in untreated PTCL patients. Decitabine, a DNMT inhibitor, inhibits DNMTs by incorporating into DNA and reversing DNA methylation and transcriptional repression. A phase 1 trial of low-dose decitabine in NHL and CLL reported dose-limiting myelosuppression.^297^ Decitabine combined with R-CHOP is being studied in a phase 1/2 trial (NCT02951728) of untreated DLBCL patients with International Prognostic Index (IPI) >1. Moreover, there is a recruiting phase 4 trial (NCT03579082) exploring the efficacy and safety of decitabine, rituximab, with/without DHAP in relapsed or refractory DLBCL. A phase 3 randomized trial (NCT03553537) is comparing the efficacy and safety of decitabine plus CHOP (D-CHOP) vs CHOP alone in patients with untreated PTCL.

**Table 5:** Targeted drugs and clinical trials related to epigenetic modifications

Drug	Disease	Trial name	Phase	Status	ORR/CR	NCT#	Reference
*DNMT inhibitor*							
*Azacitidine*	*A DNMT inhibitor which is incorporated into RNA and, to a lesser extent, into DNA, and inhibits DNMTs*						
Azacitidine, vorinostat	Relapsed or refractory DLBCL	Study of azacitidine in combination with vorinostat in patients with relapsed or refractory diffuse large B-cell lymphoma	1/2	Completed	6.7%/NA	NCT01120834	–
Azacitidine, R-CHOP	Untreated DLBCL	Phase 1/2 trial of R-CHOP plus azacytidine in diffuse large B-cell lymphoma	1/2	Completed	NA/91.7%	NCT01004991	–
Azacitidine, R-ICE	Relapsed or refractory DLBCL	Oral azacitidine plus salvage chemotherapy in relapsed or refractory diffuse large B-cell lymphoma	1	Recruiting	–	NCT03450343	–
Azacitidine, R-GDP	Relapsed or refractory DLBCL	Azacitidine and R-GDP in patients with relapsed or refractory diffuse large B-cell lymphoma (EPIC)	2	Not yet recruiting	–	NCT03719989	–
Azacitidine, CHOP	Untreated PTCL	Azacitidine plus CHOP in patients with untreated peripheral T-cell lymphoma	2	Recruiting	–	NCT03542266	–
*Decitabine*	*A DNMT inhibitor which is incorporated into DNA and inhibits DNMTs through disrupting the interaction between DNA and DNMTs*						
Decitabine, R-CHOP	Untreated DLBCL with IPI > 1	Decitabine plus R-CHOP in diffuse large B-cell lymphoma	1/2	Active, not recruiting	–	NCT02951728	–
Decitabine combined with R±DHAP	Relapsed or refractory DLBCL	A clinical trial of decitabine in relapse or refractory diffuse large B-cell lymphoma	4	Recruiting	–	NCT03579082	–
Decitabine plus CHOP vs. CHOP	Untreated PTCL	Efficacy and safety of decitabine plus CHOP vs. CHOP in patients with untreated peripheral T-cell lymphoma	3	Not yet recruiting	–	NCT03553537	–
*IDH2 inhibitor*							
*Enasidenib*	*An IDH2 inhibitor*						
Enasidenib	AITL	Study of orally administered enasidenib in subjects with advanced solid tumors, including glioma, and with angioimmunoblastic T-cell lymphoma, with an *IDH2* mutation	1/2	Completed	NA	NCT02273739	–
*EZH2 inhibitor*							
*Tazemetostat*	*A selective inhibitor of EZH2*						
Tazemetostat	Relapsed or refractory B-NHLs	Open-label, multicenter, phase 1/2 study of tazemetostat as a single agent in subjects with advanced solid tumors or with B-cell lymphomas and tazemetostat in combination with prednisolone in subjects with DLBCL	1/2	Active, not recruiting	Phase 1 part, 38%/14%	NCT01897571	^324^
*SHR2554*	*A novel EZH2 inhibitor*						
SHR2554	Relapsed or refractory mature lymphoid neoplasms	A phase 1 study of SHR2554 in subjects with relapsed or refractory mature lymphoid neoplasms	1	Recruiting	–	NCT03603951	–
*PF-06821497*	*A novel EZH2 inhibitor*						
PF-06821497	Relapsed or refractory FL	PF-06821497 treatment of relapsed or refractory small cell lung cancer, castration resistant prostate cancer, and follicular lymphoma	1	Recruiting	–	NCT03460977	–
*HDAC inhibitor*							
*Vorinostat*	*A pan-HDAC inhibitor*						
Vorinostat	Relapsed or refractory DLBCL	An investigational drug study with vorinostat in relapsed diffuse large B-cell lymphoma (0683-013)	2	Completed	5.6%/5.6%	NCT00097929	^334^
Vorinostat	Relapsed or refractory FL/other subtypes of indolent B-NHLs/MCL	Vorinostat in treating patients with low-grade non-Hodgkin’s lymphoma	2	Completed	FL, 47%/23.5%; MZL, 22%/11%; MCL, 0%/0%	NCT00253630	^335^
Vorinostat	Relapsed and refractory CTCL	Oral vorinostat in advanced cutaneous T-cell lymphoma (0683-001)	2	Completed	29.7%/0%	NCT00091559	^336^
Vorinostat, rituximab	NHLs	Vorinostat and rituximab in treating patients with indolent non-Hodgkin’s lymphoma	2	Completed	FL, 50%/40.9%; MZL, 50%/50%; MCL, 33.3%/0%; LPL, 0%/0%	NCT00720876	^337^
Vorinostat, R-CHOP	Newly diagnosed advanced-stage DLBCL	Vorinostat, rituximab, and combination chemotherapy in treating patients with newly diagnosed stage II, stage III, or stage IV diffuse large B-cell lymphoma	1/2	Completed	81%/52%	NCT00972478	^338^
Vorinostat, R-ICE	Relapsed or refractory NHLs	Vorinostat, rituximab, ifosfamide, carboplatin, and etoposide in treating patients with relapsed or refractory lymphoma	1	Completed	70%/29.6%	NCT00601718	^339^
Vorinostat, CHOP	Relapsed or refractory PTCL	Phase I study of vorinostat in combination with standard CHOP in patients with newly diagnosed peripheral T-cell lymphoma	1	Completed	93%/93%	–	^340^
*Belinostat*	*A pan-HDAC inhibitor*						
Belinostat	Relapsed or refractory aggressive B-NHLs	Belinostat in treating patients with relapsed or refractory aggressive B-cell non-Hodgkin’s lymphoma	2	Completed	10.5%/0%	NCT00303953	^341^
Belinostat	Relapsed or refractory PTCL/CTCL	A phase II clinical trial of belinostat in patients with relapsed or refractory peripheral and cutaneous T-cell lymphomas (PXD101-CLN-6)	2	Completed	PTCL, 25%/8.3%; CTCL, 14%/10.3%	NCT00274651	^342^
Belinostat, carfilzomib	Relapsed or refractory NHLs	Carfilzomib plus belinostat in relapsed or refractory NHL	1	Completed	NA	NCT02142530	–
*Panobinostat*	*A pan-HDAC inhibitor*						
Panobinostat	Relapsed or refractory NHLs	Panobinostat in treating patients with relapsed or refractory non-Hodgkin’s lymphoma	2	Active, not recruiting	21%/NA	NCT01261247	–
Panobinostat, lenalidomide	Relapsed or refractory HL	A phase 2 trial of panobinostat and lenalidomide in patients with relapsed or refractory Hodgkin’s lymphoma	2	Completed	16.7%/8.3%	NCT01460940	–
Panobinostat, ICE vs. ICE	Relapsed or refractory HL	Panobinostat plus ifosfamide, carboplatin, and etoposide compared with ifosfamide, carboplatin, and etoposide for relapsed or refractory Hodgkin’s lymphoma	1/2	Completed	NA	NCT01169636	–
*Romidepsin*	*A selective HDAC I inhibitor*						
Romidepsin	Relapsed or refractory PTCL/CTCL	Romidepsin to treat patients with peripheral T-cell lymphoma and cutaneous T-Cell lymphoma	2	Completed	PTCL, 38%/18%; CTCL, 34%/5.6%	NCT00007345	^343^^,^^344^
Romidepsin, alisertib	Relapsed or refractory lymphoma	Alisertib and romidepsin in treating patients with relapsed or refractory B-cell or T-cell lymphoma	1	Completed	NA	NCT01897012	–
Romidepsin, duvelisib; bortezomib, duvelisib	Relapsed or refractory T-NHLs	Trial of duvelisib in combination with either romidepsin or bortezomib in relapsed or refractory T-cell lymphoma	1	Recruiting	–	NCT02783625	–
Romidepsin, lenalidomide	Relapsed or refractory NHLs/MM	Romidepsin in combination with lenalidomide in adults with relapsed or refractory lymphomas and myeloma	1/2	Active, not recruiting	–	NCT01755975	–
Romidepsin, pralatrexate	Lymphoid malignancies	Romidepsin plus pralatrexate in relapsed or refractory lymphoid malignancies	1/2	Recruiting	–	NCT01947140	–
Romidepsin, ixazomib	Relapsed or refractory PTCL	Study of ixazomib and romidepsin in peripheral T-cell lymphoma	1/2	Recruiting	–	NCT03547700	–
Romidepsin, carfilzomib	Relapsed or refractory PTCL	Evaluation of the combination of romidepsin and carfilzomib in relapsed or refractory peripheral T-cell lymphoma patients	1/2	Recruiting	–	NCT03141203	–
Romidepsin, pembrolizumab	Relapsed or refractory PTCL	Study of pembrolizumab in combination with romidepsin	1/2	Recruiting	–	NCT03278782	–
Romidepsin, azacitidine	Relapsed or refractory lymphoma	Romidepsin plus azacitidine in relapsed or refractory lymphoid malignancies	1/2	Active, not recruiting	–	NCT01998035	–
Romidepsin, gemcitabine	Relapsed or refractory PTCL	Phase 2 study of romidepsin plus gemcitabine in the relapsed or refractory peripheral T-cell lymphoma patients	2	Completed	30%/15%	NCT01822886	^345^
Romidepsin, ICE	Relapsed or refractory PTCL	Romidepsin, ifosfamide, carboplatin, and etoposide in treating participants with relapsed or refractory peripheral T-cell lymphoma	1	Completed	93%/80%	NCT01590732	^346^
Romidepsin, CHOP	Untreated PTCL	A study of escalating doses of romidepsin in association with CHOP in the treatment of peripheral T-cell lymphoma	1/2	Completed	68%/51%	NCT01280526	^347^
Romidepsin, CHOP vs. CHOP	Untreated PTCL	Efficacy and safety of romidepsin plus CHOP vs. CHOP in patients with untreated peripheral T-cell lymphoma	3	Active, not recruiting	–	NCT01796002	–
*Chidamide*	*A selective HDAC I inhibitor*						
Chidamide	Relapsed or refractory B-NHLs	Study of chidamide as a single-agent treatment for patients with relapse or refractory B-NHLs	2	Unknown	–	NCT03245905	–
Chidamide	Relapsed or refractory DLBCL/FL	Chidamide for patients with relapse or refractory diffuse large B-cell lymphoma and follicular lymphoma	2	Not yet recruiting	–	NCT03410004	–
Chidamide	Relapsed or refractpry PTCL	A multicenter, open-label, pivotal phase 2 study of chidamide in relapsed or refractory peripheral T-cell lymphoma	2	Completed	28%/14%	–	^348^
Chidamide, sintilimab	Relapsed or refractory ENKTCL	Chidamide in combination with sintilimab in relapsed or refractory ENKTCL	1/2	Recruiting	–	NCT03820596	–
Chidamide, DICE	Relapsed or refractory B-NHLs	Chidamide plus DICE regimen for patients with relapse or refractory B-cell non-Hodgkin’s lymphoma	2	Unknown	–	NCT03105596	–
Chidamide, VDDT	Relapsed or refractory DLBCL	Chidamide combined with VDDT regimen in the relapse or refractory diffuse large B-cell lymphoma	2	Recruiting	–	NCT02733380	–
Chidamide, R-GDP	Relapsed or refractory DLBCL	Chidamide combined with R-GDP in treating patients with relapsed or refractory diffuse large B-cell lymphoma	2	Recruiting	–	NCT03373019	–
Chidamide, R-CHOP	Relapsed or refractory DLBCL	Chidamide with R-CHOP regimen for DLBCL patients	2	Recruiting	–	NCT03201471	–
Chidamide, R-CHOP	Elderly DLBCL	Chidamide plus R-CHOP in elderly DLBCL	2	Completed	NA	NCT02753647	–
Chidamide, CHOP	Untreated PTCL	Clinical trial of chidamide combined with CHOP in peripheral T-cell lymphoma patients	1	Completed	NA	NCT02809573	–
Chidamide, CHOP	Untreated AITL	Study evaluating the safety and efficacy of chidamide plus CHOP in untreated subjects with angioimmunoblastic T-cell lymphoma	2	Recruiting	–	NCT03853044	–
Chidamide, CHOEP	PTCL	Chidamide combined with CHOEP regimen for peripheral T-cell lymphoma patients	2	Recruiting	–	NCT03617432	–
Chidamide, CPT	Relapsed or refractory PTCL	Chidamide combined with cyclophosphamide, prednisone, thalidomide in treatment of fragile patients with relapse or refractory peripheral T-cell lymphoma	2	Recruiting	–	NCT02879526	–
Chidamide, PET	AITL	Chidamide with prednisone, etoposide, and thalidomide regimen for angioimmunoblastic T-cell lymphoma	2	Unknown	–	NCT03273452	–
Chidamide, PECM	Relapsed or refractory PTCL	Chidamide combined with PECM in relapsed or refractory peripheral T-cell lymphoma	2	Recruiting	–	NCT03321890	–

NA: ORR or CR are not available on the clinicaltrials.gov or from the published article, although the trial has been completed

*R-CHOP* rituximab, cyclophosphamide, doxorubicin, vincristine, prednisolone, *R-ICE* rituximab, ifosfamide, carboplatin, etoposide, *R-GDP* rituximab, gemcitabine, dexamethasone, cisplatin, *CHOP* cyclophosphamide, doxorubicin, vincristine, prednisolone, *DICE* dexamethasone, ifosfamide, cisplatin, etoposide, *VDDT* vinorelbine, liposomal doxorubicin, dexamethasone and thalidomide, *CHOEP* cyclophosphamide, doxorubicin, vincristine, etoposide, prednisone, *CPT* cyclophosphamide, prednisone, thalidomide, *PET* prednisone, etoposide, thalidomide, *PECM* prednisone, cyclophosphamide, etoposide, methotrexate

#### TET2

TET2 mediates the oxidation process of 5-methylcytosine (5mC) in gene bodies to 5-hydroxymethylcytosine (5hmC), which plays an important role in transcriptional activation (Fig. 2).^298–301^ Experimentally, *TET2* deletion decreased DNA hydroxymethylation at enhancers and reduced the expression of a set of genes in GC B cells associated with GC exit and plasma cell differentiation.^302–307^ *TET2* was mutated in 12% of DLBCL patients, predominantly in the GCB subtype.^308^ *TET2* mutations occur more frequently in T-cell lymphomas, including 47% of AITL and 38% of PTCL-NOS.^309–311^ A retrospective study indicated that *TET2* mutations in PTCL were associated with advanced-stage disease and high-risk IPI.^310^ To date, there are no specific TET2 inhibitors in clinical application. However, the growth inhibition of *TET2*-knockdown DLBCL cells was observed after treatment with a histone deacetylase 3 (HDAC3) inhibitor in vitro.^312^ Clinically, AITL patients with *TET2* mutations were reported to have an objective response to azacitidine treatment.^313^

#### IDH2

The isocitrate dehydrogenase (IDH) family, including IDH1, IDH2, and IDH3, catalyzes the oxidative decarboxylation process that transduces isocitrate to α-ketoglutarate.^314^ Gain-of-function mutations of *IDH2*^*R172*^ result in the production of 2-hydroxyglutarate (2HG), which inhibits TET enzymes and histone-lysine demethylases and induces the epigenetic modification of DNA.^315–317^ Altered DNA methylation and downregulated Th1 cell differentiation-associated genes were observed in *IDH2*^*R172*^-mutant AITL.^316^ *IDH2*^*R172*^/*TET2* double mutations were found in AITL and correlated with increased follicular T-helper-associated gene expression.^316^ For targeted therapy, a phase 1/2 trial (NCT02273739) of enasidenib (also known as AG-221) in subjects with AITL that harbor *IDH2* mutations has been completed.

### Histone methylation and targeted therapy

#### EZH2

Enhancer of zeste homolog 2 (EZH2) functions as a histone methyltransferase and induces transcriptional repression via the trimethylation of H3K27. EZH2 in GC B-cells represses the expression of a set of genes involved in terminal differentiation, such as *PRDM1*, *IRF4*, and *XBP1*, as well as in the negative regulation of cell-cycle progression, such as *CDKN1A* and *CDKN1B*.^318–320^ Mutations of *EZH2* occur in 25% of FL and 21.7% of GCB-DLBCL but not in ABC-DLBCL.^319,321^ A strong association between *EZH2* mutations and the loss of MHC-I or MHC-II expression was found in DLBCL, especially in GCB-DLBCL.^322^ A higher level of H3K27me3 at promoters of *NLRC5* and *CIITA* (MHC-I and MHC-II transactivators) was also found in *EZH2-*mutant cells,^322^ indicating the underlying mechanisms of *EZH2* mutation on MHC expression. *EZH2* mutations also occur in T-NHLs. Tazemetostat, a selective inhibitor of EZH2, can effectively block H3K27 methylation and inhibit mutant lymphoma cells.^323^ The phase 1 part of a phase 1/2 study (NCT01897571) of tazemetostat in relapsed or refractory B-NHLs was completed and demonstrated acceptable safety and potential antitumor activity (ORR 38% and CR 14%).^324^ Additionally, phase 1 studies are assessing the novel EZH2 inhibitors SHR2554 and PF-06821497 in lymphoma (NCT03603951 and NCT03460977).

#### KMT2D

Histone-lysine N-methyltransferase 2D (KMT2D), also called MLL2, is a member of the SET1 family of histone methyltransferases and modulates transcription by H3K4 methylation. Integrative genomic analysis identified that KMT2D-targeted genes included *TNFAIP3*, *TNFRSF14*, and *SOCS3*, which suppress tumorigenesis, and genes involved in cell signaling pathways such as JAK-STAT and BCR.^325^ The incidence of inactivating mutations of *KMT2D* is observed in 72% of FL^319^ and 30% of DLBCL.^326^ *KMT2D* missense mutations lead to a significant reduction in H3K4 methylation in vitro.^327^ Recent studies in mice showed that the loss of *KMT2D* resulted in decreased H3K4 methylation and increased tumor development.^325,327^ Though there are no targeted agents for KMT2D, the histone deacetylase inhibitors (HDACis) romidepsin and chidamide showed the ability to restore H3K4me3 levels in *KMT2D* mutant cells in vitro.^328^ Chidamide combined with decitabine was observed to induce the apoptosis of Jurkat cells bearing *KMT2D* mutations in vitro and in vivo.^328^

### Histone acetylation

#### CREBBP/EP300

The balance between histone acetyltransferases (HATs, including CREBBP and EP300) and HDACs is critical to maintain a normal histone acetylation status in cells. CREBBP and EP300, as histone acetyltransferases, regulate gene transcription by catalyzing the acetylation of the lysine residues of histones. Inactivating mutations in *CREBBP* and *EP300* in GC B-cells decrease p53-mediated tumor suppression and enhance the proto-oncogenic activity of BCL-6.^329,330^ *CREBBP* mutation is also associated with reduced MHC-II expression, which is a key element in antigen presentation, thereby promoting tumor escape from the immune system.^331^ *CREBBP* and *EP300* mutations were found in 65% and 15% of FL, respectively. *CREBBP* is mutated in DLBCL, with a significantly higher incidence in the GCB subtype (32% in GCB-DLBCL vs. 13% in ABC-DLBCL). Mutations of *EP300* were observed in 10% of DLBCL.^329^ In PTCL-NOS, *CREBBP* and *EP300* are mutated in 4% and 8% of patients, respectively.^328^ In NKTCL, *EP300* is mutated in approximately 3.8% of patients.^243^

#### HDACs

HDACs are divided into four groups: HDAC I (HDAC 1, 2, 3, and 8), HDAC II (HDAC 4, 5, 6, 7, 9, and 10), HDAC III and HDAC IV.^332^ There are three types of HDACis under clinical development: pan-HDACis (vorinostat, belinostat, and panobinostat), selective HDACis (HDAC I inhibitors including romidepsin, chidamide, and entinostat; the HDAC6 inhibitor ricolinostat) and multipharmacological HDACis.^333^

Vorinostat (suberoylanilide hydroxamic acid, SAHA), the first HDACi approved by the FDA for treating CTCL, inhibits both HDAC I and HDAC II. A phase 2 trial (NCT00097929) of vorinostat in relapsed DLBCL presented an ORR of 5.6% (CR 5.6%), suggesting that vorinostat monotherapy has limited antitumor activity in relapsed DLBCL. Common AEs were grade 1/2 diarrhea, fatigue, nausea, anemia and vomiting, and grade ≥ 3 AEs including thrombocytopenia and asthenia occurred in 16.7% and 11.1% of the patients, respectively.^334^ Another phase 2 trial (NCT00253630) of vorinostat enrolled relapsed or refractory patients with B-NHLs and showed an ORR of 47% (CR 23.5%) in FL, 22% (CR 11%) in MZL, and no response in MCL. Grade ≥ 3 AEs were thrombocytopenia (39%), anemia (11%), leucopenia (11%), and fatigue (9%).^335^ Vorinostat in relapsed or refractory CTCL (NCT00091559) had an ORR of 29.7% (CR 0%).^336^ A phase 2 trial (NCT00720876) studied the efficacy and safety of vorinostat plus rituximab in NHLs, showing an ORR of 50% (CR 40.9%) in FL, 50% (CR 50%) in MZL, 33% (CR 0%) in MCL and no response in LPL.^337^ Vorinostat plus R-CHOP was explored in a phase 1/2 study (NCT00972478) and showed a tendency to improve R-CHOP in untreated advanced-stage DLBCL (ORR 81% and CR 52%); 38% febrile neutropenia and 19% sepsis were reported.^338^ Vorinostat in combination with R-ICE was applied in patients with relapsed or refractory NHLs (NCT00601718), and an ORR of 70% (CR 29.6%) was reported. Grade ≥ 3 AEs included febrile neutropenia (27%), infection (27%), and hypophosphatemia (27%) in patients treated at the maximum tolerated dose.^339^ A phase 1 trial investigated vorinostat in combination with standard CHOP in untreated PTCL patients and presented an ORR of 93% (CR 93%). Grade ≥ 3 AEs were neutropenia (50%), anemia (17%), and diarrhea (17%) in patients receiving 300 mg three times daily on days 2 to 3.^340^

Another pan-HDACi, belinostat (PXD101), was approved by the FDA to treat PTCL. A phase 2 trial (NCT00303953) of belinostat in relapsed or refractory aggressive B-NHLs reported an ORR of 10.5% (CR 0%).^341^ Another phase 2 trial (NCT00274651) explored belinostat in relapsed or refractory PTCL or CTCL with an ORR of 25% (CR 8.3%) in PTCL and an ORR of 14% (CR 10.3%) in CTCL. Treatment-related AEs were found in 77% of patients, including nausea (43%), vomiting (21%), infusion site pain (13%), and dizziness (11%).^342^ A trial of belinostat combined with carfilzomib in relapsed or refractory NHLs (NCT02142530) is ongoing.

Panobinostat, a pan-HDACi, showed an ORR of 21% in relapsed NHLs (NCT01261247). In relapsed or refractory HL, panobinostat in combination with lenalidomide (NCT01460940) had an ORR of 16.7% (CR 8.3%), while its effect in combination with ICE (NCT01169636) is currently under evaluation.

Romidepsin (FK228), a selective HDAC inhibitor, was approved by the FDA for treating CTCL. A phase 2 trial (NCT00007345) reported an ORR of 38% (CR 18%) in relapsed or refractory PTCL and an ORR of 34% (CR 5.6%) in relapsed or refractory CTCL. Common AEs included nausea, fatigue, transient thrombocytopenia and granulocytopenia.^343,344^ Trials of the combined treatment of romidepsin with other targeted agents, such as alisertib (NCT01897012), duvelisib (NCT02783625), lenalidomide (NCT01755975), pralatrexate (NCT01947140), ixazomib (NCT03547700), carfilzomib (NCT03141203), pembrolizumab (NCT03278782), and azacytidine (NCT01998035), in relapsed or refractory NHLs are ongoing. A phase 2 trial (NCT01822886) of romidepsin plus gemcitabine in relapsed or refractory PTCL showed an ORR of 30% (CR 15%).^345^ A phase 1 trial (NCT01590732) of romidepsin plus ICE in relapsed or refractory PTCL had an ORR of 93% (CR 80%), and the most common grade ≥ 3 AEs were thrombocytopenia (83%), anemia (50%), neutropenia (44%), fatigue (33%), nausea or vomiting (33%), infections (28%), dyspnea (17%), and transaminitis (11%).^346^ Of note, a phase 1/2 trial (NCT01280526) of romidepsin plus CHOP induced an ORR of 68% (CR 51%) in untreated PTCL.^347^ Thus, a randomized phase 3 trial (NCT01796002) of romidepsin plus CHOP vs CHOP in untreated PTCL is ongoing.

Chidamide, a selective HDAC I inhibitor, is being evaluated in relapsed or refractory B-NHLs (NCT03245905 and NCT03410004). In a phase 2 trial of relapsed or refractory PTCL, chidamide showed an ORR of 28% (CR 14%). Grade ≥ 3 AEs were thrombocytopenia (22%), leucopenia (13%), and neutropenia (11%).^348^ A trial of chidamide in combination with sintilimab is ongoing in relapsed or refractory NKTCL (NCT03820596). Phase 2 trials of chidamide in combination with chemotherapy, such as dexamethasone, ifosfamide, cisplatin, and etoposide (DICE) (NCT03105596), vinorelbine, liposomal doxorubicin, dexamethasone and thalidomide (VDDT) (NCT02733380), R-GDP (NCT03373019), and R-CHOP (NCT03201471) in relapsed or refractory B-NHLs, as well as R-CHOP (NCT02753647) in untreated elderly DLBCL patients, are ongoing. In T-NHLs, the efficacy of chidamide combined with CHOP (NCT02809573 and NCT03853044); cyclophosphamide, doxorubicin, vincristine, etoposide, and prednisone (CHOEP) (NCT03617432); cyclophosphamide, prednisone, and thalidomide (CPT) (NCT02879526); prednisone, etoposide, and thalidomide (PET) (NCT03273452); and prednisone, etoposide, cyclophosphamide, and methotrexate (PECM) (NCT03321890) are under evaluation.

Although epigenetic alterations show clinical significance, modulators specifically targeting these alterations remain to be developed. Demethylation agents and HDACis have presented clinical efficacy in many lymphoma subtypes. However, the exact mechanisms of action remain unclear, and biomarkers to predict clinical effects need to be further explored. Moreover, monotherapy with epigenetic agents may have limited efficacy in lymphoma in early phase studies. Trials in combination with chemotherapy or other small molecules have demonstrated potent efficacy and acceptable safety and warrant further investigation.

## Tumor microenvironment and checkpoint-related targeted therapy

In addition to tumor cells themselves, the tumor microenvironment plays an important role in lymphoma progression. Immunotherapeutic agents can effectively activate the immune system, leading to tumor regression, and have improved clinical outcomes in lymphoma patients.^349–352^ In addition, checkpoint inhibitors combined with CAR-T therapy, epigenetic modulators, radiotherapy, and BTK inhibitors have shown striking efficacy in refractory lymphoma.^353–355^

### PD-1/PD-L1

Programmed cell death-1 (PD-1, also known as CD279) is a member of the immunoglobulin superfamily and functions as an important immune checkpoint that suppresses excessive immune responses.^6^ PD-1 is mainly expressed on activated T cells and a small number of B cells, NK cells, activated monocytes, and dendritic cells but is not expressed on naïve T cells. The persistent stimulation of PD-1 on T cells can lead to T-cell exhaustion.^356,357^ The ligands of PD-1 include PD-L1 (also known as B7-H1, CD274) and PD-L2 (also known as B7-DC, CD273).^358,359^ PD-L1 is expressed on B cells, T cells, dendritic cells, and macrophages. PD-L2 is expressed mainly on dendritic cells, macrophages, mast cells, and certain B cells in response to IL-4 and IFN.^358,359^ In addition to those immune cells, PD-L1 is expressed on tumor cells and protects them from immune surveillance; a high level of PD-L1 on tumor cells is associated with poor prognosis in patients.^360–363^ Therefore, PD-1/PD-L1 pathway blockade can promote T-cell activation and cytokine production and preserve the antitumor capacity of T cells in the treatment of lymphomas.^364^

PD-1 is overexpressed in the tumor-infiltrating lymphocytes (TILs) of HL,^365^ and 94–100% of refractory or relapsed HL cases are positive for PD-L1.^353,366^ The 9p24.1 amplification is frequently detected in HL, resulting in increased PD-L1 and PD-L2 expression on Hodgkin and Reed–Sternberg (HRS) cells.^367^ Moreover, the amplified 9p24.1 region contains the *JAK2* locus, further enhancing PD-L1 expression in HRS cells.^367^ In FL, though PD-1 expression on TILs is abundant, PD-L1 expression on lymphoma cells is low (0–5%).^368–375^ In DLBCL, the positive rate of PD-1 was 39.5–68.6%,^376–380^ and the positive rate of PD-L1 was 24–75%.^375,380–382^ Moreover, the number of PD-1^+^ TILs is higher in the GCB subtype, and patients with PD-L1^+^ tumor cells have inferior OS compared to those with PD-L1^-^ tumor cells.^379^ Soluble PD-L1 (sPD-L1), independent of IPI, has been reported to be an adverse prognostic factor for DLBCL. Similar to PD-1, sPD-L1 is elevated in DLBCL patients at diagnosis and returns to normal when patients achieve CR. Thus, sPD-L1 is an effective predictor of DLBCL.^382^ In PTCL, PD-1 is positive in 70% and 61% of AITL and PTCL-NOS, respectively, and PD-1 is rarely detected in ALCL. PD-L1 is expressed in 46% of ALK^+^ ALCL and in 46% of ALK^-^ ALCL. In contrast, there is no PD-L1 expression in AITL and PTCL-NOS.^383^

The targeted drugs and clinical trials related to PD-1 are shown in Table 6. Nivolumab and pembrolizumab were approved by the FDA to treat relapsed or refractory HL.^366,377,384,385^ In a phase 1 trial (NCT01592370) of nivolumab in relapsed or refractory HL, the ORR was 87% (CR 17%), and the 24-week PFS was 86%.^349^ In a phase 2 trial (NCT02181738), the efficacy of nivolumab was evaluated in relapsed or refractory HL. At a median follow-up of 8.9 months, the ORR was 66.3% (CR 9%), and the 6-month PFS and OS were 77% and 99%, respectively.^350^ In relapsed or refractory NHLs, a phase 1 trial of nivolumab (NCT01592370) showed an ORR of 40% (CR 10%) in FL, 36% (CR 18%) in DLBCL, and 17% (CR 0%) in T-NHLs.^386^ Another phase 2 trial (NCT02038933) of nivolumab in ASCT-failed DLBCL showed an ORR of 10.3% (CR 3.4%). Nivolumab in relapsed or refractory ALK^+^ ALCL (NCT03703050) and PTCL (NCT03075553) is currently under clinical evaluation in phase 2 trials. In relapsed or refractory PCNSL and testicular lymphoma (NCT02857426), nivolumab showed an ORR of 100% (CR 80%).^351^ In addition, nivolumab combined with BV (NCT02572167) in relapsed or refractory HL had a reported ORR of 82% (CR 16%),^387^ and this combination in NHLs (NCT02581631) is ongoing. Nivolumab combinations with other targeted agents such as lenalidomide in relapsed or refractory lymphoma (NCT03015896), rituximab in FL (NCT03245021), cabiralizumab in PTCL (NCT03927105), and in combination with chemotherapy, such as rituximab, gemcitabine, and oxaliplatin (R-GemOx) in elderly lymphoma patients (NCT03366272), R-CHOP (NCT03704714), and rituximab, dose-adjusted etoposide, prednisone, vincristine, cyclophosphamide, and doxorubicin (DA-R-EPOCH) (NCT03749018) in aggressive NHLs are ongoing.

**Table 6:** Targeted drugs and clinical trials related to PD-1

Drug	Disease	Trial name	Phase	Status	ORR/CR	NCT#	Reference
*Nivolumab*	*A human monoclonal antibody that blocks the interaction between PD-1 and its ligands, PD-L1 and PD-L2*						
Nivolumab	Relapsed or refractory HL	PD-1 blockade with nivolumab in relapsed or refractory Hodgkin’s lymphoma	1	Completed	87%/17%	NCT01592370	^349^
Nivolumab	Relapsed or refractory HL	Nivolumab for classical Hodgkin’s lymphoma after failure of both autologous stem-cell transplantation and brentuximab vedotin: a multicenter, multicohort, single-arm phase 2 trial	2	Completed	66.3%/9%	NCT02181738	^350^
Nivolumab	Relapsed or refractory NHLs/MM	Nivolumab in patients with relapsed or refractory hematologic malignancy: preliminary results of a phase 1 study	1	Completed	FL, 40%/10%; DLBCL, 36%/18%; T-NHLs, 17%/0%	NCT01592370	^386^
Nivolumab	Relapsed or refractory DLBCL (failed or not eligible for ASCT)	Study of nivolumab in patients with relapsed or refractory diffuse large B-cell lymphoma that have either failed or are not eligible for autologous stem cell transplant	2	Completed	ASCT-failed, 10.3%/2.9%; ASCT ineligible, 3.4%/0%	NCT02038933	–
Nivolumab	Relapsed or refractory ALK^+^ ALCL	Phase 2 trial of nivolumab for pediatric and adult relapsed or refractory ALK^+^ anaplastic large cell lymphoma, for evaluation of response in patients with progressive disease (cohort 1) or as consolidative immunotherapy in patients in complete remission after relapse (cohort 2)	2	Recruiting	–	NCT03703050	–
Nivolumab	Relapsed or refractory PTCL	Nivolumab in treating patients with relapsed or refractory peripheral T-cell lymphoma	2	Active, not recruiting	–	NCT03075553	–
Nivolumab	Relapsed or refractory PCNSL/primary testicular lymphoma	PD-1 blockade with nivolumab in relapsed or refractory primary central nervous system and testicular lymphoma	2	Completed	100%/80%	NCT02857426	^351^
Nivolumab, BV	Relapsed or refractory HL	Interim results of brentuximab vedotin in combination with nivolumab in patients with relapsed or refractory Hodgkin’s lymphoma	1/2	Completed	82%/61%	NCT02572167	^387^
Nivolumab, BV	NHLs	An investigational immunotherapy effectiveness and safety study of nivolumab in combination with brentuximab vedotin to treat non-Hodgkin’s lymphomas	1/2	Active, not recruiting	–	NCT02581631	–
Nivolumab, lenalidomide	Relapsed or refractory NHLs/HL	Nivolumab and lenalidomide in treating patients with relapsed or refractory non-Hodgkin’s or Hodgkin’s lymphoma	1/2	Recruiting	–	NCT03015896	–
Nivolumab, rituximab	FL	Nivolumab plus rituximab in first-line follicular lymphoma grade 1-3A	1	Recruiting	–	NCT03245021	–
Nivolumab, cabiralizumab	PTCL	Nivolumab and the antagonistic CSF-1R monoclonal antibody cabiralizumab in patients with relapsed or refractory peripheral T-cell lymphoma	2	Recruiting	–	NCT03927105	–
Nivolumab, rituximab, gemcitabine, oxaliplatin	NHLs (elderly patients)	Nivolumab with gemcitabine, oxaliplatin, rituximab in relapsed or refractory elderly lymphoma patients	2/3	Recruiting	–	NCT03366272	–
Nivolumab, R-CHOP	Aggressive NHLs	Nivolumab and combination chemotherapy in treating participants with diffuse large B-cell lymphoma	1/2	Recruiting	–	NCT03704714	–
Nivolumab, DA-R-EPOCH	Aggressive NHLs	Nivolumab with DA-REPOCH chemotherapy regimen in treating patients with aggressive B-cell non-Hodgkin’s lymphoma	2	Recruiting	–	NCT03749018	–
*Pembrolizumab*	*A humanized monoclonal antibody that blocks the interaction between PD-1 and its ligands, PD-L1 and PD-L2*						
Pembrolizumab	Relapsed or refractory HL	PD-1 blockade with pembrolizumab in patients with classical Hodgkin’s lymphoma after brentuximab vedotin failure	1	Completed	65%/16%	NCT01953692	^353^
Pembrolizumab	Relapsed or refractory HL	Phase 2 study of the efficacy and safety of pembrolizumab for relapsed or refractory classic Hodgkin’s lymphoma	2	Completed	69%/22.4%	NCT02453594	^366^
Pembrolizumab	Transformed DLBCL/relapsed or refractory CLL	Pembrolizumab in patients with CLL and Richter transformation or with relapsed CLL	2	Completed	transformed DLBCL, 41%/11%; CLL, 0%/0%	NCT02332980	^388^
Pembrolizumab	Relapsed or refractory PMBCL	Safety and tolerability of pembrolizumab in patients with relapsed or refractory primary mediastinal large B-cell lymphoma	1	Completed	41%/11.8%	NCT01953692	^389^
Pembrolizumab	Relapsed or refractory GZL/extranodal DLBCL	Pembrolizumab in relapsed or refractory gray-zone lymphoma, primary central nervous system lymphoma, and other extranodal diffuse large B-cell lymphomas	2	Recruiting	–	NCT03255018	–
Pembrolizumab	Untreated B-NHLs	Pembrolizumab in untreated B-cell non-Hodgkin’s lymphoproliferative diseases	2	Recruiting	–	NCT03498612	–
Pembrolizumab	Relapsed or refractory stage IB-IVB MF/SS	A phase 2 study of pembrolizumab for the treatment of relapsed or refractory mycosis fungoides/Sézary syndrome	2	Completed	37.5%/8.3%	NCT02243579	–
Pembrolizumab	Stage IB-IV MF	Pembrolizumab in treating patients with stage IB-IV mycosis fungoides	2	Recruiting	–	NCT03695471	–
Pembrolizumab	Early stage NKTCL, nasal type	Study of pembrolizumab in patients with early stage NK/T-Cell lymphoma, nasal type	2	Recruiting	–	NCT03728972	–
Pembrolizumab, umbralisib	Relapsed or refractory B-NHLs/CLL	Combination of pembrolizumab with umbralisib in patients with relapsed or refractory CLL and B-NHLs	1	Recruiting	–	NCT03283137	–
Pembrolizumab, lenalidomide	Relapsed NHLs/HL	Efficacy and safety study of combination of pembrolizumab and lenalidomide, in patients with relapsed non-Hodgkin’s and Hodgkin’s lymphoma	1/2	Active, not recruiting	–	NCT02875067	–
Pembrolizumab, mogamulizumab	Relapsed or refractory NHLs/HL	Pembrolizumab and mogamulizumab in treating patients with relapsed or refractory lymphomas	1/2	Recruiting	–	NCT03309878	–
Pembrolizumab, rituximab	Relapsed or refractory DLBCL/FL	Pembrolizumab and rituximab in treating patients with relapsed or refractory diffuse large B-cell lymphoma or follicular lymphoma	2	Recruiting	–	NCT03401853	–
Pralatrexate	Relapsed or refractory mature T- and NK-cell NHLs/MF	Pembrolizumab and pralatrexate in treating participants with relapsed or refractory peripheral T-cell lymphoma	1/2	Recruiting	–	NCT03598998	–
Pembrolizumab, tisagenlecleucel	Relapsed or refractory DLBCL	Study of pembrolizumab in combination with tisagenlecleucel in relapsed or refractory diffuse large B-cell lymphoma patients	1	Recruiting	–	NCT03630159	–
Pembrolizumab, EBRT	Relapsed or refractory NHLs	Pembrolizumab and external beam radiation therapy in treating participants with relapsed or refractory non-Hodgkin lymphomas	2	Recruiting	–	NCT03210662	–
Pembrolizumab, radiotherapy	Relapsed or refractory MF/SS	A trial assessing the effect of pembrolizumab combined with radiotherapy in patients with relapsed or refractory, specified stages of cutaneous T-cell lymphoma mycosis fungoides/Sézary syndrome (PORT)	2	Recruiting	–	NCT03385226	–

NA: ORR or CR are not available on the clinicaltrials.gov or from the published article although the trial has been completed

*BV* brentuximab vedotin, *R-CHOP* rituximab, cyclophosphamide, doxorubicin, vincristine, prednisolone, *DA-R-EPOCH* rituximab, dose-adjusted EPOCH, *GZL* gray-zone lymphoma, *EBRT* external beam radiotherapy

Pembrolizumab, a humanized mAb of PD-1, showed an ORR of 65% (CR 16%) in a phase 1 trial (KEYNOTE-013, NCT01953692) of relapsed or refractory HL^353^ and an ORR of 69% (CR 22.4%) in a phase 2 trial (KEYNOTE-087, NCT02453594).^366^ Pembrolizumab induced an ORR of 41% (CR 11%) in transformed DLBCL and showed no response in relapsed or refractory CLL in a phase 2 trial (NCT02332980).^388^ In a phase 1 trial (NCT01953692), pembrolizumab was evaluated in relapsed or refractory PMBCL, and the ORR was 41% (CR 11.8%).^389^ Trials of pembrolizumab in relapsed or refractory gray-zone lymphoma and PCNSL (NCT03255018) and in untreated B-NHLs (NCT03498612) are ongoing. In a study of relapsed or refractory NKTCL patients who failed asparaginase treatment or ASCT, pembrolizumab presented an ORR of 100% (CR 71.4%).^352^ In a phase 2 trial (NCT02243579) of pembrolizumab in advanced relapsed or refractory MF and SS, the ORR was 37.5% (CR 8.3%). Thus, trials of pembrolizumab in MF (NCT03695471) and NKTCL (NCT03728972) are ongoing. Pembrolizumab in combination with other targeted agents, such as umbralisib (NCT03283137), lenalidomide (NCT02875067), mogamulizumab (NCT03309878), rituximab (NCT03401853), pralatrexate (NCT03598998), CAR-T (tisagenlecleucel, NCT03630159), and radiation (NCT03210662 and NCT03385226), are under evaluation.

### CTLA-4

CTLA-4, a member of the immunoglobulin family receptors, together with CD28, are homologous receptors of CD4^+^ and CD8^+^ T cells. Both receptors share a pair of ligands (CD80/CD86) expressed on the surface of antigen-presenting cells (APCs). In contrast with CD28, the signal of CTLA-4 suppresses the activation of T cells, and the affinity of CTLA-4 and CD80 is higher than that of CD28 and CD80. In addition to APCs, CTLA-4 is also present in resting T cells in the form of intracellular vesicles and expressed on the cell membrane surface when T cells are activated.^390^ The CTLA4-CD86 protein recruits and activates Tyk2, leading to STAT3 activation and the expression of genes involved in immune suppression and tumor growth. Although the CTLA-4 antibody ipilimumab^391^ has become a first-line therapy in metastatic melanoma,^392,393^ the application of the CTLA-4 antibody still needs to be explored in hematological malignancies.^394^ The phase 1 part of a phase 1/2 trial (NCT00089076) of ipilimumab in relapsed or refractory B-NHLs induced an ORR of 11.1% (CR 5.6%).^395^ Trials of ipilimumab combined with other agents, such as nivolumab (NCT02408861) in HIV-associated HL, are ongoing. A trial of tremelimumab, another CTLA-4 mAb, in combination with durvalumab in relapsed or refractory DLBCL (NCT02549651) was completed.

### CD47/SIRPα

CD47 is a new immune checkpoint that is expressed in normal cells and upregulated in various tumors.^396–398^ Its ligand SIRPα is expressed on myeloid cells (monocytes, macrophages, and myeloid dendritic cells). CD47/SIRPα mainly regulates innate immune cell activity and sends out a “do not eat me” signal to escape the attack of innate immune cells.^399^ MYC can upregulate the expression of the *CD47* gene by binding to the promoter of *CD47*. The downregulation of *MYC* gene expression in a murine model led to decreased CD47 expression.^400^ CD47 is upregulated in various NHLs (DLBCL, MCL, FL, and CLL) and is associated with poor clinical outcomes in patients.^401^ Targeting CD47 can reduce liver and central nervous system metastasis in Raji-engrafted mice,^402^ suggesting the association of CD47 with the extranodal metastasis of lymphoma cells. TTI-621 (an anti-CD47 antibody) enhances macrophage-mediated phagocytosis and can effectively control B-NHL growth in xenograft murine models. Another anti-CD47 antibody, Hu5F9-G4, combined with rituximab, is effective in the treatment of NHLs. In the phase 1 part of a phase 1/2 trial (NCT02953509), 22 relapsed or refractory DLBCL and FL patients were enrolled. The ORRs and CR rates were 40% and 33% in DLBCL and 71% and 43% in FL, respectively. The most common AEs were anemia and infusion reactions.^403^

### OX40/OX40L

OX40 is a member of the TNFR superfamily. Under physiological conditions, it is mainly expressed in activated T cells and is more abundant in CD4^+^ T cells than in CD8^+^ T cells. OX40L, the ligand of OX40, is a type II transmembrane protein and is expressed in a variety of APCs (B-cells, dendritic cells, and macrophages), activated T cells, vascular endothelial cells and mast cells.^404–406^ The OX40L-OX40 signaling pathway is the basis for effector T-cell proliferation and memory T-cell development. However, the OX40L-OX40 axis can promote immune escape and tumor growth.

Experimentally, an OX40 agonist showed antitumor activity in combination with other drugs. Intratumoral injection of anti-CTLA-4 and OX40 agonists depleted tumor-infiltrating Tregs in murine lymphoma models.^407^ A phase 1 clinical study (NCT03636503) of PF-04518600 (the OX40 agonist) in combination with utomilumab (4-1BB agonist) and rituximab or in combination with avelumab (anti-PD-L1) and rituximab is ongoing in aggressive B-NHLs.

### Other immune checkpoint molecules

T-cell immunoglobulin and ITIM domain (TIGIT) is a coinhibitory receptor that is expressed on NK cells and different types of T cells, including effector and memory T cells and Tregs.^408–410^ The ligands of TIGIT, CD155 (PVR) and CD112 (PVRL2, nectin-2) are expressed on APCs, T cells, and tumor cells.^411,412^ In NHL, TIGIT and PD-1 are frequently coexpressed on TILs. Approximately 78–83% of CD8^+^ and 69–70% of CD4^+^ T effector memory cells (TEMs) are simultaneously positive for these two inhibitory molecules, and these TEMs have limited capability for IL-2, IFN-γ, and TNF-α secretion.^413^ In FL, TIGIT is mainly expressed by CD8^+^ effector and memory T cells and is related to advanced disease stage.^414^ TIM-3 inhibits Th1 cell responses,^415^ and its antibodies have been found to potently enhance antitumor immunity.^416^ An increased number of TIM-3^+^ T cells is related to the unfavorable prognosis of FL patients.^414^ TIM-3 is preferentially expressed on the microvascular endothelial cells of lymphoma, suppresses the activation of CD4^+^ T lymphocytes and facilitates the progression of lymphoma by mediating immune evasion.^417^

Indoleamine 2,3-dioxygenase (IDO), a known immune suppressor, plays a role in human mesenchymal stromal cells (MSCs) to regulate immunity in the tumor microenvironment. IDO^+^ MSCs can inhibit T-cell proliferation in vitro. In a lymphoma murine model, IDO^+^ MSCs could enhance tumor growth, which could be reversed by the IDO inhibitor D-1-methyl-tryptophan (D1-MT).^418^ Since MSCs secrete IDO to further suppress T-cell immune responses, umbilical cord-derived MSCs genetically secrete TandAb (a tetravalent bispecific antibody with two CD3 and two CD19 binding sites). In vitro, TandAb can induce the specific lysis of CD19^+^ cell lines in the presence of T cells, and an IDO inhibitor could enhance the cytotoxicity of T cells triggered by MSC-TandAb.^419^ Clinical studies of IDO inhibitors in lymphomas are still lacking. V-domain immunoglobulin suppressor of T-cell activation (VISTA) is another checkpoint molecule that has a strong inhibitory influence on T cells.^420^ VISTA is constitutively expressed in CD11b^high^ myeloid cells and is expressed at a low level on T cells and Foxp3^+^CD4^+^ Treg cells.^421^ In animal models with solid tumors, myeloid-derived suppressor cells (MDSCs) infiltrating tumors were found to highly express VISTA compared to peripheral blood cells.^422,423^ In a murine model of squamous cancer, anti-VISTA monotherapy increased the infiltration and activation of T cells. A clinical trial (NCT02812875) evaluating the efficacy and safety of CA-107 (targeting PD-L1, PD-L2, and VISTA) for the treatment of lymphoma is ongoing.

## Adoptive T/NK-cell therapy

Adoptive T-cell transfer is an emerging immunotherapy in a variety of tumors, particularly CAR-T therapy. In 2017, the FDA approved tisagenlecleucel (a CD19-specific 4-1BB-CAR construct) for the treatment of relapsed or refractory B-ALL, and in 2018, the FDA approved axicabtagene ciloleucel (a CD19-specific CD28-CAR construct) for the treatment of relapsed or refractory DLBCL. Another CD19 CAR-T cell line, lisocabtagene maraleucel (a CD19-specific 4-1BB-CAR construct), is also undergoing evaluation.

### CAR-T therapy in lymphoma

In a single-arm, multicenter clinical trial (NCT02348216) for relapsed or refractory DLBCL, transformed FL, and PMBCL, axicabtagene ciloleucel had an ORR of 83% (CR 58%) and median PFS of 5.9 months.^424^ In another clinical trial (NCT02030834) of relapsed or refractory B-NHLs, tisagenlecleucel induced an ORR of 64.3% (CR 57.1%). Moreover, all CR patients were still in remission at 6 months.^425^ In a phase 2 trial (NCT02445248) of tisagenlecleucel in relapsed or refractory DLBCL, the ORR was 52% (CR 40%), with a 1-year RFS of 65%.^426^

In addition to axicabtagene ciloleucel and tisagenlecleucel, lisocabtagene maraleucel was tested in relapsed or refractory DLBCL, PMBCL, FL, and MCL (TRANSCEND, NCT02631044) and showed a CR rate of 80% in high-grade B-cell lymphoma (double/triple hit) and DLBCL.^427,428^ A phase 1 dose-escalation study (NCT03355859) of anti-CD19 JWCAR029 was conducted in refractory B-NHLs, and the ORR was 100%, with 6 of 9 (66.7%) evaluable patients achieving CR. In this study, core needle biopsy was performed on tumor samples on day 11 after CAR-T cell infusion. Further RNA sequencing of these tumor samples identified gene expression signatures differentially enriched in complete and partial remission patients. Increased tumor-associated macrophage infiltration was negatively associated with remission status.^429^

In addition to studies targeting CD19 CAR-T cells, studies on CD20, CD22, and CD30 CAR-T cell therapy have also been carried out. In a phase 2 study (NCT01735604) of anti-CD20 CAR-T therapy, the ORR was 81.8% (CR 54.5%).^430^ In a phase 1 trial (NCT02315612), anti-CD22 CAR-T cells were evaluated in patients with B-cell malignancies resistant to CD19 CAR-T cells and showed a CR rate of 73%, with a median remission duration of 6 months.^431^ A phase 1 trial (NCT01306146) of anti-CD30 CAR-T cells showed a CR rate of 28.6% in relapsed HL and a CR rate of 50% in ALCL.^432^

Anti-CD4 CAR-T cells could control the growth of tumors in a xenograft murine model of ALCL.^433^ However, this therapy also faces the challenge of CAR-T cells sharing antigens with normal T cells and can recognize and kill three types of cells: tumor T cells, normal T cells, and CAR-T cells. This problem can lead to the “auto-phase killing” of CAR-T cells, while CAR-T cells targeting normal T cells may lead to severe infection in patients.^434^ Therefore, reducing the side effects of CAR-T cells in T-NHLs has become the focus of research. Moreover, using CRISPR/Cas9 gene-editing technology, generating CAR-T cells (also known as UCART7) that lack CD7 and TCR alpha-chain expression could target CD7^+^ T-cell malignancies and reduce mutual attacks between CAR-T cells.^435^

Although CAR-T therapy has been successful in the treatment of hematological malignancies, there are still patients who do not respond to the treatment, as well as some patients presenting signs of AEs such as severe cytokine release syndrome (CRS), infection, and neurotoxicity. Therefore, identifying patients who may respond to CAR-T therapy and patients who may have serious side effects during treatment has become a research hotspot.

### CAR-NK therapy

With the continuous development of CAR-T therapy, CAR-NK cells have also become a focus of attention. NK cells are cytotoxic immune cells that form a small fraction of normal lymphocytes and can trigger the innate immune response against tumor cells and virus-infected cells.^436^ Studies have shown that NK cells have a nonnegligible role in tumor monitoring, and loss of NK cells leads to tumor progression.^437–439^ Because NK and T cells are functionally similar, NK cells can also be used to attack tumors. Many researchers hope that CAR-NK cells can achieve results in tumor treatment similar to CAR-T cells. Compared with T cells, NK cells kill tumor cells in a nonantigen-dependent manner. Moreover, NK cells express CD56 and CD7 but lack the expression of CD3, TCR, and CD5.^440^ When used in the treatment of T-NHLs, the fratricide of CAR-NK cells was reduced.^441^

Anti-CD19 cord blood (CB)-derived NK cells were evaluated in a xenograft lymphoma murine model and significantly prolonged the survival of mice.^442^ In another study, anti-CD5 CAR-NKs had potent antitumor activity against a variety of T-NHLs and primary tumor cells in vitro and in a murine model.^443^ Clinical trials (NCT03383965, NCT029170083 and NCT03049449) of CAR-NK cells targeting CD19, CD20, and CD22 have begun for the treatment of B-NHLs. In addition, a phase 1 trial (NCT02742727) of anti-CD7 CAR-NK for the treatment of T-cell malignancies is ongoing.

## Specific oncogenes and proteins related to targeted therapy

Specific oncogenes, such as MYC, BCL-2, and BCL-6, converge proliferation, differentiation, and anti-apoptotic signaling in lymphoma cells and play critical roles in lymphomas. Moreover, lymphomas that have a concomitant translocation of *MYC* and *BCL-2* or *BCL-6* represent high-grade B-cell lymphoma and are resistant to conventional R-CHOP chemotherapy. The tumor suppressor gene p53 is involved in the process of DNA repair, and the depletion or mutation of *p53* promotes lymphoma progression and drug resistance. The t(2;5)(p23;q35) translocation results in the NPM1/ALK fusion protein and then activates the downstream oncogenic transcription factor STAT3, enhancing lymphoma cell proliferation and growth. Thus, these specific oncogenes are greatly involved in lymphoma genesis and progression, and targeting these genes and their downstream pathways might retard tumor progression and improve patient survival.

### MYC

*MYC* is a family of three proto-oncogenes that function as important regulators of cell proliferation, growth, differentiation, and apoptosis. They encode the related transcription factors MYC, MYCN, and MYCL, also known as c-MYC, N-MYC, and L-MYC, respectively.^444^ The *Ig-MYC* translocation is the most common type of *MYC* alteration and can cause MYC overexpression.^445^ MYC is expressed at the pro-B and pre-B-cell stages and in a minority of GC B-cells.^446–448^ Additionally, MYC is frequently overexpressed in lymphomas of GC origin. In BL, the t(8;14) translocation is found in approximately 80% of all patients.^449^ In DLBCL, MYC overexpression is shown in 30–50% of patients.^450,451^ *MYC* translocations preferentially occur in GCB-DLBCL over ABC-DLBCL (17.7% vs. 6.7%).^450^ A high level of MYC is associated with a low treatment response and poor prognosis in DLBCL patients treated with R-CHOP and may also lead to an increased relapse rate in the central nervous system.^452,453^

Studies have shown the potential effect of MYC-associated agents, including targeting cell-cycle-associated vulnerabilities, transcription, RNA processing and turnover, ribosome biogenesis and translation, as well as MYC-induced metabolic perturbations.^454^ The mitotic spindle-regulatory kinases Aurora-A and Aurora-B are both overexpressed in MYC-associated B-cells, and Aurora-A promotes the stabilization of MYC and MYCN.^455,456^ The targeted drugs and clinical trials related to specific oncogenes and proteins are shown in Table 7. A phase 1 trial (NCT01897012) of alisertib combined with romidepsin in relapsed or refractory NHLs is ongoing.

**Table 7:** Targeted drugs and clinical trials related to specific oncogenes and proteins

Drug	Disease	Trail name	Phase	Status	ORR/CR	NCT#	Reference
*MYC*							
*Alisertib*	*a selective Aurora-A inhibitor*						
Alisertib, romidepsin	Relapsed or refractory NHLs	Alisertib and romidepsin in treating patients with relapsed or refractory B-cell or T-cell lymphomas	1	Completed	NA	NCT01897012	–
*BCL-2*							
*Navitoclax*	*A BCL-2, BCL-XL, and BCL-w inhibitor*						
Navitoclax, rituximab	Lymphoid cancers	Safety study of navitoclax in combination with rituximab in lymphoid cancers	1	Active, not recruiting	–	NCT00788684	–
*Venetoclax*	*A highly selective BH3 mimetic*						
Venetoclax	relapsed or refractory NHLs/CLL	A phase 1 study evaluating the safety and pharmacokinetics of venetoclax in subjects with relapsed or refractory non-Hodgkin’s lymphoma and chronic lymphocytic leukemia	1	Active, not recruiting	MCL, 75%/21%; FL, 38%/14%; DLBCL, 18%/12%; MZL, 67%/0%	NCT01328626	^472^
Venetoclax, bendamustine, rituximab	Relapsed or refractory FL	A study evaluating the safety and efficacy of venetoclax plus bendamustine and rituximab in comparison with bendamustine plus rituximab or venetoclax plus rituximab in participants with relapsed and refractory FL	2	Completed	Venetoclax, rituximab, 32.7%/13.2%; venetoclax, BR, 45.1%/27.5%; BR, 51%/23.5%	NCT02187861	–
Venetoclax, ibrutinib	MCL	Study of venetoclax combined with ibrutinib in subjects with mantle cell lymphoma (SYMPATICO)	3	Active, not recruiting	–	NCT03112174	–
Venetoclax, RO6870810, rituximab	Relapsed or refractory DLBCL/high-grade B-cell lymphoma	A study to evaluate safety, pharmacokinetics, and clinical activity of combination of venetoclax and RO6870810, With or without rituximab, in participants with relapsed or refractory DLBCL and high-grade B-cell lymphoma	1	Active, not recruiting	–	NCT03255096	–
Venetoclax, R-CHOP/G-CHOP	B-NHLs	A safety and pharmacokinetics study of venetoclax in participants with non-Hodgkin’s lymphoma	1/2	Completed	Phase 1 part: venetoclax, R-CHOP, 87.5%/79.2%; venetoclax, G-CHOP, 87.5%/78.1%	NCT02055820	^473^
Venetoclax, DA-R-EPOCH	Aggressive B-NHLs	Study of venetoclax plus DA-R-EPOCH for the treatment of aggressive B-cell lymphomas	1	Active, not recruiting	–	NCT03036904	–
*TP53*							
*Idasanutlin*	*A potent and selective MDM2 antagonist*						
Idasanutlin, obinutuzumab/rituximab, venetoclax	Relapsed or refractory FL/DLBCL	A study of obinutuzumab in combination with idasanutlin and venetoclax in participants with relapsed or refractory follicular lymphoma or rituximab in combination with idasanutlin and venetoclax in participants with relapsed or refractory diffuse large B-cell lymphoma	1/2	Active, not recruiting	–	NCT03135262	–
*Selinexor*	*An inhibitor of exportin 1*						
Selinexor	Advanced hematological cancer	Safety study of the selective inhibitor of nuclear export selinexor in patients with advanced hematological cancer	1	Completed	31%/6%	NCT01607892	^489^
Selinexor, chemotherapy	Advanced B-NHLs	Selinexor plus chemotherapy in treating patients with advanced B-cell non-Hodgkin’s lymphoma	1/2	Recruiting	–	NCT03147885	–
*ALK*							
*Crizotinib*	*The first-generation ALK tyrosine kinase inhibitor*						
Crizotinib	Relapsed ALK^+^ lymphomas	Pilot study of crizotinib in relapsed ALK^+^ Lymphomas	2	Recruiting	–	NCT02419287	–
*Brigatinib*	*The second-generation ALK tyrosine kinase inhibitor*						
Brigatinib	Relapsed or refractory ALK^+^ ALCL	Brigatinib in relapsed or refractory ALK^+^ anaplastic large cell lymphoma	2	Recruiting	–	NCT03719898	–
*Lorlatinib*	*The third-generation ALK tyrosine kinase inhibitor*						
Lorlatinib	Relapsed ALK^+^ lymphoma	A study of oral lorlatinib in patients with relapsed ALK^+^ lymphoma (CRU3)	2	Recruiting	–	NCT03505554	–

NA: ORR or CR are not available on the clinicaltrials.gov or from the published article although the trial has been completed

*R-CHOP* rituximab, cyclophosphamide, doxorubicin, vincristine, prednisolone, *G-CHOP* obinutuzumab, cyclophosphamide, doxorubicin, vincristine and prednisone, *DA-R-EPOCH* rituximab, dose-adjusted etoposide, prednisone, vincristine, cyclophosphamide, and doxorubicin

### BCL-2

The BCL-2 family of proteins regulates the intrinsic pathway of mitochondrial apoptosis^457^ and can be divided into three groups: anti-apoptotic proteins (BH1-4 domains), multi-BH domain pro-apoptotic proteins (BH1-3 domains), and BH3-only pro-apoptotic proteins. The t(14;18)(q32;q21) translocation is a common type of *BCL-2* translocation.^458^ Mutated *BCL-2* affects cells in several aspects, such as proliferation, apoptosis, angiogenesis, and metastasis, resulting in the development of hematological malignancies.^459,460^ *BCL-2* translocation is the major hallmark of FL (>80% of samples); it occurs in bone marrow pre-B cells and leads to high BCL-2 protein expression.^461^ Chromosome 18q21 amplification leads to BCL-2 overexpression and is observed in patients with MCL.^462^ BCL-2 overexpression is also detected in approximately 30% of DLBCL.^463^ The term double-hit lymphoma (DHL) refers to a subset of DLBCLs that present concurrent rearrangements of *MYC* and *BCL-2* (sometimes *BCL-6*).^464^ DHL is present in 5–10% of DLBCL and is mostly classified as the GCB subtype, with highly aggressive clinical behavior and poor response to frontline regimens.^465,466^ The term double-expressor lymphoma (DEL) refers to a subset of DLBCLs that show the coexpression of MYC (>40%) and BCL2 (>50%) by immunohistochemistry in the absence of chromosomal translocations. DEL is present in 25–30% of DLBCL and is mostly classified as the ABC subtype, which is also associated with poor clinical outcomes.^466,467^

ABT-737, which binds to BCL-2, BCL-XL, and BCL-w with high affinity, had promising preclinical effects in CLL.^468,469^ Navitoclax (ABT-263), the orally available derivative of ABT-737,^470^ was shown to provoke transient thrombocytopenia in phase 2 trials of patients with B-NHLs due to the importance of BCL-XL for the survival of platelets.^471^ A phase 1 trial of navitoclax combined with rituximab (NCT00788684) in lymphoid cancers is ongoing. Venetoclax (ABT-199), a highly selective BH3 mimetic, is designed to treat lymphomas with *BCL-2* translocations. A phase 1 trial (NCT01328626) of venetoclax in relapsed or refractory NHLs showed an ORR of 75% (CR 21%) in MCL, an ORR of 38% (CR 14%) in FL, an ORR of 18% (CR 12%) in DLBCL and an ORR of 67% (CR 0%) in MCL.^472^ A phase 2 study (NCT02187861) of venetoclax plus rituximab vs. venetoclax plus BR in patients with relapsed or refractory FL was completed. The results showed an ORR of 32.7% (CR 13.2%) in the venetoclax plus rituximab group, an ORR of 45.1% (CR 27.5%) in the venetoclax plus BR group, and an ORR of 51% (CR 23.5%) in the BR group. Many clinical trials on combination therapy of venetoclax and chemotherapy or other targeted agents are active. In MCL, a phase 3 randomized, double-blind study (NCT03112174) to compare the efficacy and safety of the combination of ibrutinib and venetoclax vs. ibrutinib and placebo is ongoing. A phase 1 study (NCT03255096) on the combination of RO6870810 (a bromodomain inhibitor) and venetoclax, with or without rituximab, in relapsed or refractory DLBCL and high-grade B-cell lymphoma is ongoing. To test the effect of venetoclax in combination with chemotherapy, a study (NCT02055820) of venetoclax in combination with R-CHOP or obinutuzumab plus CHOP (G-CHOP) in previously untreated DLBCL was performed, and the results demonstrated an ORR of 87.5% (CR 79.2%) in the venetoclax plus R-CHOP group and an ORR of 87.5% (CR 78.1%) in the venetoclax plus G-CHOP group. Moreover, 87.5% of DEL patients achieved CR.^473^ Another phase 1 trial (NCT03036904) of venetoclax plus DA-R-EPOCH is also active for aggressive B-NHLs.

### BCL-6

*BCL-6* was initially discovered as an oncogene in B-NHLs. The BCL6 protein is an evolutionarily conserved zinc finger transcription factor with an N-terminal broad-complex, tram track and bric-a-brac/Pox virus and zinc finger (BTB/POZ) domain and functions as a transcriptional repressor.^474^ Transcription factors, transcriptional corepressors, signaling mediators, and catalytic enzymes can be regulated by BCL-6. Studies have shown that BCL-6 overexpression inhibits reactive oxygen species (ROS) generation and represses the apoptosis induced by chemotherapy in B-NHL cells.^475,476^ Similar to BCL-2, BCL-6 is the key factor for the development and maintenance of GCs within lymphoid follicles. Once GC B-cells begin their differentiation into memory B-cells and PCs with an appropriate affinity for the inciting antigen, BCL-6 will be phosphorylated and subsequently degraded by the proteasome.^476^ Moreover, BCL-6 regulates T_FH_ cell differentiation.^477,478^ *BCL-6* translocations are found in 40% of DLBCL, 48% of nodular lymphocyte-predominant Hodgkin lymphoma, and 5-10% of FL.^445,479,480^ ABC-DLBCL patients have more *BCL6* translocations than GCB-DLBCL patients (24% vs. 10%). In T-NHLs, BCL-6 is detectable in some types of PTCL, especially ALK^+^ ALCL and lymphomas derived from T_FH_ cells, particularly AITL.^481,482^ Oncogene addiction is switched to *BCL-2* and *BCL-XL* in the context of BCL-6 inhibition.^483^ To solve this problem, a combined treatment of RI-BPI (a BCL-6 inhibitor) and ABT-737 might be a choice but needs more experimental verification.

### p53

The p53 transcription factor plays an important role in regulating cell survival by activating gene transcription that is involved in apoptosis and other biological functions.^484^ Notably, p53 can interact with the BCL-2 pathway by directly and indirectly regulating the anti-apoptotic activity of the BCL-2 family of proteins.^485^ With a negative feedback response, the E3 ubiquitin ligase MDM2 can bind p53 for degradation, maintaining a low expression level of p53 under normal conditions.^486^ The dysregulation of p53 can be found in many types of lymphomas, including DLBCL (16–30%), MCL (21–45%), FL (9–29%), and MZL (8–12%).^458^ It is often regarded as an independent prognostic factor for poor outcomes and a signal for chemotherapy resistance.^487^ Targeting p53 can potentially restart apoptosis and trigger cell death. Idasanutlin (RG7388), a potent and selective MDM2 antagonist, when combined with obinutuzumab and venetoclax, showed significant antitumor activity in xenograft models.^488^ A phase 1/2 trial (NCT03135262) of idasanutlin in combination with rituximab and venetoclax in relapsed or refractory DLBCL patients is ongoing. Selinexor, an inhibitor of exportin 1 (XPO1), inhibits the nuclear export of p53 and restores p53 nuclear localization. A phase 1 study (NCT01607892) of selinexor showed an ORR of 31% (CR 6%) in advanced NHLs.^489^ A study of selinexor combined with chemotherapy (NCT03147885) in advanced B-NHLs is ongoing.

### ALK

ALK^+^ ALCL is characterized by the expression of ALK fusion proteins.^464^ The major type of *ALK* fusion is the t(2;5)(p23;q35) translocation, which is detectable in approximately 75–85% of ALK^+^ ALCL.^490,491^ All fusion proteins can activate the downstream oncogenic transcription factor STAT3 and promote proliferation and growth in cancer cells.^492^ Many inhibitors are clinically available for targeting ALK tyrosine kinase activity, including crizotinib as a first-generation agent, ceritinib and brigatinib as second-generation agents and lorlatinib as a third-generation agent.^493^ The study of crizotinib in relapsed and refractory ALK^+^ lymphomas showed an ORR of 90.5%, with a 2-year PFS of 63.7% and a 2-year OS of 72.7%.^494^ Thus, a phase 2 trial of crizotinib (NCT02419287) and brigatinib (NCT03719898) in relapsed ALK^+^ lymphomas is ongoing. Moreover, because of acquired resistance from first- and second-generation agents, a phase 2 study (NCT03505554) to define the ORR of lorlatinib in patients with ALK^+^ lymphomas resistant or refractory to ALK inhibitors is ongoing.

## Conclusions

With the understanding of the biological function of surface markers, signaling transduction pathways, and epigenetic modulations as well as the orchestration of the microenvironment with lymphoma cells in lymphoma progression, many novel agents and immune therapeutic strategies have been developed. These therapies enable clinicians to perform precision medicine and significantly improve the prognosis of patients. However, many questions remain to be answered, such as treatment scheduling, optimized dosage and combinations with other agents. The identification of potential biomarkers that can predict the clinical responses and toxicities of these targeted therapies is challenging. In conclusion, mechanism-based targeted therapy is a promising strategy to eventually make lymphoma a curable disease.

## Supplementary information

Abbreviations-Revised
