# Supplementary material for: Advances in targeted therapy for malignant lymphoma
Source: Signal Transduct Target Ther. 2020 Mar 6;5:15. doi: 10.1038/s41392-020-0113-2 (PMC7058622; doi:10.1038/s41392-020-0113-2)
Supplement: Supplementary file 1 — Abbreviations-Revised [file 41392_2020_113_MOESM1_ESM.doc]

**Abbreviations**

Hodgkin's lymphoma (HL), non-Hodgkin's lymphoma (NHL), B-cell NHLs (B-NHLs), T-cell NHLs (T-NHLs), natural killer (NK), chimeric antigen receptor T-cells (CAR-T), overall survival (OS), adverse events (AEs), monoclonal antibodies (mAbs), antibody-dependent cellular cytotoxicity (ADCC), complement-dependent cytotoxicity (CDC), Fc gamma receptor (FcγR), chronic lymphocytic leukemia (CLL), follicular lymphoma (FL), progression-free survival (PFS), overall response rate (ORR), Radioimmunotherapy (RIT), Yttrium-90 (^90^Y), partial response (PR), complete response (CR), autologous stem cell transplantation (ASCT), B-cell receptor (BCR), diffuse large B-cell lymphoma (DLBCL), small lymphocytic lymphoma (SLL), rituximab, cyclophosphamide, doxorubicin, vincristine, prednisolone (R-CHOP), event-free survival (EFS), antibody-drug conjugates (ADCs), rituximab, cyclophosphamide, vincristine, and prednisolone (R-CVP), hairy cell leukemia (HCL), tumor necrosis factor receptor (TNFR), nuclear factor-κB (NF-κB), anaplastic large cell lymphoma (ALCL), primary mediastinal B-cell lymphoma (PMBCL), peripheral T-cell lymphoma (PTCL), mycosis fungoides (MF), adult T-cell leukemia/lymphoma (ATLL), brentuximab vedotin (BV), monomethylauristatin E (MMAE), duration of response (DoR), BV, doxorubicin, vinblastine, and dacarbazine (A+AVD), doxorubicin, bleomycin, vinblastine, and dacarbazine (ABVD), cyclophosphamide, doxorubicin and prednisone (CHP), Sézary syndrome (SS), cytomegalovirus (CMV), mantle cell lymphoma (MCL), bendamustine and rituximab (BR), rituximab, cyclophosphamide, doxorubicin and prednisone (R-CHP), ifosfamide, carboplatin, etoposide (ICE), dexamethasone, high dose cytarabine, cisplatin(DHAP), Burkitt lymphoma (BL), Waldenström's macroglobulinemia (WM), antibody radionuclide conjugate (ARC), C-C chemokine receptor type 4 (CCR4), cutaneous T-cell lymphoma (CTCL), carmustine, etoposide, cytarabine, melphalan chemotherapy (BEAM), multiple myeloma (MM), NK/T-cell lymphoma (NKTCL), rituximab plus ICE (R-ICE), killer-cell immunoglobulin-like receptors (KIRs), Bispecific T cell Engager (BiTEs), spleen tyrosine kinase (SYK), Bruton’s tyrosine kinase (BTK), phosphoinositide 3-kinase (PI3K), mammalian target of rapamycin (mTOR), Janus kinase-signal transducer and activator of transcription (JAK-STAT), ubiquitin-proteasome pathway (UPP), T-cell receptor (TCR), immunoreceptor tyrosine-based activation motifs (ITAMs), phospholipase C γ2 (PLCγ2), zeta-chain-associated protein kinase 70 (ZAP-70), the activated B cell-like subtype of DLBCL (ABC-DLBCL), germinal center B-cell-like (GCB), monomorphic epitheliotropic intestinal T-cell lymphomas (MEITL), PTCL, not otherwise specified (PTCL-NOS), angioimmunoblastic T-cell lymphoma (AITL), B-cell adaptor for PI3K (BCAP), phosphatidylinositol 4,5-bisphosphate (PIP2), phosphatidylinositol 3,4,5-trisphosphate (PIP3), 1,4,5-trisphosphate (IP3), diacylglycerol (DAG), nuclear factor of activated T cells (NFAT), protein kinase Cβ (PKCβ), mitogen-activated protein kinases (MAPK), lymphoplasmacytic lymphoma (LPL), anaplastic lymphoma kinase (ALK), γ-secretase inhibitors (GSIs), T-cell lymphoblastic lymphoma (T-LBL), splenic marginal zone lymphoma (SMZL), inhibitor of NF-κB (IκB), IκB kinase (IKK), B-cell associated kinases (BAKs), CARD11-BCL-10-MALT1 (CBM), mucosa-associated lymphoid tissue (MALT), NEDD8-activating enzyme (NAE), gemcitabine, dexamethasone, and cisplatin (GDP), bortezomib, rituximab, cyclophosphamide, doxorubicin, and prednisone (VR-CAP), DNA methyltransferases (DNMTs), rituximab and GDP (R-GDP), International Prognostic Index (IPI), decitabine plus CHOP (D-CHOP), histone deacetylase 3 (HDAC3), isocitrate dehydrogenase (IDH), 2-hydroxyglutarate (2HG), Enhancer of zeste homolog 2 (EZH2), histone-lysine N-methyltransferase 2D (KMT2D), histone deacetylase inhibitor (HDACi), histone acetyltransferases (HATs), dexamethasone, ifosfamide, cisplatin, etoposide (DICE), vinorelbine, liposomal doxorubicin, dexamethasone and thalidomide (VDDT), cyclophosphamide, doxorubicin, vincristine, etoposide, and prednisone (CHOEP), cyclophosphamide, prednisone, thalidomide (CPT), prednisone, etoposide, thalidomide (PET), prednisone, etoposide, cyclophosphamide, methotrexate (PECM), programmed cell death-1 (PD-1), tumor-infiltrating lymphocytes (TILs), Hodgkin and Reed-Sternberg (HRS), soluble PD-L1 (sPD-L1), rituximab, gemcitabine, oxaliplatin (R-GMox), rituximab, dose-adjusted etoposide, prednisone, vincristine, cyclophosphamide, and doxorubicin (DA-R-EPOCH), antigen-presenting cells (APCs), T-cell immunoglobulin and ITIM domains (TIGIT), T effector memory cell (TEMs), indoleamine 2,3-dioxygenase (IDO), mesenchymal stromal cells (MSCs), myeloid-derived suppressor cells (MDSCs), cytokine release syndrome (CRS), cord blood (CB), double-hit lymphoma (DHL), double-expressor lymphoma (DEL), obinutuzumab plus CHOP (G-CHOP), reactive oxygen species (ROS)
